# Synthesis of Thiazolo[5,4-*f*]quinazolin-9(8*H*)-ones as Multi-Target Directed Ligands of Ser/Thr Kinases

**DOI:** 10.3390/molecules21050578

**Published:** 2016-04-30

**Authors:** Damien Hédou, Julien Godeau, Nadège Loaëc, Laurent Meijer, Corinne Fruit, Thierry Besson

**Affiliations:** 1 Normandie Univ; Uni Rouen; INSA; CNRS, COBRA, UMR 6014, 76000 Rouen, France; d.hedou@laposte.net (D.H.); juliengoorga@hotmail.fr (J.G.); corinne.fruit@univ-rouen.fr (C.F.); 2 Protein Phosphorylation & Human Disease group, Station Biologique, 29680 Roscoff, France; Nadege.Loaec@univ-brest.fr; 3 Manros Therapeutics, Centre de Perharidy, 29680 Roscoff, France; meijer@manros-therapeutics.com

**Keywords:** thiazolo[5,4-*f*]quinazolin-9(8*H*)-ones, multi-target-directed ligand, protein kinases, microwave-assisted synthesis, CDK5, GSK-3, CLK1, CK1, DYRK1A.

## Abstract

A library of thirty novel thiazolo[5,4-*f*]quinazolin-9(8*H*)-one derivatives belonging to four series designated as **12**, **13**, **14** and **15** was efficiently prepared, helped by microwave-assisted technology when required. The efficient multistep synthesis of methyl 6-amino-2-cyano- benzo[*d*]thiazole-7-carboxylate (**1**) has been reinvestigated and performed on a multigram scale. The inhibitory potency of the final products against five kinases involved in Alzheimer’s disease was evaluated. This study demonstrates that some molecules of the **12** and **13** series described in this paper are particularly promising for the development of new multi-target inhibitors of kinases.

## 1. Introduction

Major human diseases such as cancer, neurodegenerative disorders and cardiovascular diseases have been closely associated with the deregulation of kinases [1,2,3]. Consequently, protein kinases represent pertinent targets for academic and industrial chemists searching for kinase inhibitors as potential new therapeutic agents [4,5,6]. Most kinases phosphorylate both serine and threonine residues, others phosphorylate tyrosines, and a small number phosphorylate all three amino acids (dual-specificity kinases). Our research groups are mostly invested in the synthesis of sulfur- nitrogen heteroaromatic molecules able to modulate the activity of deregulated kinases thought to be involved in Alzheimer’s disease (AD) [7,8,9,10,11,12,13,14,15]. These five important kinases used in this study are the Ser/Thr kinases (CDK5, GSK-3, CLK1 and CK1) and the dual-specificity kinases (DYRK1A family) [16,17,18,19]. The important impact of these kinases in various key cellular regulatory mechanisms is justifying recent approaches consisting in the design of multi-target-directed ligands (MTDLs) [20,21,22,23] able to target more than one kinase. This highly pertinent therapeutic strategy may allow the development of new tools or therapies to better understand and treat patients suffering of neurodegenerative diseases.

**Figure 1:**
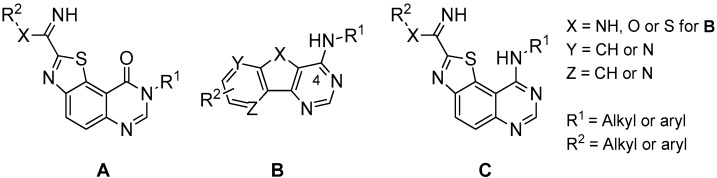
General formulae A, B and C of previously described kinase inhibitors [6,7,8,9,10,11,12,13,14].

**Figure 2:**
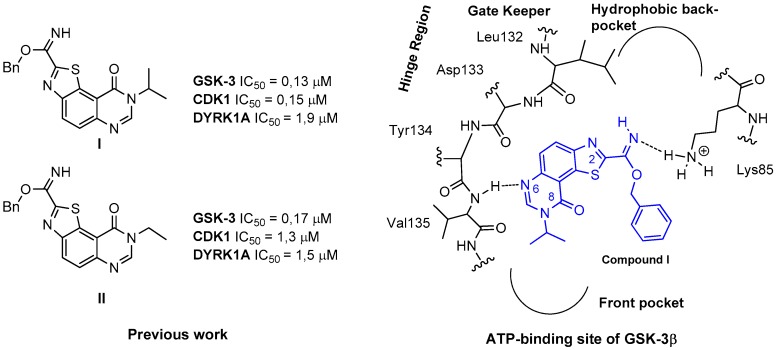
Schematic representation of the proposed binding mode of compound **I** in the ATP-binding site of GSK-3β.

In the course of our work, we previously described the synthesis of a small library of 8*H*-thiazolo[5,4-*f*]quinazolin-9(8*H*)-ones (**A** in Figure 1) as dual CDK1/GSK-3 kinases inhibitors. Brief studies of their inhibitory potency were realized with a small panel of kinases and showed two compounds (**I** and **II** in Figure 2) with a micromolar range inhibitory effect on CDK1 and GSK-3 [7,8]. More recently, the synthesis and the kinase inhibitory potency of various *N*-arylbenzothieno[3,2-*d*]pyrimidin-4-amines, and their pyrido and pyrazino analogues (**B** in Figure 1), have been published. These original heteroaromatics provide new means to target and inhibit some of the abovementioned kinases in the nanomolar range [9,10,11].

The overall pharmaceutical interest of all these compounds encouraged us to conceive new series of thiazolo[5,4-*f*]quinazolines substituted in position 4 of the pyrimidine ring by an aromatic amine and by carboximidamide or amidine groups in position 2 of the thiazole moiety [6,12,13,14,15] (see general formula **C** in Figure 1). These compounds were conceived as 6,6,5-tricyclic homologues of the basic 4-aminoquinazoline pharmacophore which is present in approximately 80% of ATP-competitive kinase inhibitors that have received approval for the treatment of cancer [4,5]. Five of the novel thiazolo[5,4-*f*]quinazoline derivatives prepared displayed single-digit nanomolar or subnanomolar IC_50_ values and are among the most potent and selective DYRK1A/1B inhibitors disclosed to date [13,14,15].

Returning to our initial work [7,8] and extending the list of targeted kinases, we discovered that compounds **I** and **II** exhibit micromolar IC_50_ values against DYRK1A (Figure 2). This result suggested the possibility to extend the scope of pertinent kinases that these ligands are able to target.

The results of various docking studies realized to understand the structure-activity relationships (SARs) are represented in Figure 2 and concern the ATP-binding site of GSK-3β [7,8,24]. They suggest that the unencumbered nitrogen in position 6 of the tricyclic core can form a hydrogen bond with the backbone NH-residue of Val135 in the hinge segment. This region of kinases ATP-binding site is considered as a critical H-bonded system with the majority of inhibitors that have been published to date. As described in Figure 2, a benzylcarbimidate function may form polar interaction between nitrogen atom of the imidate and the ammonium group of Lys85. In this hypothesis a bulky substituent at the R^2^ position may not fit into the hydrophobic back-pocket of GSK-3β. This region of kinases ATP-binding site is known to not be conserved among kinases and can thus be used to gain affinity as well as selectivity.

**Figure 3:**
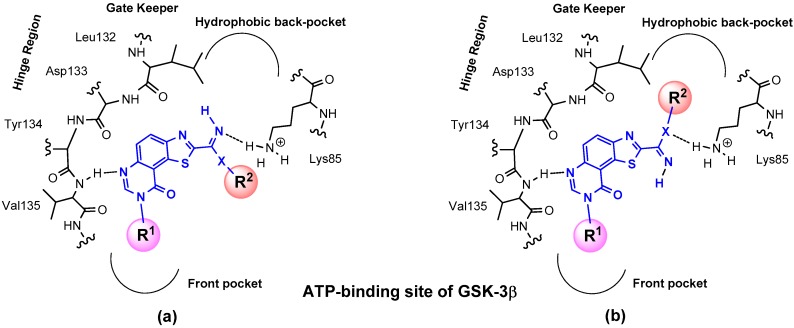
Suggested position of envisioned molecules in the targeted binding sites (example of the ATP-binding site of GSK-3β: (**a**) *cis-* spatial position of R^1^ and R^2^ or (**b**) *trans-*spatial position of R^1^ and R^2^.

As depicted in Figure 3, modulating the size and/or the nature of R^1^ and R^2^ may have an effect on the affinity of the ligands by allowing *cis-* or *trans-*spatial positions of these groups in the targeted binding sites. In the latest case, R^2^ group will be able to fit into the back-pocket of kinases and concomitantly, the R^2^ group present on *N*^8^ may also influence the position of the inhibitor into the enzyme site.

Considering all these facts, the synthetic route to the thiazolo[5,4-*f*]quinazolin-9(8*H*)-one scaffold was optimized with the aim of modulating the R^1^ and R^2^ groups. This was expected by substituting the position 8 of the pyrimidine ring with various alkyl and aryl groups and by introducing various alkyl substituents on the carbimidate groups in position 2 of the thiazole moiety. Concerning this last point, the choice of the aliphatic chains for R^1^ was inspired by previous results (see compounds C in Figure 1) showing that small size groups can help to enhance the inhibitory activity against kinases [13].

This paper describes the development of a simple and reliable method allowing the preparation of a library of new thiazolo[5,4-*f*]quinazolin-9(3*H*)-ones for which interesting multi-target kinase inhibitory activities were observed. The main part of the chemistry described in this paper was achieved under microwave irradiation as a continuation of our global strategy consisting in the design of appropriate reagents and techniques offering operational, economic, and environmental benefits over conventional methods [25,26,27].

## 2. Results and Discussion

### 2.1. Chemistry

**Scheme 1:**
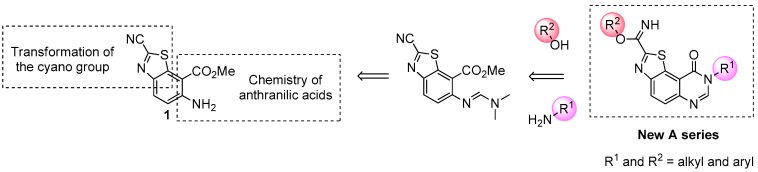
Envisioned transformations of **1** for synthesis of novel compounds of series **A**.

The target molecules were thiazolo[5,4-*f*]quinazolin-9(8*H*)-ones substituted in position *N*^8^ (which corresponds to position 4 of the pyrimidine ring) by an aliphatic chain or an aromatic substituent (Scheme 1). In order to reach an efficient route to these various 8-substituted thiazolo[5,4-*f*]quinazolin-9(8*H*)-ones, a rational multistep synthesis of a novel polyfunctionalized benzothiazole (see **1** in Scheme 1) has been developed [28,29,30]. This molecular system was conceived to be a versatile efficient precursor to various target molecules. The presence of the carbonitrile function in position 2 of the thiazole ring should allow easy access to (alkyl)carbimidate function and the *ortho*-aminobenzoïc ester part should access to the target *N*^8^-substituted pyrimidin-4-one derivatives.

**Scheme 2:**
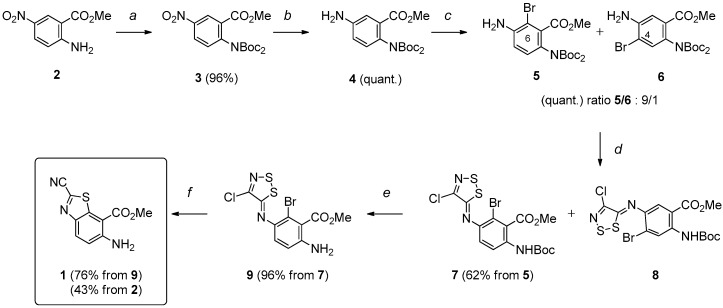
Multistep synthesis of the key benzothiazole **1**. *Reagents and conditions:* (**a**) Boc_2_O (2.3 equiv), DMAP (1.0 equiv), Et_3_N (1.0 equiv), THF, r.t., 5 h; (**b**) HCO_2_NH_4_ (5.0 equiv), Pd/C (10%), EtOH, 78 °C (μw), 15 min; (**c**) NBS (1.0 equiv), DMF, −10 °C, 3 h; (**d**) Appel salt (1.2 equiv), Py. (2.3 equiv), CH_2_Cl_2_, r.t., 3 h; (**e**) TFA, CH_2_Cl_2_, r.t., 2 h; (**f**) CuI (1.0 equiv), Py., 115 °C (μw), 30 min.

Based on our previous studies [25,26,27], the synthesis of the key intermediate **1** was revised and optimized in six steps according to the procedure depicted in Scheme 2. *N*^2^-Protection of methyl 5-nitroanthranilate (**2**) [31] provided methyl 2-[di(*tert*-butoxycarbonyl)amino]-5-nitrobenzoate (**3**), which was reduced by treatment with ammonium formate in the presence of a catalytic amount of 10% palladium charcoal. The resulting aromatic amine (**4**) was treated with *N*-bromosuccinimide (NBS) in DMF to give **5** and **6** (ratio **5**/**6**: 9/1) in a quantitative yield. The mixture of *ortho*-bromo anilines **5** and **6** was reacted with Appel’s salt (4,5-dichloro-1,2,3-dithiazolium chloride) to give intermediate imino-1,2,3-dithiazoles **7** and **8** which were separated and purified by flash-column chromatography on silica gel. The intermediate imine **7** was transformed into the target methyl 6-amino-2-cyanobenzo[*d*]thiazole-7-carboxylate (**1**) after *N*^2^-deprotection (giving **9**, 96%) and microwave-assisted copper-mediated cyclization. This synthetic route allowed an efficient and reproducible preparation of **1**, in a good overall yield of 43%, helped in some steps by microwave-assisted heating. In terms of efficiency, 20 g of 2-methyl 5-nitroanthranilate (**2**) may lead to 6 g of polyfunctionalized benzo[*d*]thiazole **1**.

**Scheme 3:**
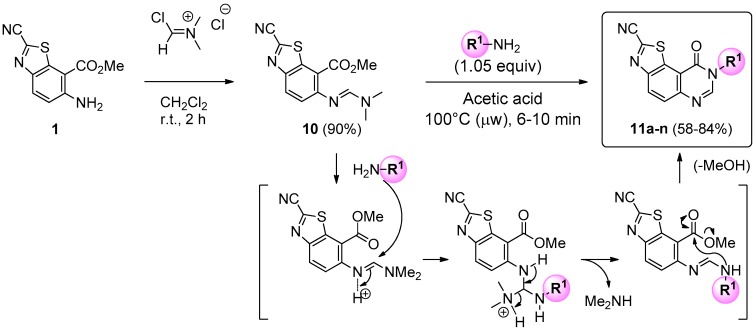
Synthesis of series **11** compounds and suggested mechanism of cyclization after attack of the primary amine. For yields see Table 1.

Treatment of **1** with 1.5 equiv of Vilsmeier-Haack reagent in dichloromethane at room temperature gave (*E*)-methyl 2-cyano-6-([(dimethylamino)methylene]amino)benzo[*d*]thiazole-7-carboxylate (**10**) in excellent yield (90%). This efficient synthesis can be performed at the multi-gram scale, enabling preparation of several grams of the key precursor **10**. The aforementioned formimidamide was heated at 100 °C under microwave irradiation, in the presence of 1.05 equiv of appropriate amines, in acetic acid. After irradiation times of 6 to 10 min, cyclization of the pyrimidin-4-one part of the tricyclic product allowed access to the expected *N*^8^-substituted-9-oxo- 8,9-dihydrothiazolo[5,4-*f*]quinazoline-2-carbonitriles (series **11**) in moderate to good yields (58%–84%). Considering previous kinetics studies [32], it may be assumed that the mechanism of cyclization occurred via a first attack of the amine on the activated carbon of the amidine. The intermediate triamine species released dimethylamine and cyclized into the expected quinazolin-4-one derivatives (Scheme 3).

**Scheme 4:**
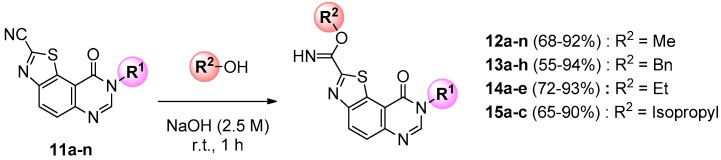
General synthesis of carbimidates **12**, **13**, **14** and **15** from **11** series (for yields see Table 2).

At the last stage of the synthesis, transformation of the carbonitrile group into a methylcarbimidate was realized by stirring compounds **11a**–**n** with sodium hydroxide (2.5 M in water) in methanol at room temperature for 1 h (Scheme 4). Methyl 9-oxo-8,9-dihydrothiazolo[5,4-*f*]quinazoline-2-carbimidates (**12a**–**n**) were thus obtained in good to excellent yields (Table 2). In the course of SAR studies, libraries of various carbimidates were extended to ethyl, isopropyl, and benzyl derivatives using the same procedure and the appropriate alcohol (Scheme 4).

The synthesis of forty-four 8-susbtituted thiazolo[5,4-*f*]quinazolin-9(8*H*)-one derivatives was performed with success. This process was helped by the use of methyl 6-amino-2-cyano- benzo[*d*]thiazole-7-carboxylate (**1**) [27], a molecular platform conceived to be a versatile and efficient precursor to various target molecules. Note that microwave heating was mainly performed at atmospheric pressure in a controlled multimode cavity with a microwave power delivery system ranging from 0 to 1200 W. Concerning the technical aspects, open-vessel microwave experiments have some advantages, such as the possibility of easier scale-up and the use of common laboratory glassware.

### 2.2. Biological Studies

Products of series **11a**–**c**, **12a**–**n**, **13a**–**h**, **14a**–**e** and **15a**–**c** were tested in five different *in vitro* kinase assays (CDK5/p25 (cyclin-dependent kinase), CK1δ/ε casein kinase 1), GSK-3α/β glycogen synthase kinase 3), DYRK1A (dual-specificity, tyrosine phosphorylation regulated kinase) and CLK1 (cdc2-like kinase 1) to evaluate their inhibition potency [33,34,35,36,37]. All compounds were first tested at a final concentration of 10 μM. Compounds showing less than 50% inhibition were considered as inactive (IC_50_ >10 μM). Compounds displaying more than 50% inhibition at 10 μM were next tested over a wide range of concentrations (usually 0.01 to 10 μM), and IC_50_ values were determined from the dose-response curves (Sigma-Plot). Harmine (Table 3) is a β−carboline alkaloid known to be a potent inhibitor of DYRK1A [38]. It was also tested as positive control and its IC_50_ values were compared to those obtained for the compounds under study.

Results given in Table 3 demonstrate that none of the tricyclic derivatives prepared in this work showed significant inhibitory activity against CDK5/p25 and CK1δ/ε. Only three compounds of **11a**–**c** series are described in Table 3 and none of them showed any significant activity against the targeted kinases. These results are consistent with previous studies [7,8].

The results of the kinases inhibition potential obtained with compounds of the **14a**–**e** and **15a**–**c** series were also quite disappointing. Apart from product **14e**, which exhibits a good and selective inhibition of GSK-3 (IC_50_ = 0.029 μM) and product **15a**, which revealed a fairly good inhibition of CLK1 (IC_50_ = 0.043 μM), the IC_50_ values obtained for the other compounds of these two series (**14a**–**d**, **15b** and **15c**) were not significant.

In general, interesting and significant biological activity of the tested compounds was oriented towards three kinases of the initial panel: CLK1, DYRK1A and GSK-3α/β.

Undoubtedly, the most active molecules prepared in this study were series **12** and **13** in which the final carbimidate function was obtained after attack of methanol or benzyl alcohol. Most of these compounds from these two series showed submicromolar activities against CLK1, DYRK1A and GSK-3α/β, except product **12m** which was completely inactive (IC_50_ > 10 μM for the five kinases tested). Curiously, despite the size modification of the carbimidate substituents, similar activity profiles can be observed for the **12** and **13** series (see Table 3 in which the most significant results are underlined in grey and significant IC_50_ values in the nanomolar range written in bold). It appears that the most significant results were obtained with compounds of the **a** and **d**–**g** series in the two family products (**12** and **13**).

Results obtained with compounds **12h**–**l** (CLK1: 0.691 μM < IC_50_ < 7.9 μM), DYRK1A (0.2 μM < IC_50_ < 3.0 μM) and GSK-33 α/β (0.3 μM < IC_50_ < 1.8 μM) demonstrated the necessity to conserve a cyclic alkyl group in position 8 of the tricyclic core (**12a** and **12d**–**g**), in order to maintain good to very good affinity. The presence of basic groups in *N*^8^ (**12h**–**j)** may be correlated with an rather important decrease of affinity, in the same way as the presence of aromatic substituents in position 8 of the thiazolo[5,4-*f*]quinazolin-9(8*H*)-one core (compounds **12k**–**m**). In this last case, it appeared that the nature of the substituents present in the *para*-position of the phenyl group in *N*^8^ may influence and decrease the IC_50_ values measured for the various kinases. Compared with data obtained for compounds in the **12d**–**g** series, it seems to be the consequence to steric influence rather than electronic effects. Compounds **12b**, **12c** and **13b**, **13c** were also analyzed and although their inhibitory activity towards the three kinases (CLK1, DYRK1A and GSK-3α/β) was characterized by submicromolar values of IC_50_, their global activity was considered not significant enough to continue further studies.

Comparison of the substituents present on *N*^8^ position of **a** and **d**–**f** series shows mainly a small-size ring (e.g., cyclopropyl for **12a** and **13a** series) or its equivalent such as isopropyl (**12d** and **13d**), cyclobutyl (**12f** and **13f**) and cyclopentyl (**12e** and **13e**). The size of the cycle present in position *N*^8^ seems to be extremely important, whilst 3-, 4- and 5-membered rings exhibit the best affinity for the target kinases.

Concerning the activity against CLK1, comparison of the results obtained for compounds **12a** and **12f** (IC_50_ = 0.031 μM and 0.091 μM, respectively) with those obtained for **13a** and **13f** (IC_50_ = 0.06 μM and 0.071 μM, respectively), demonstrates that the presence of methyl or benzyl carbimidates at C^2^ may have a slightly beneficial effect on the inhibitory activity, accompanied by the same cyclopropyl substituent on *N*^8^. The same phenomenon is observed when data concerning DYRK1A and GSK-3α/β kinases are compared. The DYRK1A-IC_50_ values obtained for **12a**, **12d**–**f** and **13a**, **13d**–**f** series are mainly in the submicromolar range (0.13 μM < IC_50_ < 0.39 μM) except for compounds **12a** and **13a** (IC_50_ = 0.091 μM and 0.06 μM, respectively), **12e** and **13e** (IC_50_ = 0.072 μM and 0.062 μM, respectively) and **13**f (IC_50_ = 0.00.59 μM) for which nanomolar IC_50_ values were observed. These five products show interesting activity against DYRK1A. As described above for CLK1, the interesting results concern the fact that two different carbimidate groups gave similar affinity for the same kinase despite changes in the size of their substituents in position *N*^8^. This may suggest the existence of a spatial zone in the front pocket of the active site of the kinases which is relatively tolerant to various sizes of the molecules with 3-, 4- or 5- membered cyclolalkyl groups.

In the case of GSK-3α/β, the nanomolar values obtained for compounds **12a**, **12d**–**h** and **13a**, **13d**–**h** are spectacular and the list of compounds able to show very good affinity for the target kinase was extended to **12d**–**13d** (IC_50_ = 0.041 μM and 0.030 μM, respectively) and **12g**–**13g** (IC_50_ = 0.030 μM and 0.083 μM, respectively). For GSK-3α/β in particular, the fact that its hydrophobic back-pocket was recognized to be larger than in the case of the two other kinases (CLK1 and DYRK1A) may explain why **12d** and **12g** show very good affinity for this kinase. It may be suggested that the space available in this back-pocket allowed the molecules to shift and to have a better interaction with GSK-3α/β than in the other enzymes in which hydrophobic back-pocket are known to have a reduced size. In the same time the displacement of the tricyclic core into the site may allow the front pocket to be more tolerant and accept other substituents in *N*^8^.

**Scheme 5:**
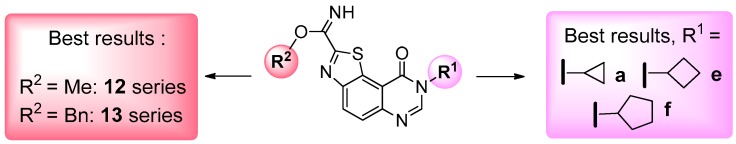
Structures of the lead compounds (nanomolar IC_50_ values) identified in this study.

All these results demonstrate that it is difficult to define any role for the various substituents located at position 8 of the thiazolo[5,4-*f*]quinazolin-9(8*H*)-one. The presence of small-sized cycloalkyl groups linked to the front pocket is favorable to the development of further SAR studies, whilst the other side of the molecule (carbimidate function) will perhaps serve to discriminate the pertinent kinases such as CLK1, DYRK1A and GSK-3α/β (Scheme 5). Although this needs to be confirmed, the results described in this study indicate clearly that the carbimidate function remains crucial for the multi-target inhibitory activity of kinases with such heterocyclic structures [13,14,15].

The first kinase CLK1 is one of the four isoforms (CLK1-4) of the cdc2-like kinase family. In humans, the highest levels of CLK1 expression were found in the brain. It was described that CLK inhibitors may alter the splicing of microtubule-associated protein tau implicated in AD and Parkinson’s disease [39]. The second kinase targeted by the compounds described in this study is DYRK1A. Evidence for the role of overexpressed DYRK1A in various neurodegenerative diseases and Down syndrome is now well established and it has become an attractive drug target for numerous research groups [40,41,42]. The third kinase inhibited by the lead molecules in this study was GSK-3α/β which has definitely gained the attention of research groups and industry. This very versatile kinase has become a key target in type II diabetes, bipolar disorder, cancer, chronic inflammatory and immune disorder and, more importantly, in neurodegenerative diseases, making this enzyme a main target for AD [43].

Because drugs focusing their activity against a single target may generate low benefits, new studies are encouraged in the direction of multi-targeting strategies. Therefore, developing molecules showing submicromolar affinities on a panel of three kinases seems to be of great interest [17]. In this sense the new thiazolo[5,4-*f*]quinazolin-9(8*H*)-one series described in this paper need to be developed for the discovery of valuable and pertinent multi-kinases inhibitors.

## 3. Materials and Methods

### 3.1. General Information

Starting materials were obtained commercially and used without further purification. All reactions were monitored by thin-layer chromatography with silica gel 60 F254 precoated aluminium plates (0.25 mm). Visualization was performed with a UV light at wavelengths of 254 and 312 nm. Purifications were conducted with a flash column chromatography system equipped with a dual UV/Vis spectrophotometer (200–600 nm), a fraction collector (176 tubes), a dual piston pump (1 to 200 mL/min, *P*_max_ = 15 bar), which allowed quaternary gradients, and an additional inlet for air purge. Melting points of solid compounds were measured with a SMP3 Melting Point instrument (STUART, Bibby Scientific Ltd, Roissy, France) with a precision of ±1.5 °C. IR spectra were recorded with a Spectrum 100 Series FTIR spectrometer (PerkinElmer, Villebon S/Yvette, France). Liquids and solids were investigated with a single-reflection attenuated total reflectance (ATR) accessory; the absorption bands are given in cm^−1^. NMR spectra (^1^H and ^13^C) were acquired at 295 K using a WP 300 spectrometer an AVANCE 300 MHz spectrometer (Bruker, Wissembourg, France) at 300 and 75.4 MHz, using TMS as an internal standard. Coupling constants *J* are in Hz, and chemical shifts are given in ppm. Signals in ^13^C spectra were assigned based on the result of ^13^C DEPT135 experiments (see Supplementary Materials). Mass spectrometry was performed by the Mass Spectrometry Laboratory of the University of Rouen. The mass spectra [ESI, EI, and field desorption (FD)] were recorded with a LCP 1er XR spectrometer (WATERS, Guyancourt, France). Microwave experiments were conducted in a commercial microwave reactor especially designed for synthetic chemistry. Start S^TM^ (Milestone S.r.l., Bergamo, Italy) is a multi-mode cavity with a microwave power delivery system ranging from 0 to 1200 W. The temperatures of the reactions were mainly monitored via contact-less infrared pyrometer which was calibrated in control experiments with a fibre-optic contact thermometer protected in a Teflon coated ceramic well inserted directly in the reaction mixture. Open vessel experiments were carried out in a 50–250 mL round bottom flask fitted with a reflux condenser. The vessel contents were stirred by means of an adjustable rotating magnetic plate located below the floor of the microwave cavity and a Teflon-coated magnetic stir bar inside the vessel. Temperature and power profiles were monitored in both cases through the EASY-Control software provided by the manufacturer (Milestone S.r.l., Bergamo, Italy). The times indicated in the various protocols are the times measured when the mixtures reached the programmed temperature after a ramp period of 2 min.

### 3.2. Chemistry

#### 3.2.1. General Procedure for the Synthesis of Carbonitriles **11a**–**n** from **10**

*(E)-Methyl 2-cyano-6-([(dimethylamino)methylene]amino)benzo[d]thiazole-7-carboxylate* (**10**): To a stirred solution of methyl 6-amino-2-cyanobenzo[*d*]thiazole-7-carboxylate (**1**, 1.00 g, 4.29 mmol) in methylene chloride (20 mL) was added Vilsmeier-Haack reagent (0.824 g, 6.44 mmol, 1.5 equiv) at room temperature. The resulting mixture was stirred at room temperature for 2 h. On completion, the crude mixture was diluted with methylene chloride (40 mL) and a saturated aqueous solution of NaHCO_3_ (40 mL). After 15 min of stirring, the product was extracted twice with methylene chloride. The combined organic layers were washed with brine and dried over MgSO_4_. Evaporation of solvent gave the amidine product **10** as a pale yellow solid (1.11 g, 90%), mp. 136–138 °C; ^1^H-NMR (DMSO-*d*_6_) δ 8.23 (d, *J* = 9.0 Hz, 1H, H_4_), 7.75 (s, 1H, CH(N)), 7.74 (d, *J* = 9.0 Hz, 1H, H_5_), 3.87 (s, 3H, OCH_3_), 3.10 (s, 3H, NCH_3_), 3.03 (s, 3H, NCH_3_); ^13^C-NMR (DMSO-*d*_6_) δ 167.1, 155.8, 154.8, 146.7, 138.3, 135.7, 128.7, 125.4, 114.8, 113.3, 52.3, 34.1; ν_max_ 2952, 2224 (ν C≡N), 1622, 1569, 1423, 1331, 1258, 1098, 1016, 831 cm^−1^; HRMS calcd for C_13_H_13_N_4_O_2_S [M + H]^+^ 289.0759 found 289.0746.

In a sealed tube, a suspension of (*E*)-methyl 2-cyano-6-([(dimethylamino)methylene]-amino)benzo[*d*]thiazole-7-carboxylate (**10**, 125 mg, 0.43 mmol) and the appropriate amine (0.45 mmol, 1.05 equiv) in acetic acid (600 μL) was irradiated under microwaves at 100 °C for 6–10 min. The solvent was removed under vacuum to provide a crude residue which was purified by flash chromatography on silica gel with methylene chloride/ethyl acetate (100:0 to 50:50, *v*/*v*) as eluent to furnish the expected 8-substituted thiazoloquinazolinone-2-carbonitrile derivatives.

*8-Cyclopropyl-9-oxo-8,9-dihydrothiazolo[5,4-f]quinazoline-2-carbonitrile* (**11a**): white solid (90.0 mg, 78%), mp. 248–250 °C; ^1^H-NMR (DMSO-*d*_6_) δ 8.63 (d, *J* = 8.7 Hz, 1H, H_4_), 8.61 (s, 1H, H_2_), 7.99 (d, *J* = 9.0 Hz, 1H, H_5_), 3.42–3.36 (m, 1H, NCH), 1.13–1.09 (m, 4H, CH); ^13^C-NMR (DMSO-*d*_6_) δ 160.2, 150.3, 149.5, 148.2, 139.2, 131.6, 129.9, 127.8, 115.4, 113.5, 29.7, 5.9 (2C); ν_max_ 3067, 2233 (C≡N), 1664, 1579, 1441, 1353, 1303, 1222, 1038, 839, 692 cm^−1^; HRMS calcd for C_13_H_9_N_4_OS [M + H]^+^ 269.0497 found 269.0487.

*8-(2-Methoxyethyl)-9-oxo-8,9-dihydrothiazolo[5,4-f]quinazoline-2-carbonitrile* (**11b**): pale beige solid (100.8 mg, 82%), mp. 204–206 °C; ^1^H-NMR (CDCl_3_) δ 8.67 (d, *J* = 9.0 Hz, 1H, H_4_), 8.62 (s, 1H, H_2_), 8.02 (d, *J* = 9.0 Hz, 1H, H_5_), 4.32 (t, *J* = 5.1 Hz, 2H, OCH_2_), 3.69 (t, *J* = 5.1 Hz, 2H, NCH_2_), 3.26 (s, 3H, OCH_3_); ^13^C-NMR (DMSO-*d*_6_) δ 159.9, 150.3, 149.8, 148.7, 139.2, 131.7, 130.1, 127.9, 115.4, 113.5, 68.8, 58.1, 46.0; ν_max_ 3098, 2932, 2891, 2237 (ν C≡N), 1656, 1582, 1352, 1111, 1013, 835, 559 cm^−1^; HRMS calcd for C_13_H_11_N_4_O_2_S [M + H]^+^ 287.0603 found 287.0605.

*8-(3-Methoxypropyl)-9-oxo-8,9-dihydrothiazolo[5,4-f]quinazoline-2-carbonitrile* (**11c**): pale beige solid (103.4 mg, 80%), mp. 128–130 °C; ^1^H-NMR (DMSO-*d*_6_) δ 8.68 (s, 1H, H_7_), 8.65 (d, *J* = 9.0 Hz, 1H, H_4_), 8.01 (d, *J* = 9.0 Hz, 1H, H_5_), 4.19 (t, *J* = 6.9 Hz, 2H, OCH_2_), 3.41 (t, *J* = 6.0 Hz, 2H, NCH_2_), 3.21 (s, 3H, OMe), 2.01 (dt, *J =* 6.9, 6.0 Hz, 2H, CH_2_); ^13^C-NMR (DMSO-*d_6_*) δ 159.0, 150.3, 149.6, 148.7, 139.2, 131.7, 129.9, 127.9, 115.5, 113.6, 69.1, 57.9, 44.6, 28.2; ν_max_ 3058, 2901, 2878, 2831, 2232 (ν C≡N), 1656, 1586, 1470, 1354, 1156, 1110, 889, 847 cm^−1^; HRMS calcd for C_14_H_13_N_4_O_2_S [M + H]^+^ 301.0759 found 301.075.

*8-(3-Isopropyl)-9-oxo-8,9-dihydrothiazolo[5,4-f]quinazoline-2-carbonitrile* (**11d**): obtained in 58% yield as a white solid (mp > 265 °C). Data supporting its chemical structure are reported in [6].

*8-Cyclopentyl-9-oxo-8,9-dihydrothiazolo[5,4-f]quinazoline-2-carbonitrile* (**11e**): beige solid (89.2 mg, 70%), mp > 265°C; ^1^H-NMR (DMSO-*d*_6_) δ 8.75 (s, 1H, H_2_), 8.66 (d, *J* = 8.7 Hz, 1H, H_4_), 8.02 (d, *J* = 8.7 Hz, 1H, H_5_), 5.13–5.06 (m, 1H, NCH), 2.22–1.61 (m, 8H, CH); ^13^C-NMR (CDCl_3_ + DMSO*-d*_6_) δ 159.0, 150.4, 147.3, 139.2, 129.8, 127.8, 113.7, 57.2, 30.0, 24.1; ν_max_ 3074, 2961, 2233 (ν C≡N), 1657, 1583, 1473, 1356, 1254, 1105, 840 cm^−1^; HRMS calcd for C_15_H_13_N_4_OS [M + H]^+^ 297.0810 found 297.0820.

*8-Cyclobutyl-9-oxo-8,9-dihydrothiazolo[5,4-f]quinazoline-2-carbonitrile* (**11f**): white solid (88.5 mg, 73%), mp > 265 °C; ^1^H-NMR (CDCl_3_) δ 8.52 (d, *J* = 8.7 Hz, 1H, H_4_), 8.40 (s, 1H, H_2_), 7.98 (d, *J* = 8.7 Hz, 1H, H_5_), 5.21–5.09 (m, 1H, NCH), 2.71–2.61 (m, 2H, CH), 2.54–2.40 (m, 2H, CH), 2.07–2.02 (m, 2H, CH); ^13^C-NMR (DMSO-*d*_6_) δ 159.7, 151.6, 148.8, 145.2, 140.3, 132.4, 130.6, 128.2, 116.1, 113.4, 51.2, 30.0 (2C), 15.6; ν_max_ 3441, 3296, 3092, 2233 (ν C≡N), 1662, 1586, 1280, 1145, 1059, 1114, 841 cm^−1^; HRMS calcd for C_14_H_11_N_4_OS [M + H]^+^ 283.0654 found 283.0660.

*8-Cyclohexyl-9-oxo-8,9-dihydrothiazolo[5,4-f]quinazoline-2-carbonitrile* (**11g**): white solid (92.4 mg, 69%), mp. 256–258 °C; ^1^H-NMR (DMSO-*d*_6_) δ 8.81 (s, 1H, H_7_), 8.65 (d, *J* = 9.0 Hz, 1H, H_4_), 8.01 (d, *J* = 9.0 Hz, 1H, H_5_), 4.78–4.71 (m, 1H, NCH), 2.0–1.21 (m, 10H, CH); ^13^C-NMR (DMSO-*d*_6_) δ 158.6, 150.3, 148.1, 147.2, 139.2, 131.9, 130.0, 127.9, 115.4, 113.6, 54.7, 31.1 (2C), 25.5 (2C), 24.6; ν_max_ 3060, 2940, 2869, 2226 (ν C≡N), 1657, 1583, 1466, 1448, 1388, 1346, 1183, 1127, 830 cm^−1^; HRMS calcd for C_16_H_15_N_4_OS [M + H]^+^ 311.0967 found 311.0966.

*8-Dimethylamino-9-oxo-8,9-dihydrothiazolo[5,4-f]quinazoline-2-carbonitrile* (**11h**): beige solid (98.0 mg, 84%), mp > 265 °C; ^1^H-NMR (DMSO-*d*_6_) δ 8.68 (d, *J* = 8.8 Hz, 1H, H_4_), 8.61 (s, 1H, H_2_), 8.03 (d, *J* = 8.8 Hz, 1H, H_5_), 3.10 (s, 6H, CH_3_); ^13^C-NMR (DMSO-*d*_6_) δ 159.0, 150.9, 150.3, 148.1, 139.4, 131.5, 130.1, 128.0, 117.1, 113.5, 44.2 (2C); ν_max_ 3080, 2966, 2887, 2231 (C≡N), 1659, 1577, 1442, 1349, 1299, 1156, 1060, 839, 696 cm^−1^; HRMS calcd for C_12_H_10_N_5_OS [M + H]^+^ 272.0606 found 272.0600.

*8-(2-Dimethylaminoethyl)-9-oxo-8,9-dihydrothiazolo[5,4-f]quinazoline-2-carbonitrile* (**11i**): white solid (99.1 mg, 77%), mp > 265 °C; ^1^H-NMR (CDCl_3_) δ 8.52 (d, *J* = 9.0 Hz, 1H, H_4_), 8.30 (s, 1H, H_7_), 7.98 (d, *J* = 9.0 Hz, 1H, H_5_), 4.22 (t, *J* = 6.0 Hz, 2H, NCH_2_), 2.72 (t, *J* = 6.0 Hz, 2H, NCH_2_), 2.30 (s, 6H, NCH_3_); ^13^C-NMR (CDCl_3_) δ 159.5, 151.3, 149.0, 148.3, 140.0, 132.3, 130.3, 128.1, 116.2, 113.2, 57.7, 45.0; ν_max_ 3072, 2949, 2826, 2781, 2235 (ν C≡N), 1667, 1585, 1465, 1354, 1151, 931, 832 cm^−1^; HRMS calcd for C_14_H_14_N_5_O_2_S [M + OH]^−^ 316.0868 found 316.0872.

*8-(2-Dimethylaminoethyl)-9-oxo-8,9-dihydrothiazolo[5,4-f]quinazoline-2-carbonitrile* (**11j**): yellow solid (114.5 mg, 78%), mp. 192–194 °C; ^1^H-NMR (CDCl_3_) δ 8.52 (d, *J* = 9.0 Hz, 1H, H_4_), 8.28 (s, 1H, H_7_), 7.97 (d, *J* = 9.0 Hz, 1H, H_5_), 4.23 (t, *J* = 5.7 Hz, 2H, NCH_2_), 3.68–3.65 (m, 4H, CH), 2.77 (t, *J* = 5.7 Hz, 2H, NCH_2_), 2.54–2.51 (m, 4H, CH); ^13^C-NMR (CDCl_3_) δ 159.7, 151.5, 149.2, 148.4, 140.3, 132.5, 130.6, 128.3, 116.3, 113.4, 67.1 (2C), 57.0, 53.9 (2C), 44.0; ν_max_ 3060, 2925, 2815, 1659, 1586, 1452, 1345, 1112, 833 cm^−1^; HRMS calcd for C_16_H_16_N_5_O_2_S [M + H]^+^ 342.1025 found 342.1026.

*8-(p-Tolyl)-9-oxo-8,9-dihydrothiazolo[5,4-f]quinazoline-2-carbonitrile* (**11k**): white solid (116.4 mg, 85%), mp > 265 °C; ^1^H-NMR (DMSO-*d*_6_) δ 8.72 (d, *J* = 9.0 Hz, 1H, H_4_), 8.67 (s, 1H, H_2_), 8.09 (d, *J* = 9.0 Hz, 1H, H_5_), 7.51 (d, *J* = 8.4 Hz, 2H, H_Ar_), 7.42 (d, *J* = 8.4 Hz, 2H, H_Ar_), 2.43 (s, 3H, CH_3_); ν_max_ 3057, 2225 (ν C≡N), 1668, 1580, 1490, 1353, 1273, 1189, 1091, 847, 835, 802, 576 cm^−1^.

*8-(4-Methoxyphenyl)-9-oxo-8,9-dihydrothiazolo[5,4-f]quinazoline-2-carbonitrile* (**11l**): white solid (33.7 mg, 46%), mp > 265 °C; ^1^H-NMR (DMSO-*d*_6_) δ 8.71 (d, *J* = 8.7 Hz, 1H, H_4_), 8.66 (s, 1H, H_2_), 8.08 (d, *J* = 8.7 Hz, 1H, H_5_), 7.56 (d, *J* = 9.0 Hz, 2H, ArH), 7.15 (d, *J* = 9.0 Hz, 2H, ArH), 3.56 (s, 3H, OCH_3_); ν_max_ 3099, 2932, 2237 (ν C≡N), 1656, 1584, 1352, 1111, 1013, 835 cm^−1^; HRMS calcd for C_17_H_11_N_4_O_3_S [M+OH]^−^ 351.0552 found 351.0548.

*8-(4-Dimethylaminophenyl)-9-oxo-8,9-dihydrothiazolo[5,4-f]quinazoline-2-carbonitrile* (**11m**): yellow solid (121.0 mg, 81%), mp. 224–226 °C; ^1^H-NMR (DMSO-*d*_6_) δ 8.70 (d, *J* = 9.0 Hz, 1H, H_4_), 8.63 (s, 1H, H_2_), 8.07 (d, *J* = 9.0 Hz, 1H, H_5_), 7.40 (d, *J* = 9.0 Hz, 2H, ArH), 6.87 (d, *J* = 9.0 Hz, 2H, ArH), 2.99 (s, 6H, NCH_3_); ν_max_ 3374, 3061, 2234 (ν C≡N), 1661, 1611, 14583, 1518, 1353, 1277, 1189, 836, 805 cm^−1^; HRMS calcd for C_18_H_14_N_5_O_2_S [M + OH]^−^ 364.0868 found 364.0872.

*8-Methyl-9-oxo-8,9-dihydrothiazolo[5,4-f]quinazoline-2-carbonitrile* (**11n**): white solid (87.5 mg, 84%), mp. 248–250 °C; ^1^H-NMR (CDCl_3_) δ 8.53 (d, *J* = 9.0 Hz, 1H, H_4_), 8.30 (s, 1H, H_2_), 7.98 (d, *J* = 9.0 Hz, 1H, H_5_), 3.77 (s, 3H, NCH_3_); ^13^C-NMR (CDCl_3_) δ 160.0, 151.5, 149.2, 147.9, 140.3, 132.2, 130.5, 128.2, 116.2, 113.3, 34.5; *ν*_max_ 2987, 2904, 2234 (ν C≡N), 1664, 1669, 1588, 1357, 1334, 1055, 841 cm^−1^; HRMS calcd for C_11_H_7_N_4_OS [M + H]^+^ 243.0341 found 243.0334.

#### 3.2.2. General Procedure for the Synthesis of Carbimidates **12a**–**n**, **13a**–**h**, **14a**–**e** and **15a**–**c** from **11a**–**n**

To a stirred solution of **11a**–**n** (0.25 mmol, 1 equiv) in appropriate alcohol (2.5 mL) was added an aqueous solution of sodium hydroxyde (2.5 M, 100 μL, 1.0 equiv) and the resulting mixture was stirred at room temperature for 1 h under argon atmosphere. The solvent was removed under *vacuum* and the crude residue was adsorbed on Celite^®^ and purified by flash chromatography on silica gel with methylene chloride/methanol (100:0 to 2:98; *v*/*v*) as eluent to furnish the desired carbimidate compound.

##### Ethyl 8-cyclopropyl-9-oxo-8,9-dihydrothiazolo[5,4-*f*]quinazoline-2-carbimidates **12a**–**n**

*Methyl 8-cyclopropyl-9-oxo-8,9-dihydrothiazolo[5,4-f]quinazoline-2-carbimidate* (**12a**): white solid (68 mg, 91%), mp. 234–236 °C; ^1^H-NMR (CDCl_3_) δ 8.94 (s, 1H, NH), 8.40 (d, *J* = 9.0 Hz, 1H, H_4_), 8.22 (s, 1H, H_7_), 7.78 (d, *J* = 9.0 Hz, 1H, H_5_), 4.04 (s, 3H, OCH_3_), 3.37–3.29 (m, 1H, NCH), 1.27–1.23 (m, 2H, CH), 1.01–1.00 (m, 2H, CH); ^13^C-NMR (CDCl_3_) δ 161.9 (2C), 160.9, 151.8, 147.2, 147.0, 133.0, 129.8, 126.7, 116.3, 54.4, 29.7, 6.6 (2C); ν_max_ 3272, 2918, 2848, 1728, 1664, 1583, 1498, 1438, 1339, 1141, 1060, 893, 837 cm^−1^; HRMS calcd for C_14_H_13_N_4_O_2_S [M + H]^+^ 301.0759 found 301.0771.

*Methyl 8-(2-methoxyethyl)-9-oxo-8,9-dihydrothiazolo[5,4-f]quinazoline-2-carbimidate* (**12b**): white solid (56.5 mg, 71%), mp. 196–198 °C; ^1^H-NMR (DMSO-*d*_6_) δ 9.46 (s, 1H, NH), 8.57 (d, *J* = 8.7 Hz, 1H, H_4_), 8.54 (s, 1H, H_7_), 7.93 (d, *J =* 8.7 Hz, 1H, H_5_), 4.31 (t, *J* = 5.1 Hz, 2H, OCH_2_), 3.98 (s, 1H, OCH_3_), 3.68 (t, *J* = 5.1 Hz, 2H, NCH_2_), 3.26 (s, 3H, OCH_3_); ^13^C-NMR (DMSO-*d*_6_) δ 160.6, 159.9, 159.0, 150.9, 149.0, 147.6, 131.7, 129.6, 126.9, 115.9, 68.9, 58.1, 54.1, 45.9; ν_max_ 3271, 2914, 1651, 1587, 1444, 1340, 1111, 1065 cm^−1^; HRMS calcd for C_14_H_15_N_4_O_2_S [M + H]^+^ 319.0865 found 319.0880.

*Methyl 8-(3-methoxypropyl)-9-oxo-8,9-dihydrothiazolo[5,4-f]quinazoline-2-carbimidate* (**12c**): white solid (63.0 mg, 76%), mp. 168–170 °C; ^1^H-NMR (DMSO-*d*_6_) δ 9.45 (s, 1H, NH), 8.59 (s, 1H, H_7_), 8.54 (d, *J* = 8.7 Hz, 1H, H_4_), 7.91 (d, *J* = 8.7 Hz, 1H, H_5_), 4.16 (t, *J* = 6.2 Hz, 2H, NCH_2_), 3.98 (s, 3H, OCH_3_), 3.40 (t, *J* = 5*.7* Hz, 2H, OCH_2_), 3.22 (s, 3H, OCH_3_), 2.00 (dt, *J* = 6.2, 5.7 Hz, 2H, CH_2_); ^13^C-NMR (DMSO-*d*_6_) δ 160.6, 160.0, 159.0, 150.8, 148.7, 147.6, 131.6, 129.4, 126.8, 115.9, 69.1, 57.9, 54.0, 44.4, 28.2; ν_max_ 3272, 2946, 1658, 1635, 1588, 1350, 1105, 889, 831 cm^−1^; HRMS calcd for C_15_H_17_N_4_O_3_S [M + H]^+^ 333.1021 found 333.1030.

*Methyl 8-isopropyl-9-oxo-8,9-dihydrothiazolo[5,4-f]quinazoline-2-carbimidate* (**12d**): white solid (69.5 mg, 92%), mp. 234–236 °C; ^1^H-NMR (DMSO*-d*_6_) δ 9.45 (s, 1H, NH), 8.73 (s, 1H, H_7_), 8.55 (d, *J* = 9.0 Hz, 1H, H_4_), 7.93 (d, *J* = 9.0 Hz, 1H, H_5_), 5.15–5.08 (sept, *J* = 6.9 Hz, 1H, NCH), 3.99 (s, 3H, OCH_3_), 1.50 (d, *J* = 6.9 Hz, 6H, CH_3_); ^13^C-NMR (DMSO*-d*_6_) δ 160.6, 160.0, 158.7, 150.8, 147.1, 146.1, 131.9, 129.5, 126.8, 115.8, 54.0, 47.0, 21.2 (2C); ν_max_ 3315, 3061, 2977, 1673, 1657, 1583, 1347, 1072, 838, 699 cm^−1^; HRMS calcd for C_14_H_15_N_4_O_2_S [M + H]^+^ 303.0916 found 303.0919.

*Methyl 8-cyclopentyl-9-oxo-8,9-dihydrothiazolo[5,4-f]quinazoline-2-carbimidate* (**12e**): white solid (73 mg, 89%), mp. 226–228 °C; ^1^H-NMR (DMSO-*d*_6_) δ 9.45 (s, 1H, NH), 8.66 (s, 1H, H_7_), 8.55 (d, *J* = 8.7 Hz, 1H, H_4_), 7.91 (d, *J* = 8.7 Hz, 1H, H_5_), 5.13–5.05 (m, 1H, NCH), 3.98 (s, 3H, OCH_3_), 2.22–2.09 (m, 2H, CH), 2.04–1.87 (m, 4H, CH), 1.77–1.65 (m, 2H, CH); ^13^C-NMR (DMSO-*d*_6_) δ 160.7, 160.0, 159.1, 150.9, 147.1, 146.6, 131.9, 129.5, 126.8, 115.8, 56.6, 54.1, 31.0 (2C), 24.1 (2C); ν_max_ 3293, 2933, 2862, 1657, 1636, 1579, 1494, 1434, 1349, 1254, 840 cm^−1^; HRMS calcd for C_16_H_17_N_4_O_2_S [M + H]^+^ 329.1072 found 329.1077.

*Methyl 8-cyclobutyl-9-oxo-8,9-dihydrothiazolo[5,4-f]quinazoline-2-carbimidate* (**12f**): white solid (59.7 mg, 76%), mp. 260–262 °C; ^1^H-NMR (DMSO-*d*_6_) δ 9.46 (s, 1H, NH), 8.72 (s, 1H, H_7_), 8.56 (d, *J* = 8.7 Hz, 1H, H_4_), 7.93 (d, *J* = 8.7 Hz, 1H, H_5_), 5.15–5.07 (m, 1H, NCH), 3.99 (s, 3H, OCH_3_), 1.90–1.85 (m, 4H, CH), 1.23–1.18 (m, 2H, CH); ^13^C-NMR (DMSO-*d*_6_) δ 162.0 (2C), 159.8, 151.9, 147.5, 144.3, 133.2, 129.9, 126.8, 116.5, 54.5, 50.9, 29.9 (2C), 15.5; ν_max_ 3296, 2983, 2955, 1661, 1636, 1580, 1494, 1436, 1351, 1255, 1155, 841 cm^−1^; HRMS calcd for C_15_H_15_N_4_O_2_S [M + H]^+^ 315.0916 found 315.0918.

*Methyl 8-cyclohexyl-9-oxo-8,9-dihydrothiazolo[5,4-f]quinazoline-2-carbimidate* (**12g**)*:* white powder (78 mg, 92%), mp. 246–248 °C; ^1^H-NMR (DMSO-*d*_6_) δ 9.45 (s, 1H, NH), 8.73 (s, 1H, H_7_), 8.56 (d, *J* = 8.7 Hz, 1H, H_4_), 7.93 (d, *J* = 8.7 Hz, 1H, H_5_), 4.79–4.72 (m, 1H, NCH), 3.99 (s, 3H, OCH_3_), 1.97–1.38 (m, 10, CH); ^13^C-NMR (DMSO-*d*_6_) δ 162.0, 161.9, 159.5, 151.8, 147.3, 144.2, 133.4, 129.9, 126.7, 116.6, 54.5, 32.7 (2C), 26.0 (2C), 35.3; ν_max_ 3298, 2946, 2856, 1658, 1586, 1333, 1139, 1078, 834 cm^−1^; HRMS calcd for C_17_H_19_N_4_O_2_S [M + H]^+^ 343.1229 found 343.1224.

*Methyl 8-dimethylamino-9-oxo-8,9-dihydrothiazolo[5,4-f]quinazoline-2-carbimidate* (**12h**): white solid (53.1 mg, 70%), mp. 246–248 °C; ^1^H-NMR (DMSO-*d*_6_) δ 9.46 (s, 1H, NH), 8.57 (d, *J* = 9.0 Hz, 1H, H_4_), 8.52 (s, 1H, H_7_), 7.94 (d, *J* = 9.0 Hz, 1H, H_5_), 3.99 (s, 3H, OCH_3_), 3.10 (s, 6H, NCH_3_); ^13^C-NMR (CDCl_3_) δ 162.1, 162.0, 160.0, 151.9, 149.3, 147.4, 133.0, 130.0, 127.0, 118.1, 54.5, 45.0 (2C); ν_max_ 3299, 3073, 3036, 2945, 1670, 1637, 1580, 1437, 1347, 1158, 1079, 838, 808, 706 cm^−1^; HRMS calcd for C_13_H_14_N_5_O_2_S [M + H]^+^ 304.0868 found 304.0871.

*Methyl 8-[(3-dimethylamino)ethyl]-9-oxo-8,9-dihydrothiazolo[5,4-f]quinazoline-2-carbimidate* **(12i)**: white solid (66.3 mg, 80%), mp. 178–180 °C; ^1^H-NMR (DMSO-d_6_) δ 9.46 (s, 1H, NH), 8.57 (s, 1H, H_7_), 8.56 (d, *J* = 9.0 Hz, 1H, H_4_), 7.92 (d, *J* = 9.0 Hz, 1H, H_5_), 4.21 (t, *J* = 6.0 Hz, 2H, NCH_2_), 3.98 (s, 3H, OCH_3_), 2.61 (t, *J* = 6.0 Hz, 2H, NCH_2_), 2.20 (s, 6H, NCH_3_); ^13^C-NMR (DMSO-*d*_6_) δ 158.9, 150.3, 149.7, 148.7, 139.1, 131.7, 130.0, 127.9, 115.4, 113.5, 57.0, 45.2 (2C), 44.0, 30.7; ν_max_ 3201, 2981, 2774, 1650, 1587, 1442, 1341, 1066, 826 cm^−1^; HRMS calcd for C_15_H_18_N_5_O_2_S [M + H]^+^ 332.1181 found 332.1178.

*Methyl 8-(2-morpholinoethyl)-9-oxo-8,9-dihydrothiazolo[5,4-f]quinazoline-2-carbimidate* (**12j**): light yellow solid (80.3 mg, 86%), mp. 190–192 °C; ^1^H-NMR (DMSO-*d*_6_) δ 9.41 (s, 1H, NH), 8.56 (s, 1H, H_7_), 8.52 (d, *J* = 9.0 Hz, 1H, H_4_), 7.92 (d, *J* = 9.0 Hz, 1H, H_5_), 4.22 (t, *J* = 6.0 Hz, 2H, NCH_2_), 3.98 (s, 3H, OCH_3_), 3.54–3.51 (m, 4H, OCH_2_), 2.67 (t, *J* = 6.0 Hz, 2H, NCH_2_), 2.48–2.46 (m, 4H, NCH_2_); ^13^C-NMR (DMSO-*d*_6_) δ 160.6, 160.0, 159.0, 150.8, 148.9, 147.6, 131.7, 129.5, 126.8, 115.8, 115.8, 66.2 (2C), 56.2, 54.0, 53.1 (2C), 42.9; ν_max_ 3452, 3296, 2944, 2804, 1649, 1587, 1497, 1439, 1340, 1065, 1112, 1065, 825 cm^−1^; HRMS calcd for C_17_H_20_N_5_O_3_S [M + H]^+^ 374.1287 found 374.1278.

*Methyl 8-(p-tolyl)-9-oxo-8,9-dihydrothiazolo[5,4-f]quinazoline-2-carbimidate* (**12k**): white solid (59.0 mg, 68%), mp > 265 °C; ^1^H-NMR (DMSO-*d*_6_) δ 9.47 (s, 1H, NH), 8.55 (d, *J* = 8.7 Hz, 1H, H_5_), 8.57 (s, 1H, H_7_), 7.99 (d, *J* = 8.7 Hz, 1H, H_4_), 7.51 (d, *J* = 8.4 Hz, 2H, H_Ar_), 7.42 (d, *J* = 8.4 Hz, 2H, H_Ar_), 3.97 (s, 3H, OCH_3_), 2.42 (s, 3H, CH_3_); ν_max_ 3257, 3060, 1644, 1576, 1490, 1320, 1263, 1185.0, 803, 781 cm^−1^; HRMS calcd for C_18_H_15_N_4_O_2_S [M + H]^+^ 351,0916 found 351.0950.

*Methyl 8-(4-methoxyphenyl)-9-oxo-8,9-dihydrothiazolo[5,4-f]quinazoline-2-carbimidate* (**12l**): white solid (74.6 mg, 82%), mp > 265 °C; ^1^H-NMR (DMSO-*d*_6_) δ 9.47 (s, 1H, NH), 8.61 (d, *J* = 8.7 Hz, 1H, H_4_), 8.57 (s, 1H, H_7_), 7.99 (d, *J* = 8.7 Hz, 1H, H_5_), 7.55 (d, *J* = 9.0 Hz, 2H, H_Ar_), 7.15 (d, *J* = 9.0 Hz, 2H, ArH), 3.98 (s, 3H, OCH_3_), 3.86 (s, 3H, OCH_3_); ν_max_ 3307, 3061, 1671, 1582, 1516, 1339, 1261, 832 cm^−1^; HRMS calcd for C_18_H_15_N_4_O_3_S [M + H]^+^ 367.0865 found 367.0873.

*Methyl 8-(4-dimethylaminophenyl)-9-oxo-8,9-dihydrothiazolo[5,4-f]quinazoline-2-carbimidate* (**12m**): yellow solid (70.2 mg, 74%), mp > 265 °C; ^1^H-NMR (DMSO-*d*_6_) δ 9.46 (s, 1H, NH), 8.60 (d, *J* = 8.7 Hz, 1H, H_4_), 8.54 (s, 1H, H_7_), 7.98 (d, *J* = 8.7 Hz, 1H, H_5_), 7.38 (d, *J* = 9.0 Hz, 2H, H_Ar_), 6.86 (d, *J* = 9.0 Hz, 2H, H_Ar_), 3.98 (s, 3H, OCH_3_), 2.99 (s, 6H, NCH_3_); ν_max_ 3245, 2932, 2854, 1659, 1587, 1347, 1192, 836, 805 cm^−1^; HRMS calcd for C_19_H_18_N_5_O_2_S [M + H]^+^ 380.1181 found 380.1196.

*Methyl 8-methyl-9-oxo-8,9-dihydrothiazolo[5,4-f]quinazoline-2-carbimidate* (**12n**): white solid (61.7 mg, 90%), mp. 244–246 °C; ^1^H-NMR (DMSO-*d*_6_) δ 9.44 (s, 1H, NH), 8.62 (s, 1H, H_7_), 8.54 (d, *J* = 8.7 Hz, 1H, H_4_), 7.91 (d, *J* = 8.7 Hz, 1H, H_5_), 3.98 (s, 3H, OCH_3_), 3.32 (s, 3H, NCH_3_); ^13^C-NMR (DMSO-*d*_6_) δ 160.6, 160.0, 159.5, 150.8, 149.1, 147.8, 135.4, 131.6, 129.4, 126.9, 115.8, 54.0, 33.9; ν_max_ 3257, 2949, 1655, 1589, 1436, 1339, 1153, 1064, 839, 716, 498 cm^−1^; HRMS calcd for C_12_H_11_N_4_O_2_S [M + H]^+^ 275.0603 found 275.0608.

##### Benzyl 8-cyclopropyl-9-oxo-8,9-dihydrothiazolo[5,4-*f*]quinazoline-2-carbimidates (**13a**–**h**)

*Benzyl 8-cyclopropyl-9-oxo-8,9-dihydrothiazolo[5,4-f]quinazoline-2-carbimidate* (**13a**): white solid (48 mg, 85%), mp. 214–216 °C; ^1^H-NMR (CDCl_3_) δ 9.60 (s, 1H, NH), 8.56 (d, *J* = 9.0 Hz, 1H, H_4_), 8.53 (s, 1H, H_7_), 7.92 (d, *J* = 9.0 Hz, 1H, H_5_), 7.57–7.40 (m, 5H, Ph), 5.48 (s, 2H, CH_2_Ph), 3.30–3.15 (m, 1H, CH), 1.11–1.06 (m, 4H, CH); ^13^C-NMR (CDCl_3_) δ 162.1, 161.2, 161.1, 152.0, 147.4, 147.1, 136.0, 133.3, 130.0, 128.7 (2C), 128.3, 127.9 (2C), 126.9, 116.5, 68.8, 29.8, 6.7 (2C); ν_max_ 3286, 3024, 1660, 1636, 1586, 1491, 1335, 1155, 1084, 833, 734 cm^−1^; HRMS calcd for C_20_H_17_N_4_O_2_S [M + H]^+^ 377.1072 found 377.1082.

*Benzyl 8-(2-methoxyethyl)-9-oxo-8,9-dihydrothiazolo[5,4-f]quinazoline-2-carbimidate* (**13b**): white solid (57.1 mg, 58%), mp. 160–162°C; ^1^H-NMR (DMSO-*d*_6_) δ 9.61 (s, 1H, NH), 8.58 (d, *J* = 9.0 Hz, 1H, H_4_), 8.54 (s, 1H, H_7_), 7.93 (d, *J* = 9.0 Hz, 1H, H_5_), 7.56–7.38 (m, 5H, Ph), 5.47 (s, 2H, CH_2_Ph), 4.29 (t, *J* = 5.1 Hz, 2H, OCH_2_), 3.67 (t, *J* = 5.1 Hz, 2H, NCH_2_), 3.26 (s, 3H, OCH_3_); ^13^C-NMR (DMSO-*d*_6_) δ 160.7, 159.1, 159.0, 151.0, 149.0, 147.6, 136.3 ,131.7, 129.7, 128.5 (2C), 128.0, 127.8 (2C), 126.9, 115.9, 69.1, 67.8, 58.1, 45.9; ν_max_ 3456, 3396, 3275, 2925, 1656, 1589, 1334, 1105, 1005, 837 cm^−1^; HRMS calcd for C_20_H_19_N_4_O_3_S [M + H]^+^ 395.1178 found 395.1177.

*Benzyl 8-(3-methoxypropyl)-9-oxo-8,9-dihydrothiazolo[5,4-f]quinazoline-2-carbimidate* (**13c**): white solid (56.2 mg, 55%), mp. 168–170 °C; ^1^H-NMR (DMSO-*d*_6_) δ 9.60 (s, 1H, NH), 8.59 (s, 1H, H_7_), 8.57 (d, *J* = 9.0 Hz, 1H, H_4_), 7.93 (d, *J* = 9.0 Hz, 1H, H_5_), 7.56–7.39 (m, 5H, Ph), 5.47 (s, 1H, CH_2_Ph), 4.16 (t, *J* = 6.9 Hz, 2H, NCH_2_), 3.40 (t, *J* = 6.0 Hz, 2H, OCH_2_), 3.21 (s, 3H, OCH_3_), 2.00 (dt, *J* = 6.9, 6.0 Hz, 2H, CH_2_); ^13^C-NMR (DMSO*-d*_6_) δ 160.7, 159.2, 159.1, 150.9, 148.7, 147.7, 136.3 ,131.7, 129.5, 128.4 (2C), 128.0, 127.8 (2C), 126.9, 116.0, 69.1, 67.8, 57.9, 44.4, 28.2; ν_max_ 3272, 3078, 2923, 2865, 1666, 1647, 1584, 1453, 1317, 1113, 1050, 890, 837, 749 cm^−1^; HRMS calcd for C_21_H_21_N_4_O_3_S [M + H]^+^ 409.1334 found 409.1333.

*Benzyl 8-isopropyl-9-oxo-8,9-dihydro*[1,3]*thiazolo[5,4-f]quinazoline-2-carbimidate* (**13d**): white solid (61 mg, 65%), mp. 212–214 °C; ^1^H-NMR (DMSO-*d*_6_) δ 9.60 (s, 1H, NH), 8.72 (s, 1H, H_7_), 8.56 (d, *J* = 8.7 Hz, 1H, H_4_), 7.93 (d, *J* = 8.7 Hz, 1H, H_5_), 7.56–7.38 (m, 5H, Ph), 5.47 (s, 2H, CH_2_Ph), 5.02–5.11 (sept, *J* = 6.9 Hz, 1H, NCH), 1.50 (d, *J* = 6.9 Hz, 6H, CH_3_). Other data supporting its chemical structure are reported in [6].

*Benzyl 8-cyclopentyl-9-oxo-8,9-dihydrothiazolo[5,4-f]quinazoline-2-carbimidate* (**13e**): white solid (91 mg, 90%), mp. 214–216 °C; ^1^H-NMR (DMSO-*d*_6_) δ 9.58 (s, 1H, NH), 8.65 (s, 1H, H_7_), 8.55 (d, *J* = 8.7 Hz, 1H, H_4_), 7.92 (d, *J* = 8.7 Hz, 1H, H_5_), 7.57–7.39 (m, 5H, Ph), 5.47 (s, 2H, CH_2_Ph), 5.13–5.02 (m, 1H, NCH), 2.21–2.09 (m, 2H, CH_2_), 2.00–1.87 (m, 4H, 2 × CH_2_), 1.79–1.60 (m, 2H, CH_2_); ^13^C-NMR (DMSO-*d*_6_) δ 161.9, 161.3, 160.0, 151.9, 147.4, 144.7, 136.0, 133.4, 129.9, 128.7 (2C), 128.3, 128.0 (2C), 126.8, 116.6, 68.8, 57.0, 32.2 (2C), 24.6 (2C); ν_max_ 3300, 2926, 2869, 1671, 1660, 1632, 1583, 1491, 1335, 1318, 1257, 1144, 830, 745 cm^−1^; HRMS calcd for C_22_H_21_N_4_O_2_S [M + H]^+^ 405.1385 found 405.1394.

*Benzyl 8-cyclobutyl-9-oxo-8,9-dihydrothiazolo[5,4-f]quinazoline-2-carbimidate* (**13f**): white solid (84 mg, 86%), mp. 234–236 °C; ^1^H-NMR (CDCl_3_) δ 9.11 (s, 1H, NH), 8.44 (d, *J* = 8.7 Hz, 1H, H_4_), 8.32 (s, 1H, H_7_), 7.89 (d, *J* = 8.7 Hz, 1H, H_5_), 7.53–7.36 (m, 5H, Ph), 5.50 (s, 2H, CH_2_Ph), 5.16–5.04 (m, 1H, NCH), 2.63 (m, 2H, 2 × CH), 2.52–2.43 (m, 2H,2 × CH), 2.02–2.00 (m, 2H, 2 x CH); ^13^C-NMR (CDCl_3_) δ 161.9, 161.3, 159.9, 152.0, 147.6, 144.3, 136.0, 133.3, 130.0, 128.7 (2C), 128.3, 128.0 (2C), 126.9, 116.5, 68.9, 51.2, 29.8 (2C), 15.6; ν_max_ 3301, 3055, 2983, 2955, 2921, 1659, 1640, 1581, 1340, 1159, 830, 733 cm^−1^; HRMS calcd for C_21_H_19_N_4_O_2_S [M + H]^+^ 391.1229 found 391.1216.

*Benzyl 8-cyclohexyl-9-oxo-8,9-dihydrothiazolo[5,4-f]quinazoline-2-carbimidate* (**13g**): white solid (59 mg, 94%), mp. 212–214 °C; ^1^H-NMR (CDCl_3_) δ 9.11 (s, 1H, NH), 8.42 (d, *J* = 8.7 Hz, 1H, H_4_), 8.27 (s, 1H, H_7_), 7.85 (d, *J* = 8.7 Hz, 1H, H_5_), 7.52–7.34 (m, 5H, Ph), 5.50 (s, 2H, CH_2_Ph), 4.89–4.81 (m, 1H, NCH), 2.08-1.25 (m, 10H, 5 × CH_2_); ^13^C-NMR (CDCl_3_) δ 162.0, 161.4, 159.7, 152.0, 147.4, 144.3, 136.2, 133.6, 130.0, 128.8, 128.3, 127.9, 126.9, 68.9, 54.9, 32.8, 26.1, 25.4; ν_max_ 3301, 2931, 2861, 1656, 1627, 1583, 1490, 1334, 1146, 1078, 833, 744, 700 cm^−1^; HRMS calcd for C_23_H_23_N_4_O_2_S [M + H]^+^ 419.1542 found 419.1546.

*Benzyl 8-dimethylamino-9-oxo-8,9-dihydrothiazolo[5,4-f]quinazoline-2-carbimidate* (**13h**): white solid (45 mg, 80%), mp. 190–192 °C; ^1^H-NMR (DMSO-*d*_6_) δ 9.61 (s, 1H, NH), 8.58 (d, *J =* 9.0 Hz, 1H, H_4_), 8.52 (s, 1H, H_7_), 7.94 (d, *J* = 9.0 Hz, 1H, H_5_), 7.57–7.37 (m, 5H, Ph), 5.48 (s, 2H, CH_2_Ph), 3.09 (s, 6H, 2 × CH_3_); ^13^C-NMR (DMSO-*d*_6_) δ 161.1, 159.1, 151.0, 150.0, 147.0, 138.3, 129.7, 128.5 (2C), 128.1, 127.9 (2C), 117.6, 67.9, 44.2 (2C); ν_max_ 3291, 3038, 2922, 1688, 1642, 1578, 1493, 1444, 1330, 1295, 1164, 1072, 916, 827, 752, 699 cm^−1^; HRMS calcd for C_19_H_18_N_5_O_2_S [M + H]^+^ 380.1181 found 380.1183.

##### Ethyl 9-oxo-8,9-dihydrothiazolo[5,4-*f*]quinazoline-2-carbimidates (**14a**–**e**)

*Ethyl 8-cyclopropyl-9-oxo-8,9-dihydrothiazolo[5,4-f]quinazoline-2-carbimidate* (**14a**): white solid (73.0 mg, 93%), mp. 248–250 °C; ^1^H-NMR (CDCl_3_) δ 8.94 (s, 1H, NH), 8.42 (d, *J* = 9.0. Hz, 1H, H_4_), 8.27 (s, 1H, H_7_), 7.86 (d, *J* = 9.0 Hz, 1H, H_5_), 4.49 (q, *J* = 7.2 Hz, 2H, OCH_2_), 3.41–3.33 (m, 1H, NCH), 1.48 (t, *J* = 7.2 Hz, 3H, CH_3_), 1.33–1.24 (m, 2H, CH), 1.06–1.03 (m, 2H, CH); ^13^C-NMR (CDCl_3_) δ 162.6, 161.4, 161.1, 152.0, 147.3, 147.1, 133.2, 130.0, 126.8, 116.5, 63.2, 29.7, 14.3, 6.7 (2C); ν_max_ 3279, 2982, 1666, 1633, 1584, 1497, 1345, 1274, 1076, 866, 845 cm^−1^; HRMS calcd for C_15_H_15_N_4_O_2_S [M + H]^+^ 315.0916 found 315.0901.

*Ethyl 8-(2-methoxyethyl)-9-oxo-8,9-dihydrothiazolo[5,4-f]quinazoline-2-carboximidoate* (**14b**): white solid (63.2 mg, 76%), mp. 166–168 °C, ^1^H-NMR (DMSO-*d*_6_) δ 9.36 (br s, 1H, NH), 8.54 (d, *J* = 9.0 Hz, 1H, H_4_), 8.53 (s, 1H, H_7_), 7.90 (d, *J* = 9.0 Hz, 1H, H_5_), 4.42 (q, *J* = 7.2 Hz, 2H, OCH_2_), 4.30 (t, *J* = 5.1 Hz, 2H, OCH_2_), 3.68 (t, *J* = 5.1 Hz, 2H, NCH_2_), 3.27 (s, 3H, OCH_3_), 1.41 (t, *J* = 7.2 Hz, 3H, CH_3_); ν_max_ 3308, 3160, 2985, 1663, 1589, 1347, 1112, 1005, 837 cm^−1^; HRMS calcd for C_15_H_17_N_4_O_3_S [M + H]^+^ 333.1021 found 333.1023.

*Ethyl 8-(3-methoxypropyl)-9-oxo-8,9-dihydrothiazolo[5,4-f]quinazoline-2-carboximidoate* (**14c**): white solid (62 mg, 72%), mp. 172–174 °C; ^1^H-NMR (DMSO-*d_6_*) δ 9.37 (s, 1H, NH), 8.59 (s, 1H, H_7_), 8.55 (d, *J* = 8.9 Hz, 1H, H_4_), 7.91 (d, *J* = 8.9 Hz, 1H, H_5_), 4.42 (q, *J* = 7.2 Hz, 1H, CH_2_), 4.17 (t, *J* = 6.9 Hz, 2H, OCH_2_), 3.40 (t, *J* = 6.0 Hz, 2H, NCH_2_), 3.32 (s, 3H, OCH_3_), 2.00 (dt, *J* = 6.9, 6.0 Hz, 2H, CH_2_), 1.41 (t, *J* = 7.2 Hz, 3H, CH_3_); ^13^C-NMR (DMSO-*d*_6_) δ 160.9, 159.4, 159.1, 150.9, 148.7, 147.6, 131.7, 129.5, 126.8, 116.0, 69.1, 62.5, 57.9, 44.4, 28.2, 14.0; ν_max_ 3280, 2973, 2867, 1660, 1640, 1581, 1327, 1107, 893, 827 cm^−1^; HRMS calcd for C_16_H_19_N_4_O_3_S [M + H]^+^ 347.1173, found 347.1178.

*Ethyl 8-isopropyl-9-oxo-8,9-dihydrothiazolo[5,4-f]quinazoline-2-carboximidoate* (**14d**): white solid (63.3.mg, 80%), mp. 196–198 °C; ^1^H-NMR (DMSO*-d*_6_) *δ* 9.37 (s, 1H, NH), 8.72 (s, 1H, H_7_), 8.55 (d, 8.7 Hz, 1H, H_4_), 7.92 (d, 8.7 Hz, 1H, H_5_), 5.13–5.04 (m, 1H, NCH), 4.42 (q, *J* = 6.9 Hz, 2H, OCH_2_), 1.50 (d, *J* = 6.9 Hz, 6H, 2x CH_3_), 1.42 (t, 7.2 Hz, 1H, CH_3_). Other data supporting its chemical structure are reported in [6].

*Ethyl 8-cyclopentyl-9-oxo-8,9-dihydrothiazolo[5,4-f]quinazoline-2-carbimidat*e (**14e**): white solid (79 mg, 92%), mp. 218-220 °C; ^1^H-NMR (CDCl_3_) δ 8.94 (s, 1H, NH), 8.41 (d, *J* = 9.0 Hz, 1H, H_4_), 8.27 (s, 1H, H_7_), 7.86 (d, *J* = 9.0 Hz, 1H, H_5_), 5.22 (m, 1H, NCH), 4.49 (q, *J* = 6.9 Hz, 2H, OCH_2_), 2.27 (m, 2H, CH), 1.95 (m, 6H, CH), 1.48 (t, *J* = 6.9 Hz, 3H, CH_3_); ^13^C-NMR (CDCl_3_) δ 162.3, 161.4, 159.8, 151.8, 147.2, 144.6, 133.2, 129.8, 126.6, 116.5, 63.1, 56.9, 32.1 (2C), 24.6 (2C), 14.2; ν_max_ 3274, 3063, 2922, 1647, 1587, 1495, 1323, 21143, 1118, 1055, 836 cm^−1^; HRMS calcd for C_17_H_19_N_4_O_2_S [M + H]^+^ 343.1229 found 343.1232.

##### Isopropyl 9-oxo-8,9-dihydrothiazolo[5,4-f]quinazoline-2-carbimidates **15a**–**c**

*Isopropyl 8-cyclopropyl-9-oxo-8,9-dihydrothiazolo[5,4-f]quinazoline-2-carbimidate* (**15a**): white solid (72.2 mg, 88%), mp. 228–230 °C; ^1^H-NMR (DMSO-*d*_6_) δ 9.34 (s, 1H, NH), 8.54 (d, *J* = 8.7 Hz, 1H, H_4_), 8.53 (s, 1H, H_7_), 7.91 (d, *J =* 8.7 Hz, 1H, H_5_), 5.29 (sept, *J* = 6.3 Hz, 1H, NCH), 3.30–3.25 (m, 1H, CH), 1.42 (d, *J* = 6.3 Hz, 6H, CH_3_), 1.12–1.06 (m, 4H, CH); ^13^C-NMR (CDCl_3_) δ 163.2, 161.1, 160.7, 152.0, 147.2, 147.1, 133.2, 130.0, 126.7, 116.5, 70.4, 29.8, 21.8, 6.7 (2C) ; ν_max_ 3275, 2971, 1668, 1641, 1586, 1494, 1455, 1352, 1313, 1145, 1109, 1054, 893, 823 cm^−1^; HRMS calcd for C_16_H_17_N_4_O_2_S [M + H]^+^ 329.1072 found 329.1065.

*Isopropyl 8-(2-methoxyethyl)-9-oxo-8,9-dihydro*[1,3]*thiazolo[5,4-f]quinazoline-2-carboximidate* (**15b**): white solid (56.3 mg, 65%), mp. 174–176 °C; ^1^H-NMR (DMSO-*d*_6_) δ 9.34 (br s, 1H, NH), 8.56 (d, *J* = 9.0 Hz, 1H, H_4_), 8.54 (s, 1H, H_7_), 7.92 (d, *J* = 9 .0 Hz, 1H, H_5_), 5.29 (sept, *J* = 7.0 Hz, 1H, OCH), 4.30 (t, *J* = 5.1 Hz, 2H, OCH_2_), 3.68 (t, *J* = 5.1 Hz, 2H, NCH_2_), 3.27 (s, 3H, OCH_3_), 1.41 (d, *J* = 7.0 Hz, 6H, 2x CH_3_); ^13^C-NMR (DMSO-*d*_6_) *δ* 161.5, 159.0, 158.8, 151.0, 148.9, 147.6, 131.8, 129.6, 126.8, 115.9, 69.5, 68.9, 58.1, 45.9, 21.4 (2C); ν_max_ 3278, 2981, 2928, 1658, 1641, 1588, 1350, 1140, 1111, 1052, 889, 837 cm^−1^; HRMS calcd for C_16_H_19_N_4_O_3_S [M + H]^+^ 347.1178 found 347.1170.

*Isopropyl 8-(3-methoxypropyl)-9-oxo-8,9-dihydro*[1,3]*thiazolo[5,4-f]quinazoline-2-carboximidate* (**15c**): white solid (81 mg, 90%), mp. 162–164 °C; ^1^H-NMR (DMSO-*d*_6_) δ 9.34 (s, 1H, NH), 8.59 (s, 1H, H_7_), 8.55 (d, *J* = 9.0 Hz, 1H, H_4_), 7.92 (d, *J* = 9.0 Hz, 1H, H_5_), 5.28 (sept, *J* = 6.0 Hz, 1H, CH), 4.17 (t, *J* = 6.9 Hz, 2H, OCH_2_), 3.40 (t, *J* = 6.0 Hz, 2H, NCH_2_), 3.28 (s, 3H, OCH_3_), 2.00 (dt, *J* = 6.9, 6.0 Hz, 2H, CH_2_), 1.40 (d, *J* = 6.0 Hz, 6H, CH_3_), ^13^C-NMR (DMSO-*d_6_*) δ 161.4, 159.1, 158.9, 150.9, 148.7, 147.6, 131.7, 129.5, 126.8, 115.9, 69.5, 69.1, 57.9, 44.4, 28.2, 21.4 (2C); ν_max_ 3275, 2993, 2939, 1663, 1582, 1340, 1112, 1058, 830 cm^−1^; HRMS calcd for C_17_H_21_N_4_O_3_S [M + H]^+^ 361.1331 found. 361.1334.

### 3.3. In Vitro Kinase Preparation and Assays [25]

#### 3.3.1. Buffers

*Buffer A*: MgCl_2_ (10 mM), 1 mM ethylene glycol-bis(2-aminoethylether)-*N,N,N’,N’*- tetraacetic acid (EGTA), 1 mM dithiothreitol (DTT), 25 mM Tris-HCl pH 7.5, 50 μg heparin/mL. *Buffer B*: β-Glycerophosphate (60 mM), 30 mM *p-*nitrophenylphosphate, 25 mM 3-(*N*-morpholino) propanesulfonic acid (Mops) (pH 7.2), 5 mM EGTA, 15 mM MgCl_2_, 1 mM DTT, 0.1 mM sodium vanadate.

#### 3.3.2. Kinase Preparations and Assays

Kinase activities were assayed in triplicates in buffer A or B, for 30 min. at 30 °C, at a final adenosine triphosphate (ATP) concentration of 15 μM. Blank values were substracted and activities expressed in % of the maximal activity, *i.e.*, in the absence of inhibitors. Controls were performed with appropriate dilutions of dimethylsulfoxide (DMSO). IC_50_ values were calculated from dose-response curves established by Sigma-Plot graphs. The GSK-3, CK1, DYRK1A and CLK1 peptide substrates were obtained from Proteogenix (Oberhausbergen, France).

*CDK5/p25.* (Human, recombinant) was prepared as previously described [21]. Its kinase activity was assayed in buffer A, with 1 mg of histone H1/mL, in the presence of 15 μM [γ-^33^P] ATP (3000 Ci/mmol; 10 mCi/mL) in a final volume of 30 μL. After 30 min incubation at 30 °C, 25 μL aliquots of supernatant were spotted onto sheets of P81 phosphocellulose paper (Whatman) and 20 s later, the filters were washed eight times (for at least 5 min each time) in a solution of 10 mL phosphoric acid/L of water. The wet filters were counted in the presence of 1 mL ACS (Amersham, UK) scintillation fluid.

*GSK-3α/β.* (Porcine brain, native) was assayed, as described for CDK5/p25 but in buffer A and using a GSK-3 specific substrate (GS-1: YRRAAVPPSPSLSRHSSPHQpSEDEEE) (pS stands for phosphorylated serine) [36].

*CK1δ/ε.* (Porcine brain, native) was assayed as described for CDK5/p25 but using the CK1-specific peptide substrate RRKHAAIGpSAYSITA [37].

*DYRK1A.* (Rat, recombinant, expressed in E. coli as a glutathione transferase (GST) fusion protein) was purified by affinity chromatography on glutathione-agarose and assayed, as described for CDK5/p25 using Woodtide (KKISGRLSPIMTEQ) (1.5μg/assay) as a substrate.

*CLK1.* (Human, recombinant, expressed in E. coli as GST fusion protein) was assayed in buffer A (+0.15 mg BSA/mL) with RS peptide (GRSRSRSRSRSR) (1 μg/assay).

## 4. Conclusions

A library of thirty-three novel thiazolo[5,4-*f*]quinazoline derivatives **11a**–**c**, **12a**–**n**, **13a**–**h**, **14a**–**e**, **15a**–**c** has been rapidly prepared, using microwave-assisted technology when efficient heating was needed. In order to have an efficient route to these variously 8-substituted thiazolo[5,4-*f*]quinazolin-9(8*H*)-ones, a rational multistep synthesis of methyl 6-amino-2-cyano- benzo[*d*]thiazole-7-carboxylate (**1**) was developed and optimized to the multigram scale, affording large quantities of a versatile and efficient precursor for the various target molecules in this study. The inhibitory potency of the final products against five kinases involved in AD was evaluated. Our study demonstrates that some molecules of the **12** and **13** series described in this paper are particularly promising for the development of new multi-target inhibitors of kinases. The most active compounds showed nanomolar IC_50_ values for CLK1, DYRK1A and GSK-3α/β over the other tested enzymes. Although affinities of these compounds for these three kinases are not negligible, optimization is still needed and this paper allows us to consider further SAR studies for the design of more efficient inhibitors of these targeted kinases.

## Figures and Tables

**Table 1:** Chemical structures and yields obtained for the synthesis of series **11a**–**n** ^a^.

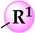	Compound	Yield ^b^ (%)	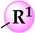	Compound	Yield ^b^ (%)
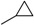	**11a**	78	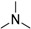	**11h**	84
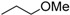	**11b**	82	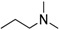	**11i**	77
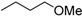	**11c**	80	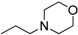	**11j**	78
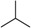	**11d**	58	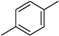	**11k**	85
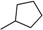	**11e**	70	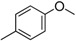	**11l**	77
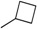	**11f**	73	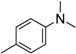	**11m**	81
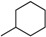	**11g**	69	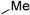	**11n**	84

^a^ Reaction time is 10 min, except for **11k**, **11l** and **11m** (6 min). ^b^ Isolated yield.

**Table 2:** Synthesis of carbimidates **12**, **13**, **14** and **15** from **11** series.

Product	Yield ^a^ (%)	Product	Yield ^a^ (%)	Product	Yield ^a^ (%)	Product	Yield ^a^ (%)
**12a**	91	**12h**	70	**13a**	87	**14a**	93
**12b**	71	**12i**	80	**13b**	58	**14b**	76
**12c**	76	**12j**	86	**13c**	55	**14c**	72
**12d**	92	**12k**	68	**13d**	65	**14d**	80
**12e**	89	**12l**	74	**13e**	90	**14e**	92
**12f**	76	**12m**	82	**13f**	86	**15a**	88
**12g**	92	**12n**	90	**13g**	94	**15b**	65
-	-	-	-	**13h**	62	**15c**	90

^a^ Isolated yield.

**Table 3:** Kinase inhibitory activity ^a,b^ of the thiazolo[5,4-*f*]quinazoline series **11a**–**c**, **12a**–**n**, **13a**–**h**, **14a**–**e**, **15a**–**c**.

Compounds	CDK5/p25	CK1δ/ε	CLK1	DYRK1A	GSK-3α/β
**11a**	>10	>10	1.1	3.3	3.3
**11b**	>10	>10	4.1	7.8	≥10
**11c**	>10	>10	1.3	7.7	1.2
**12a**	3.9	>10	**0.031**	**0.091**	**0.082**
**12b**	>10	>10	0.51	0.39	1.2
**12c**	>10	>10	0.21	0.32	0.12
**12d**	3.0	5.4	0.16	0.14	**0.041**
**12e**	4.9	>10	0.29	**0.072**	**0.028**
**12f**	>10	>10	**0.091**	0.13	0.16
**12g**	1.8	>10	0.38	0.17	**0.030**
**12h**	>10	>10	0.99	0.5	0.3
**12i**	>10	>10	1.8	1.0	1.8
**12j**	>10	>10	0.69	0.2	1.2
**12k**	>10	>10	0.88	0.38	0.51
**12l**	>10	>10	7.9	3.0	1.20
**12m**	>10	>10	>10	>10	≥10
**12n**	>10	>10	0.080	0.18	1.7
**13a**	>10	>10	**0.06**	**0.06**	0.1
**13b**	>10	>10	0.59	0.29	0.69
**13c**	>10	>10	0.48	0.16	0.11
**13d**	>10	>10	0.27	0.13	**0.057**
**13e**	>10	>10	0.32	**0.062**	**0.028**
**13f**	>10	>10	**0.071**	**0.059**	**0.071**
**13g**	>10	>10	1.30	0.28	**0.083**
**13h**	>10	>10	1.40	0.21	0.15
**14a**	>10	>10	0.1	0.21	0.2
**14b**	>10	>10	1.9	0.82	1.2
**14c**	>10	>10	0.49	0.37	0.17
**14d**	>10	>10	0.34	0.37	0.17
**14e**	>10	>10	0.78	0.23	**0.029**
**15a**	>10	>10	**0.043**	0.42	0.42
**15b**	>10	>10	2.1	3.9	2.5
**15c**	>10	>10	1.2	0.95	0.27
Harmine	>10	1.5	0.026	0.029	>10

^a^ IC_50_ values are reported in μM. The most significant results are presented in bold; ^b^ Kinases activities were assayed in triplicate. Typically, the standard deviation of single data points was <10%.

## Supplementary Materials

Supplementary materials can be accessed at: http://www.mdpi.com/1420-3049/21/5/578/s1.

## Abbreviations

The following abbreviations are used in this manuscript:

### Abbreviations

ATP: Adenosine triphosphate
CMGC group: Group of kinases including Cyclin-dependent kinases (CDKs), Mitogen-activated protein kinases (MAP kinases), Glycogen synthase kinases (GSK) and Cyclin dependent kinases (CDK-like kinases)
DMF: Dimethylformamide
MTDL: Multi-target-directed ligand
NBS: *N*-bromosuccinimide
SAR: Structure Activity Relationship
