# Supplementary material for: Synthesis of Thiazolo[5,4-f]quinazolin-9(8H)-ones as Multi-Target Directed Ligands of Ser/Thr Kinases
Source: Molecules. 2016 Apr 30;21(5):578. doi: 10.3390/molecules21050578 (PMC6273584; doi:10.3390/molecules21050578)
Supplement: Supplementary file 1 [file molecules-21-00578-s001.pdf]

Supplementary Materials: Synthesis of Thiazolo[5,4-f]quinazolin-9(8*H*)-ones as Multi-Target Directed Ligands of Ser/Thr Kinases

Damien Hédou, Julien Godeau, Nadège Loaëc, Laurent Meijer, Corinne Fruit and Thierry Besson

^{1}H- and ^{13}C-NMR spectra of compounds 10, 11a–n, 12a–n, 13a–g, 14a–e and 15a–c

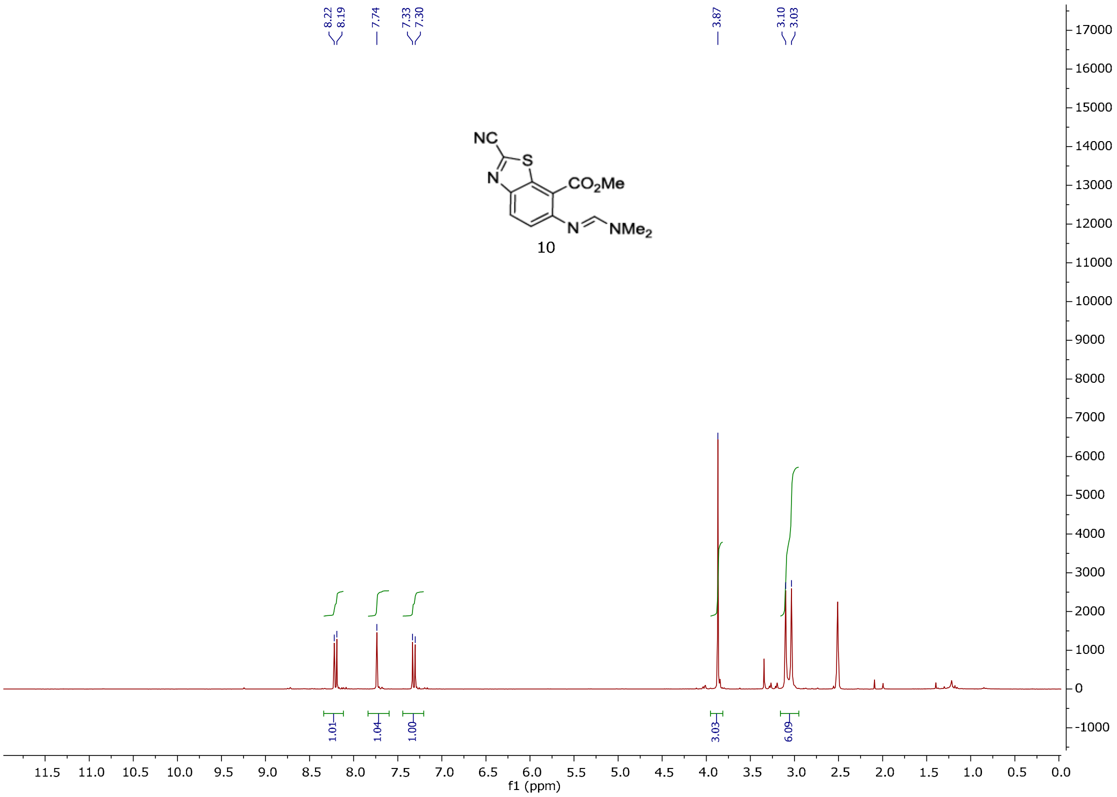

Figure S1. ^{1}H-NMR Compound **10**.

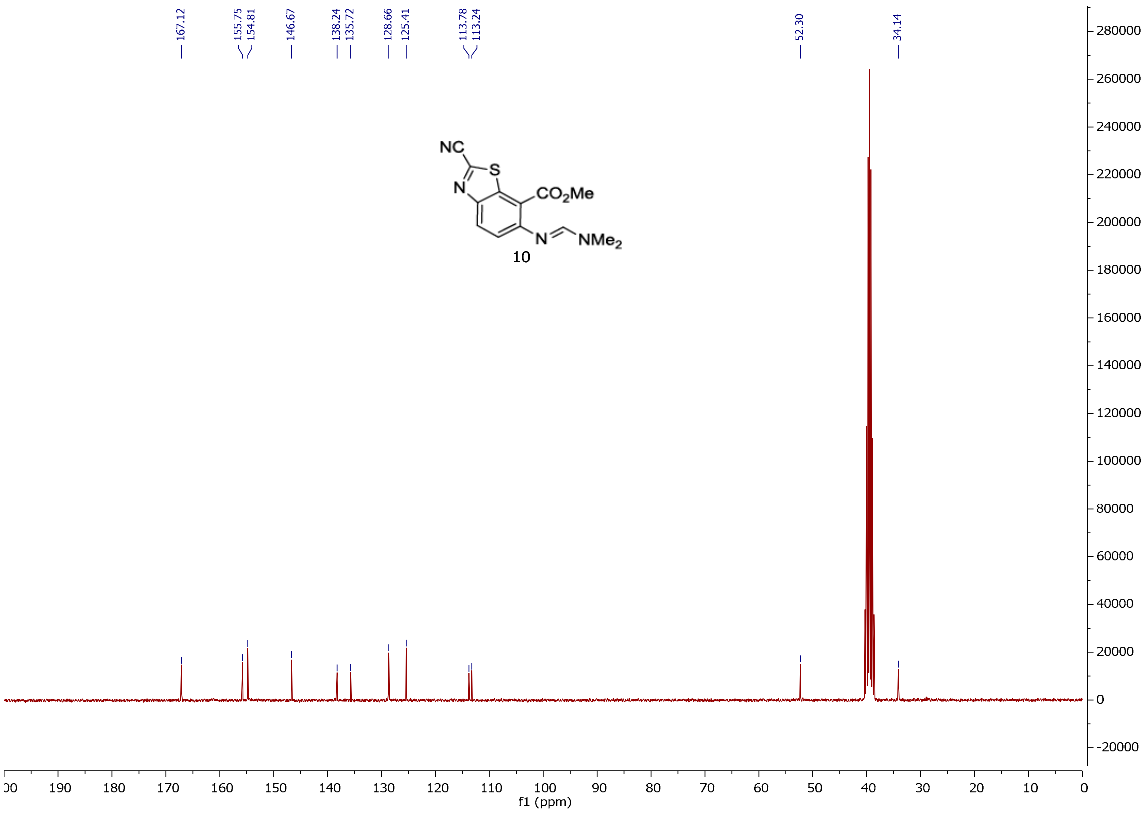

Figure S2. ^{13}C-NMR Compound **10.**

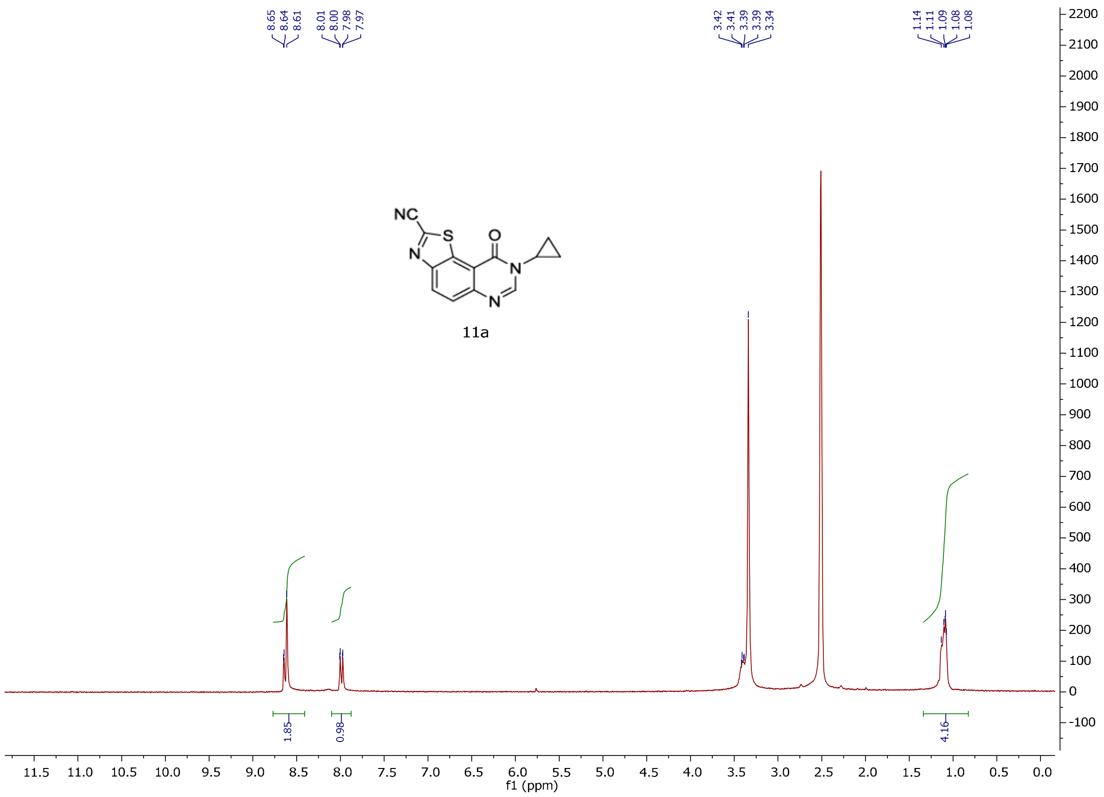

Figure S3. ^{1}H-NMR Compound **11a**.

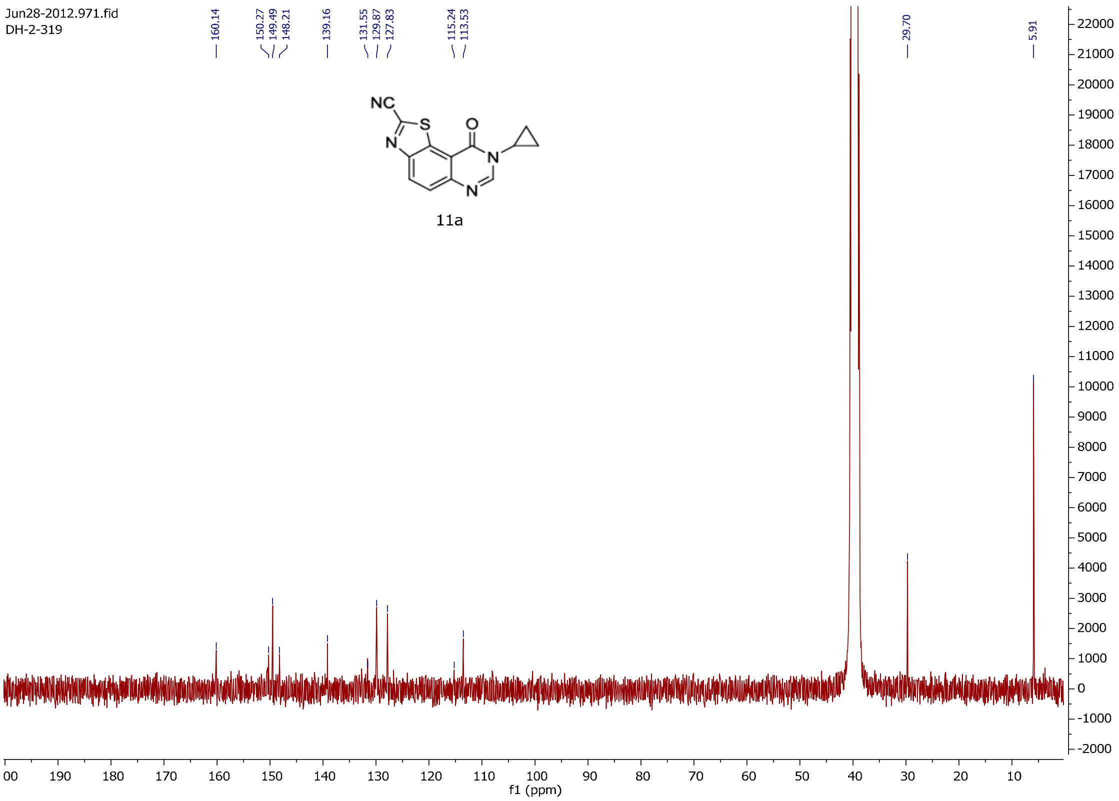

Figure S4. ^{13}C-NMR Compound **11a.**

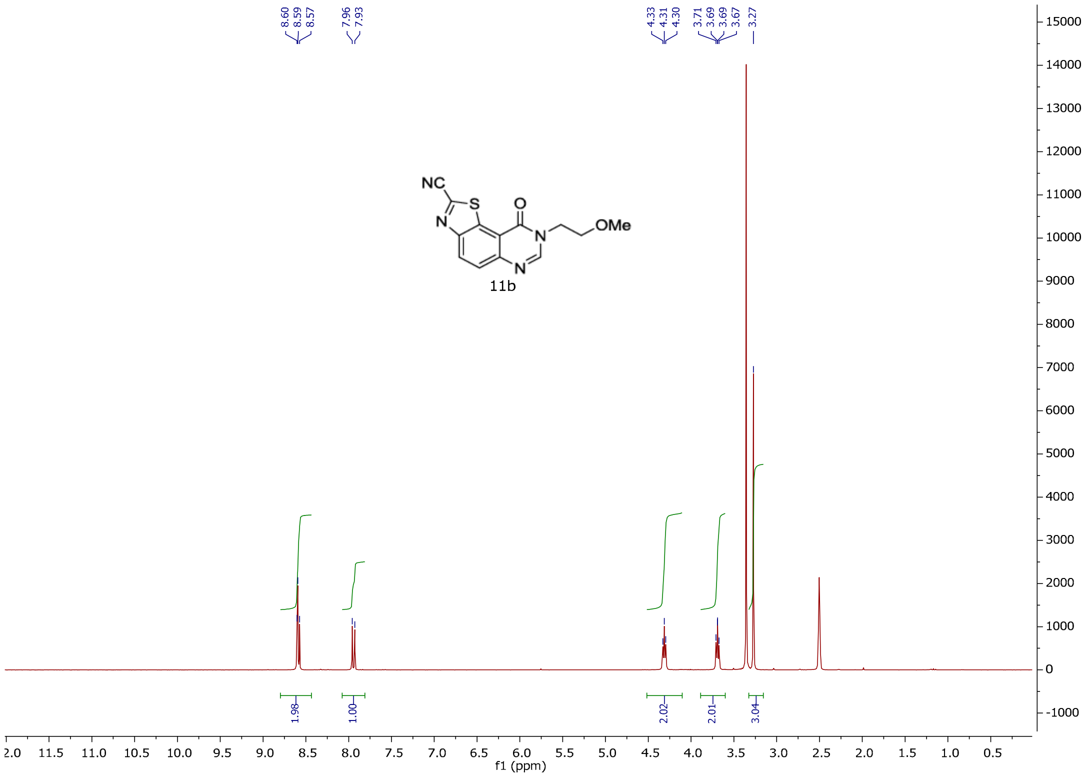

Figure S5. ^{1}H-NMR Compound **11b.**

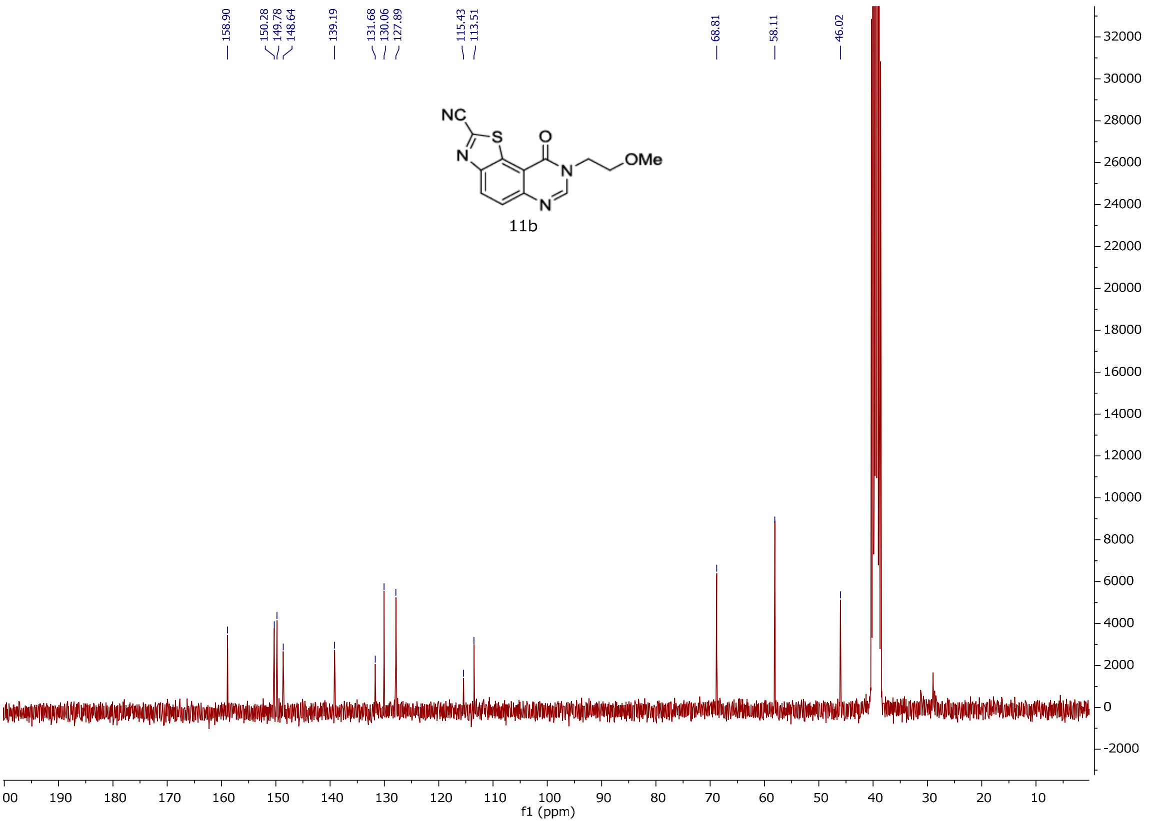

Figure S6. ^{13}C-NMR Compound **11b.**

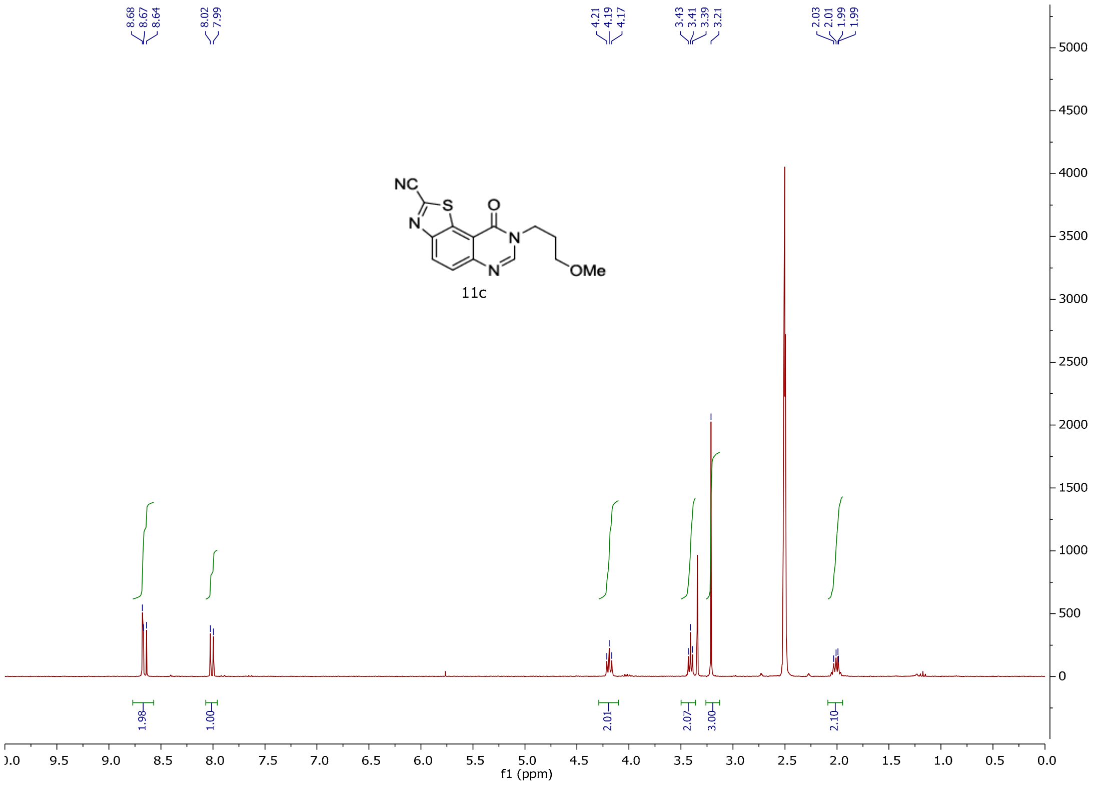

Figure S7. ^{1}H-NMR Compound **11c.**

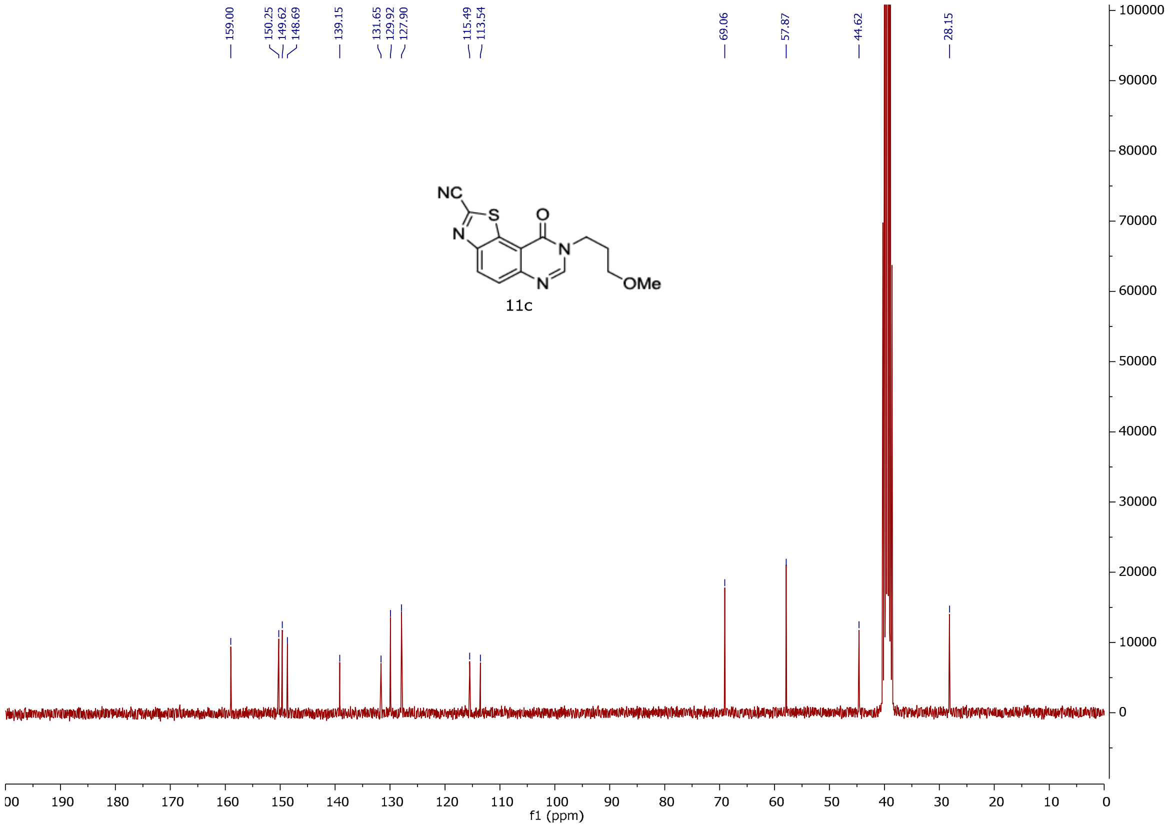

Figure S8. ^{13}C-NMR Compound **11c.**

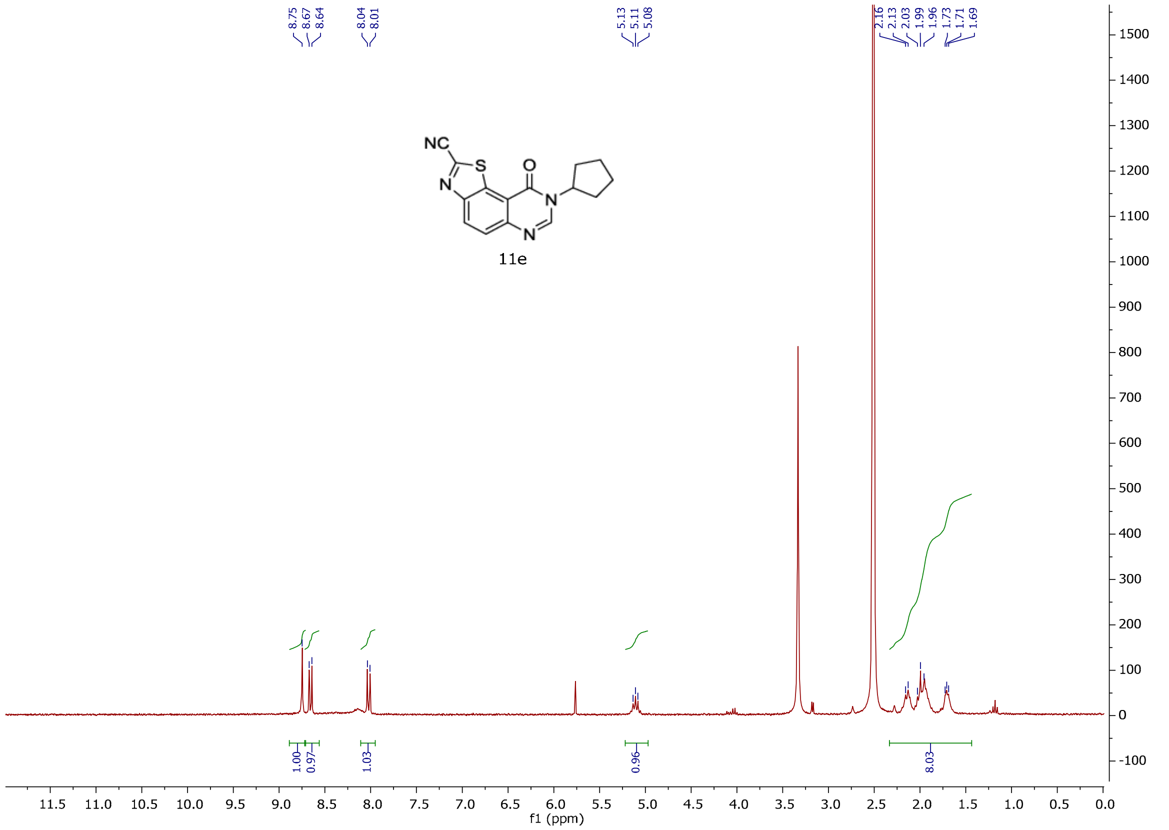

Figure S9. ^{1}H-NMR Compound **11e.**

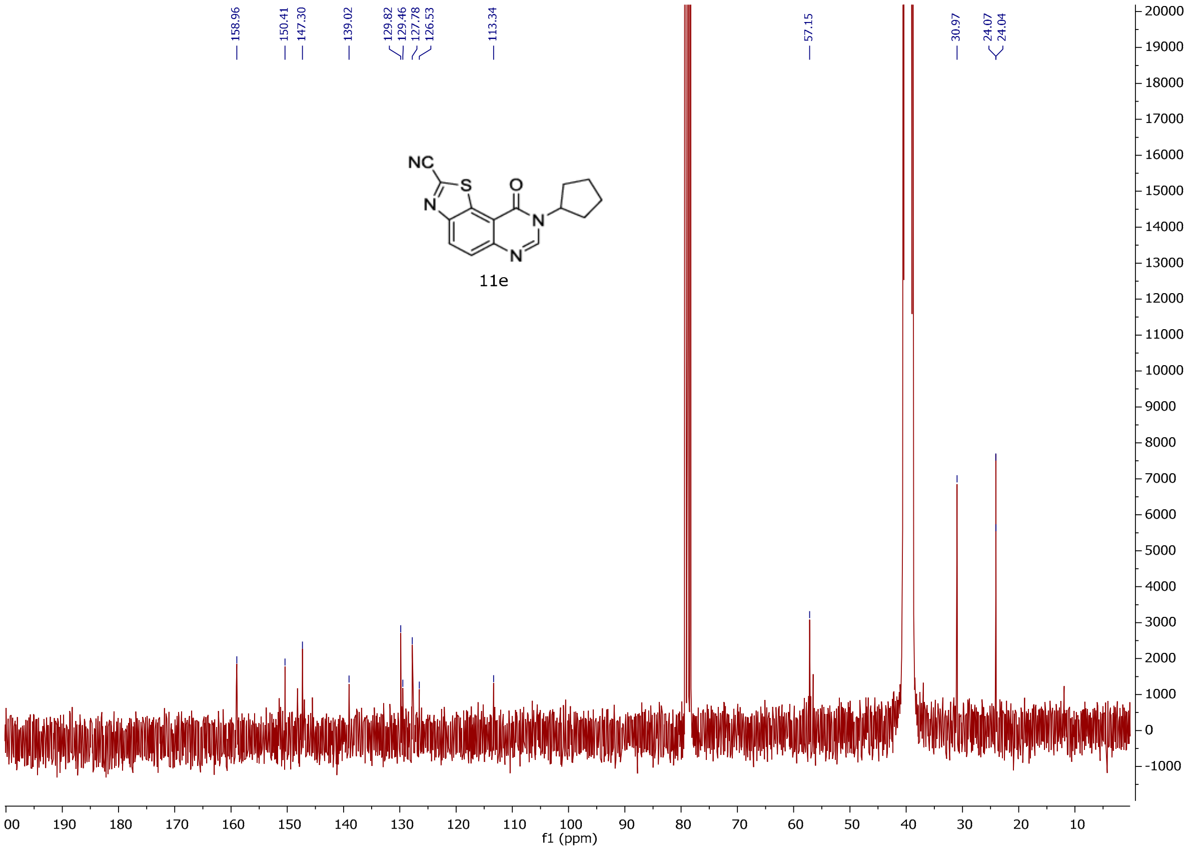

Figure S10. ^{13}C-NMR Compound **11e.**

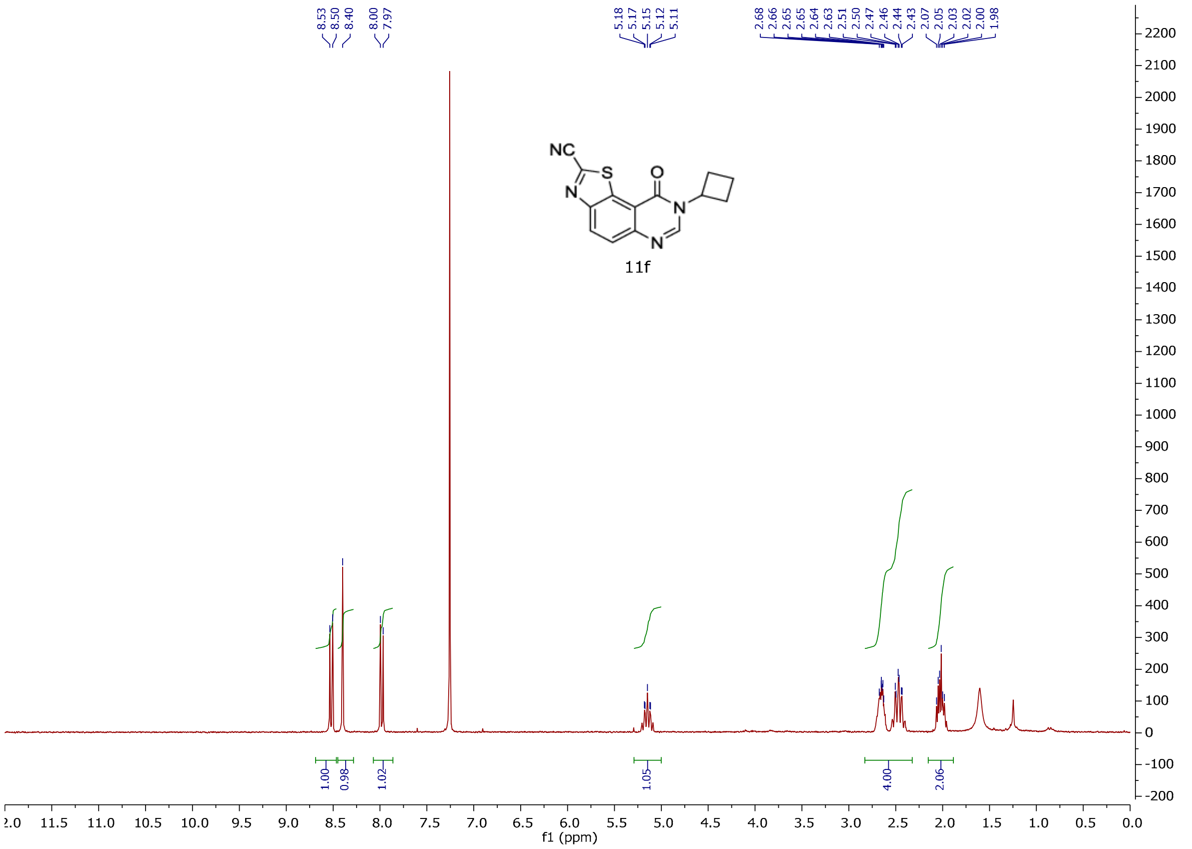

Figure S11. ^{1}H-NMR Compound **11f.**

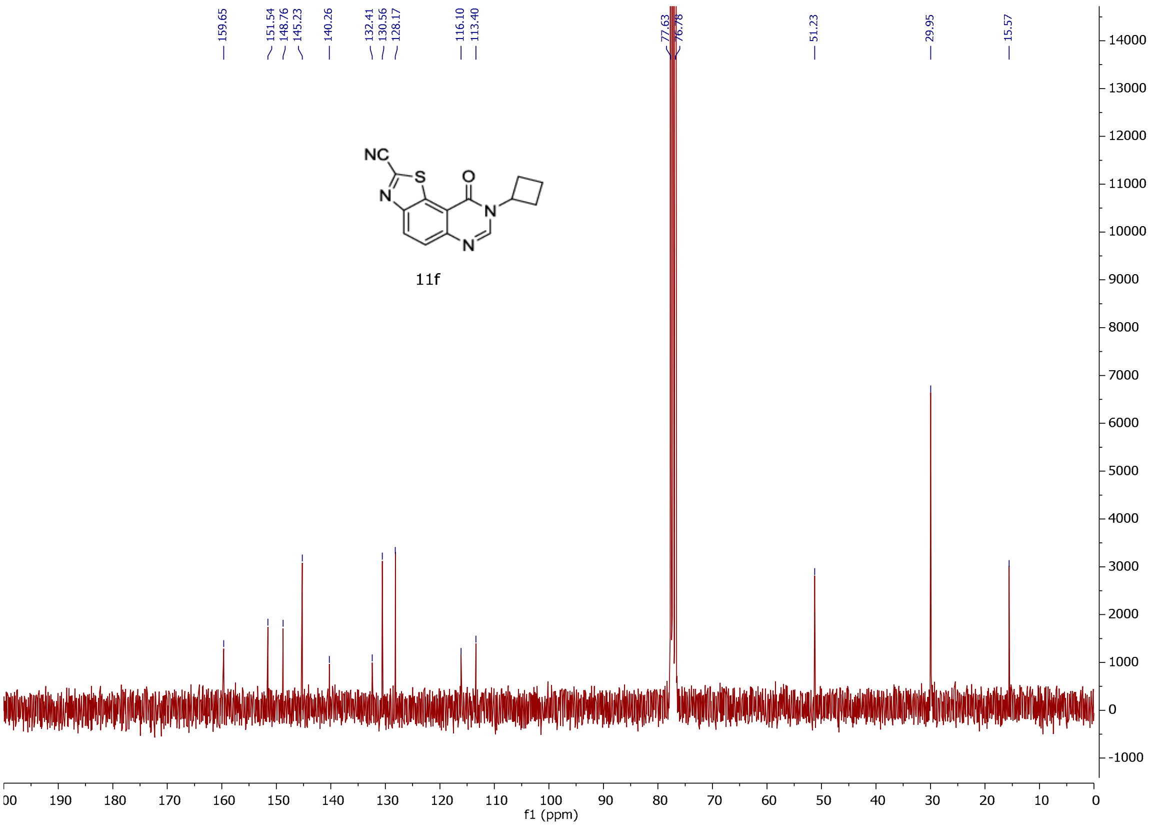

Figure S12. ^{13}C-NMR Compound **11f.**

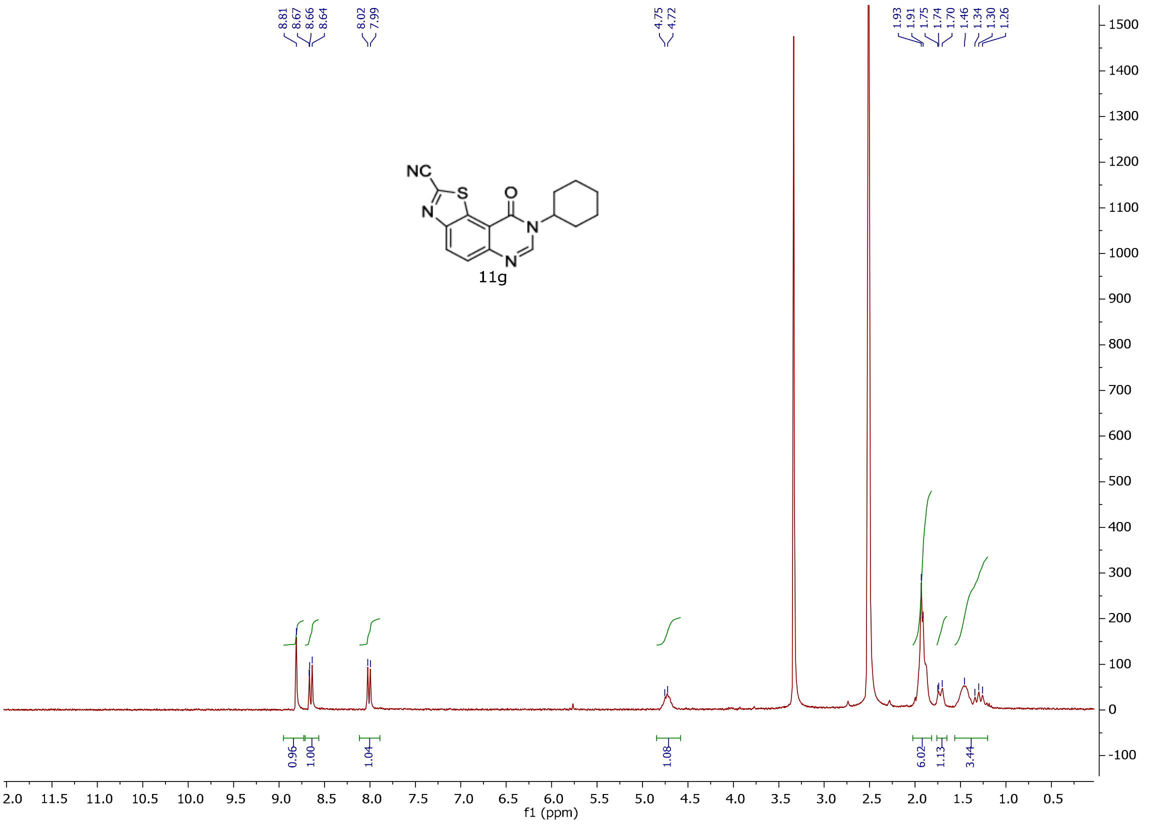

Figure 13 ^{1}H-NMR Compound **11g.**

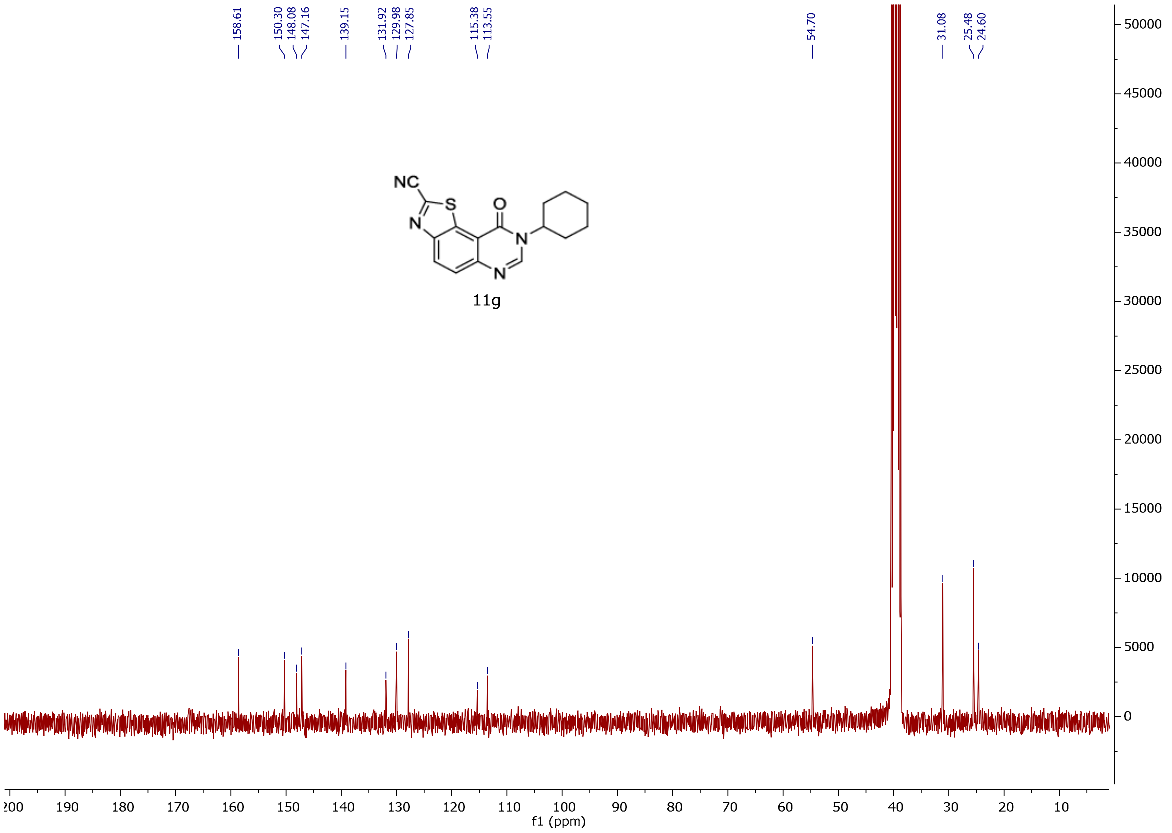

Figure S14. ^{13}C-NMR Compound **11h.**

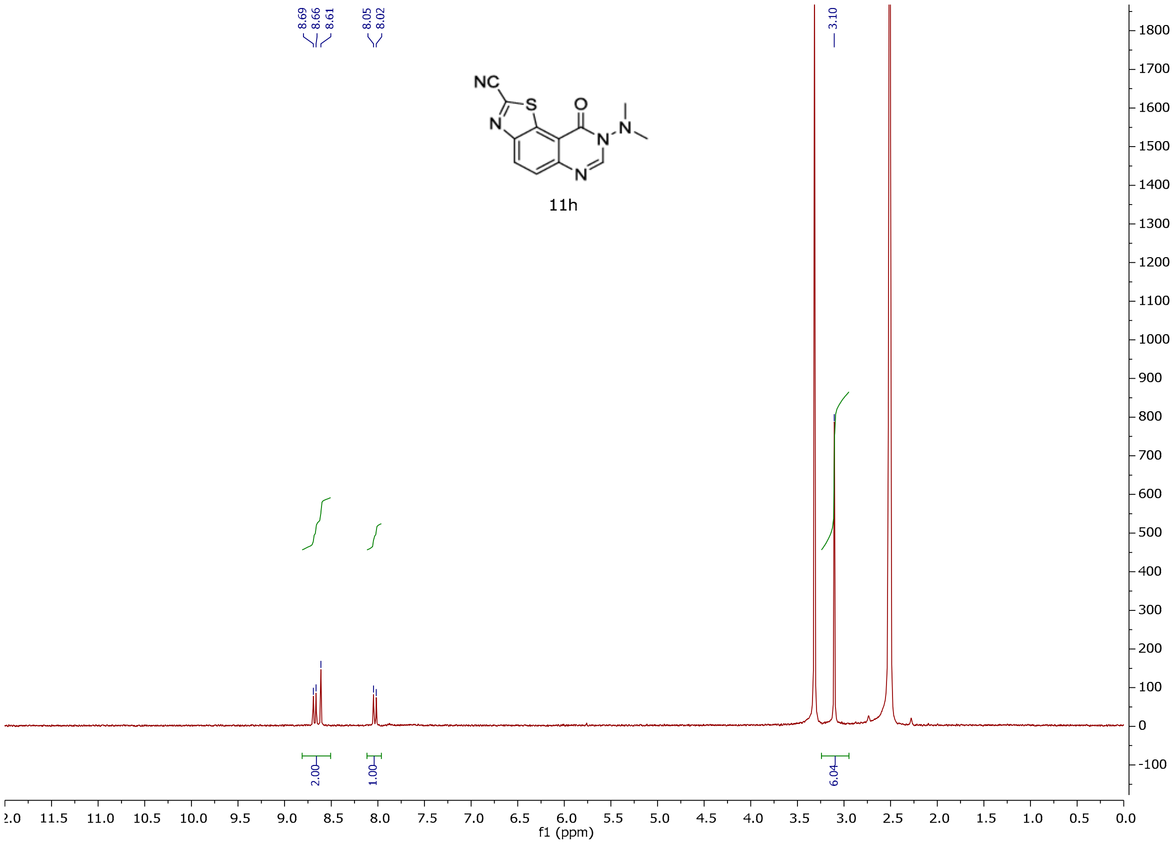

Figure S15. ^{1}H-NMR Compound **11h.**

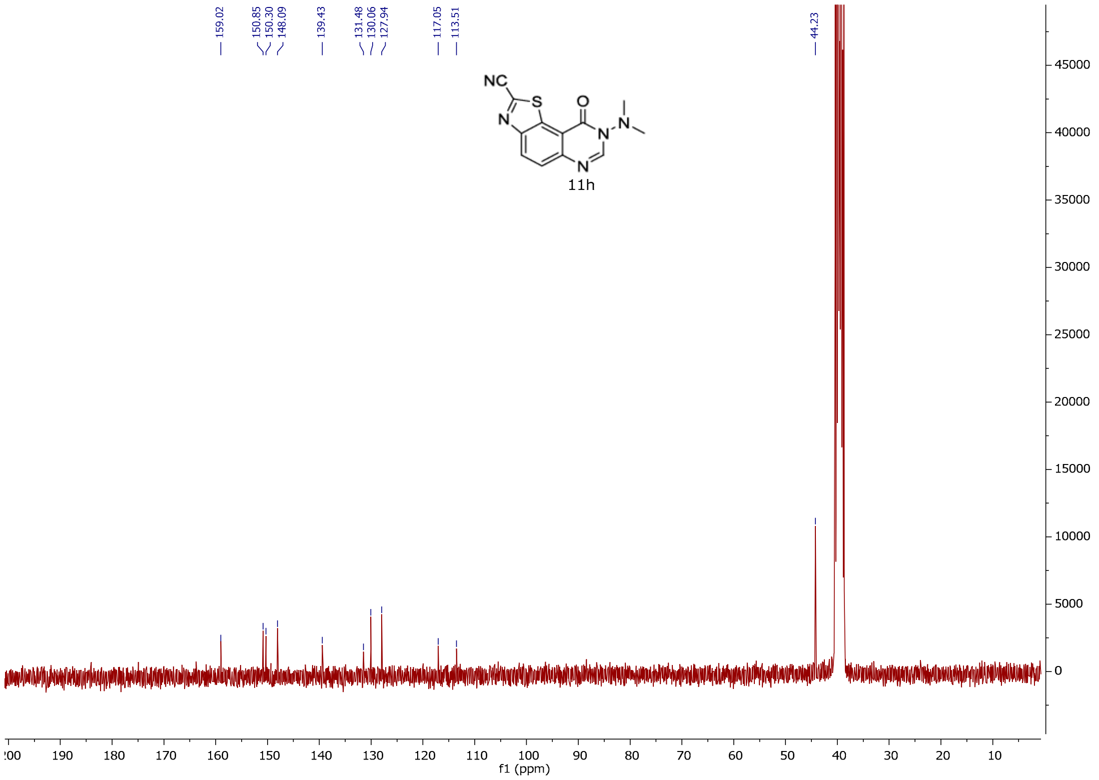

Figure S16. ^{13}C-NMR Compound **11h.**

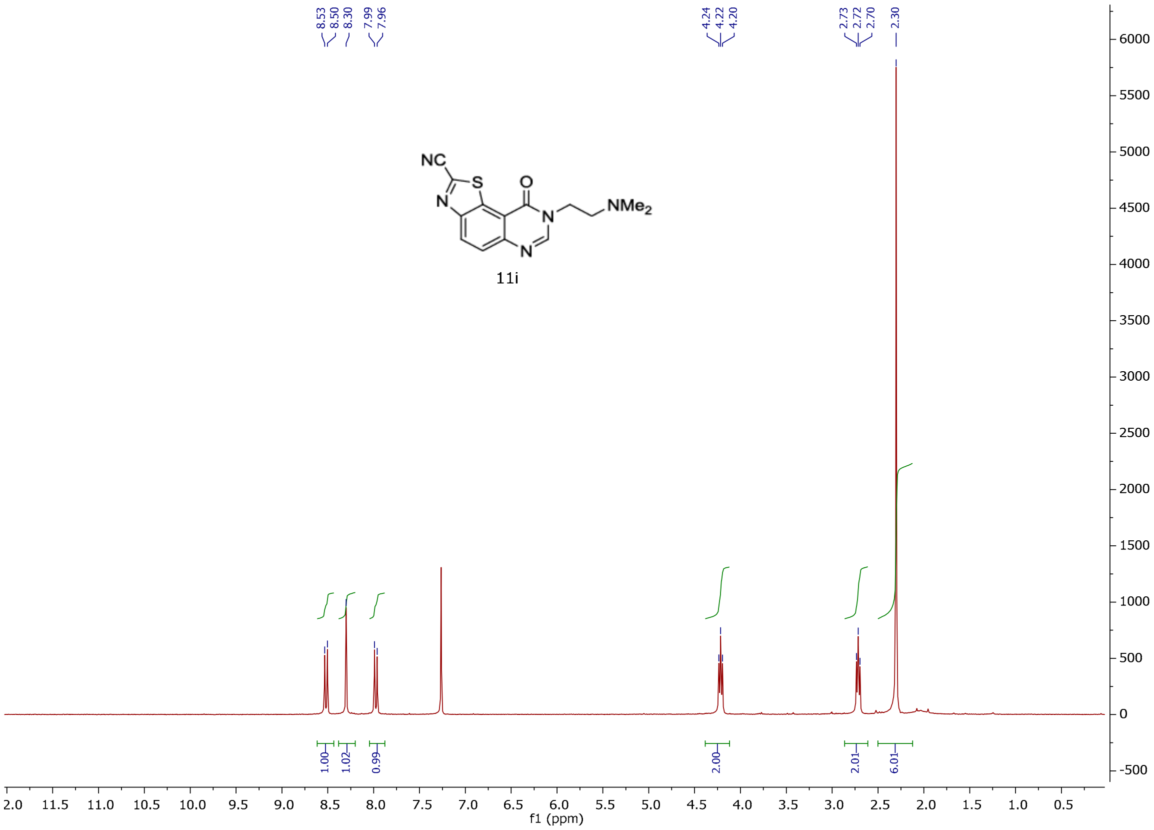

Figure S17. ^{1}H-NMR Compound **11i**.

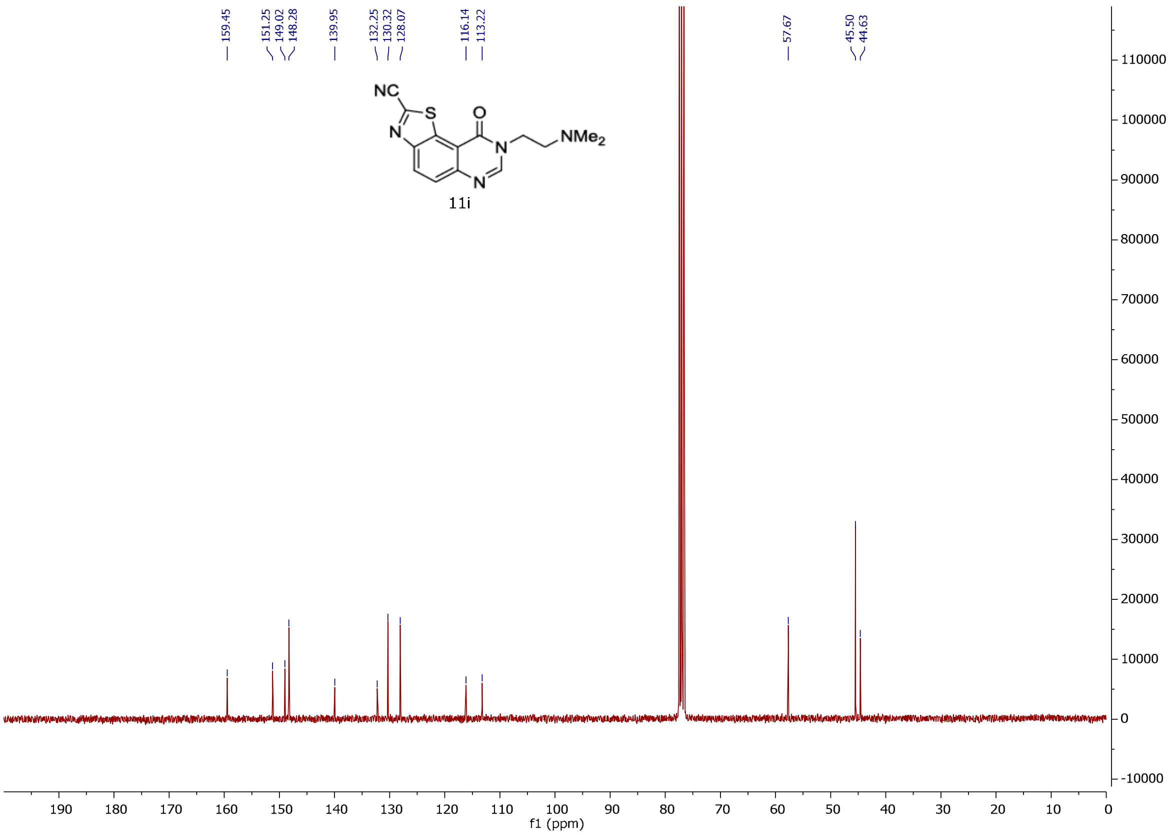

Figure S18. ^{13}C-NMR Compound **11i**.

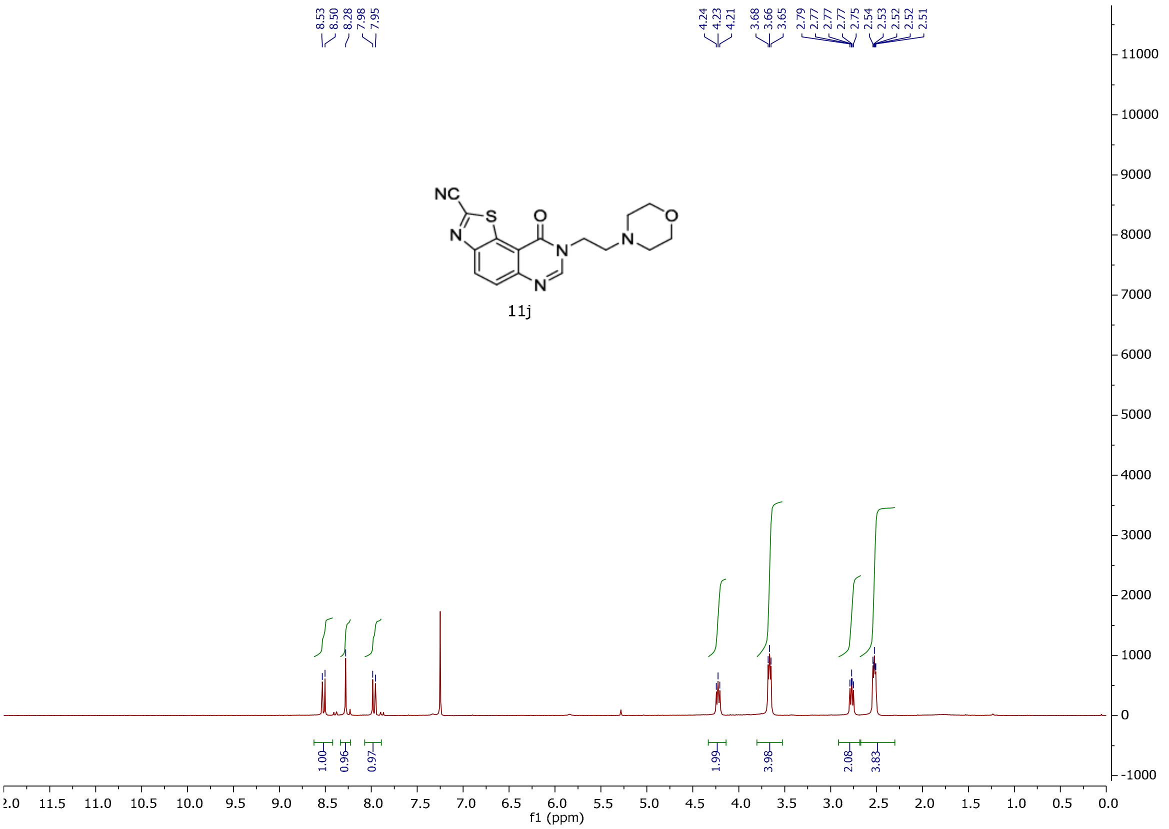

Figure S19. ^{1}H-NMR Compound **11j**.

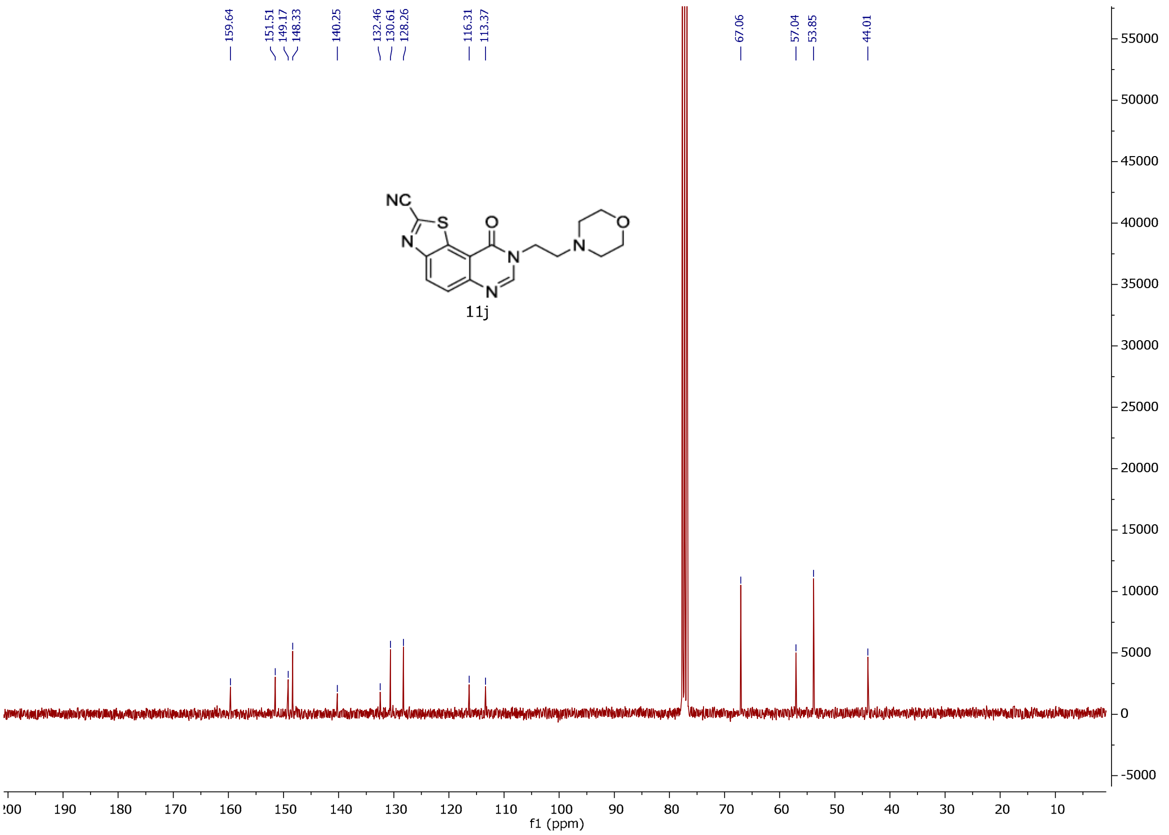

Figure S20. ^{13}C-NMR Compound **11i**.

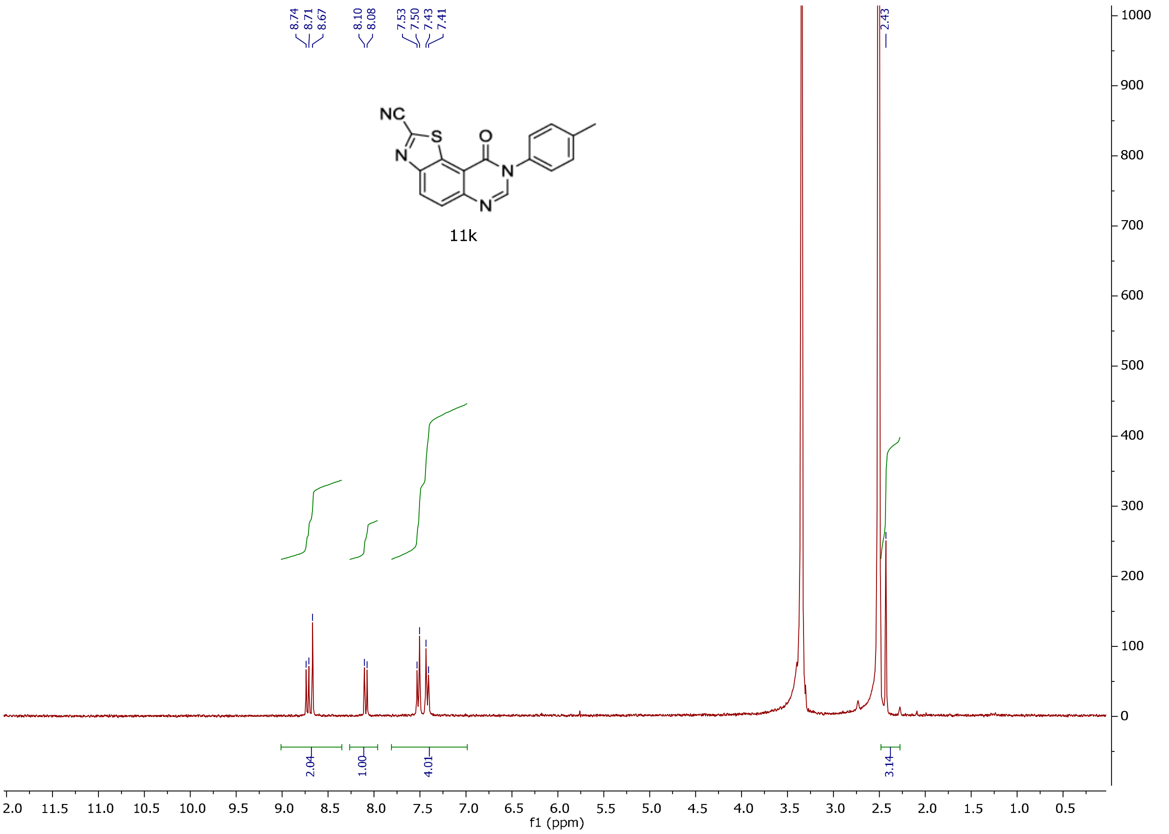

Figure S21. ^{1}H-NMR Compound **11k.**

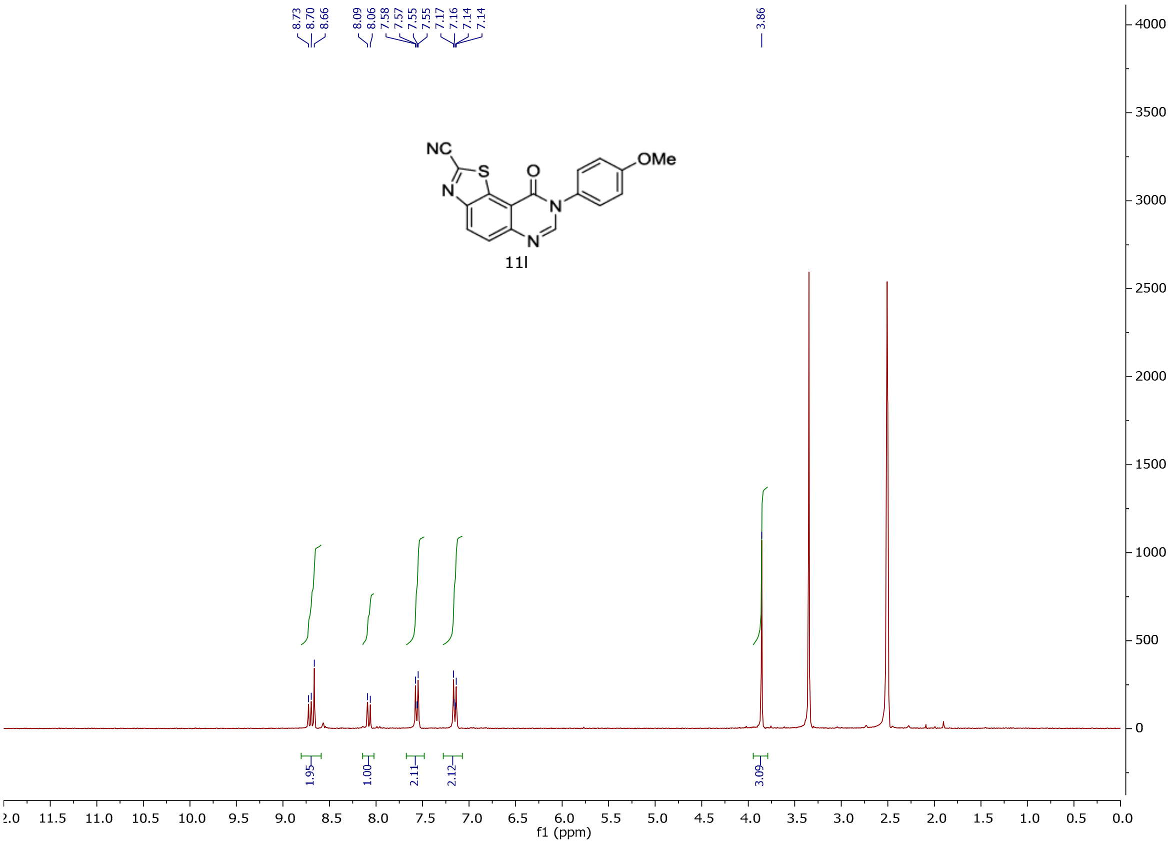

Figure S22. ^{1}H-NMR Compound **11l.**

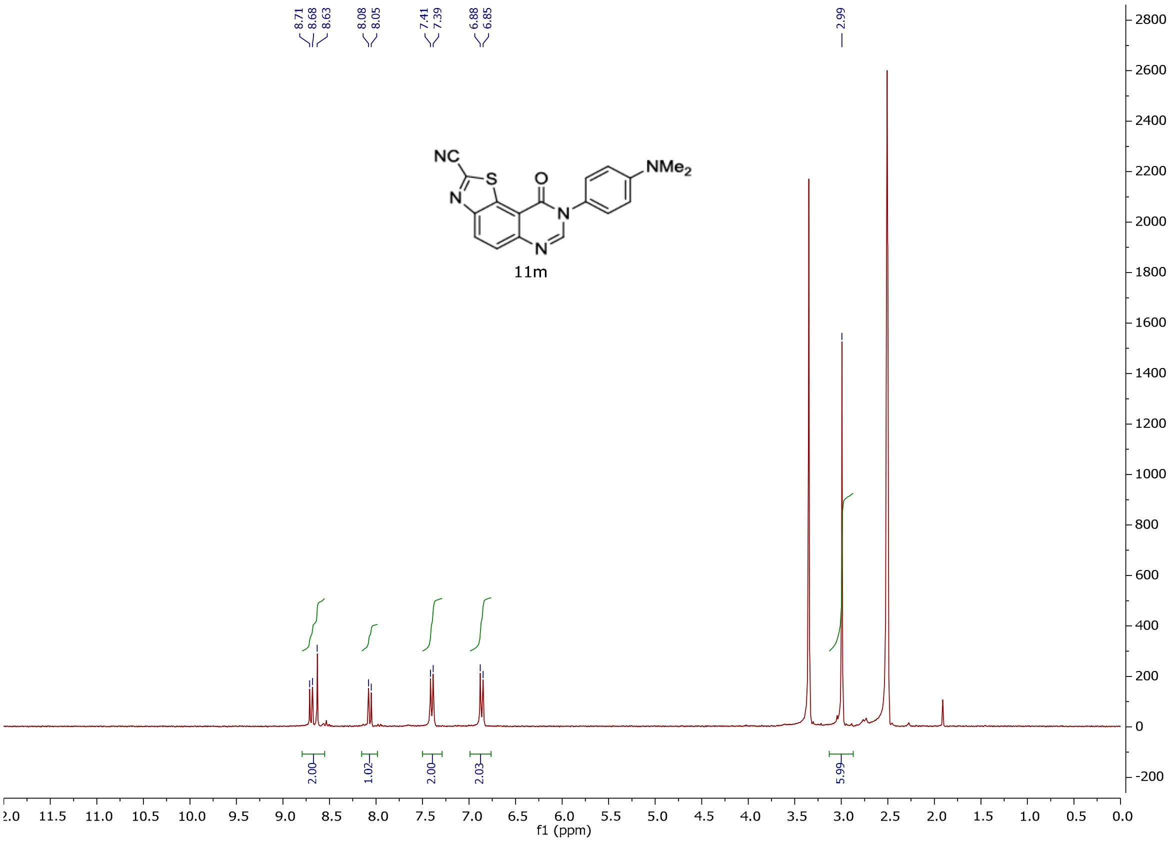

Figure S23. ^{1}H-NMR Compound **11m.**

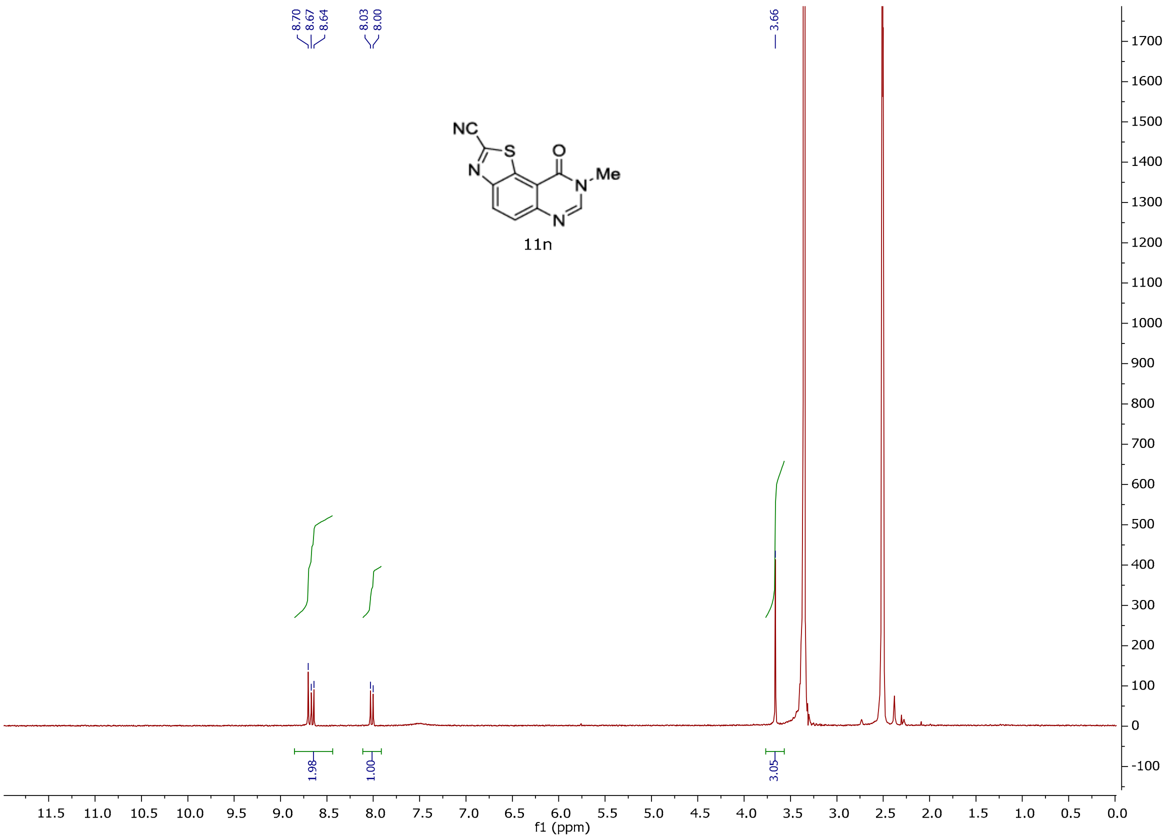

Figure S24. ^{1}H-NMR Compound **11n.**

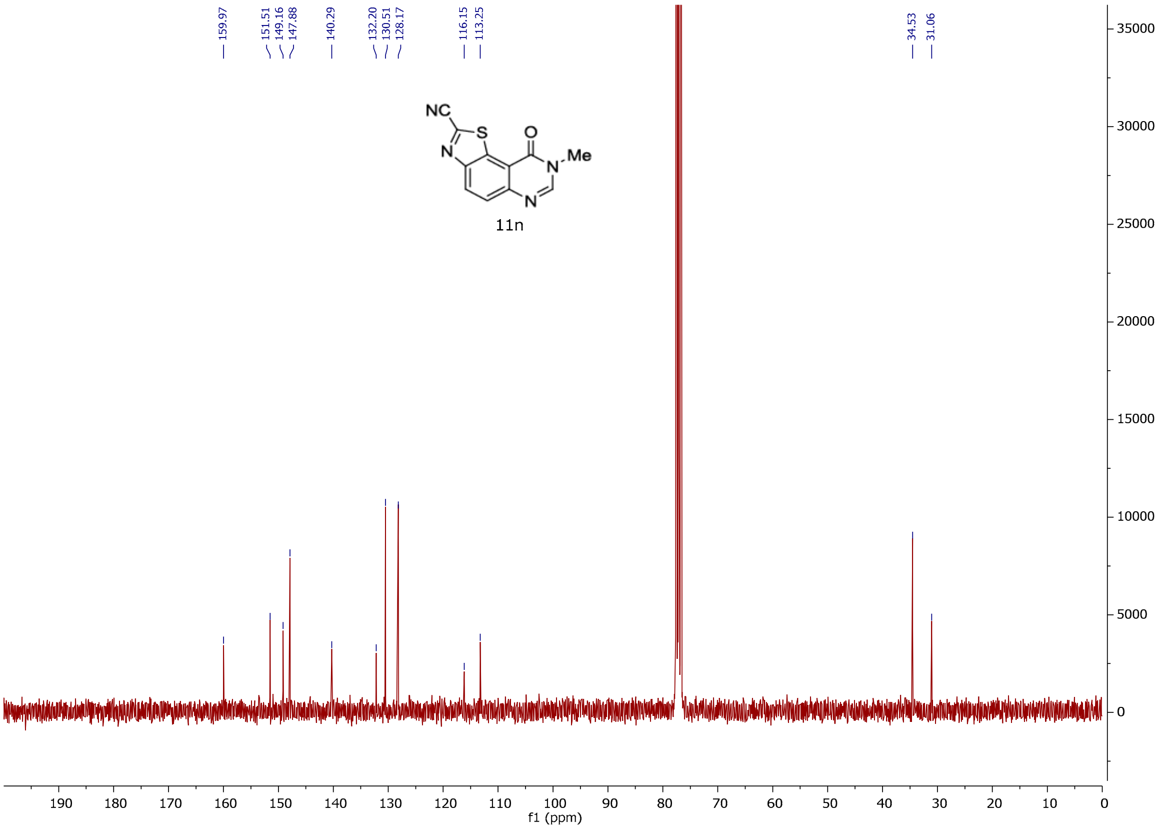

Figure S25. ^{13}C-NMR Compound **11n.**

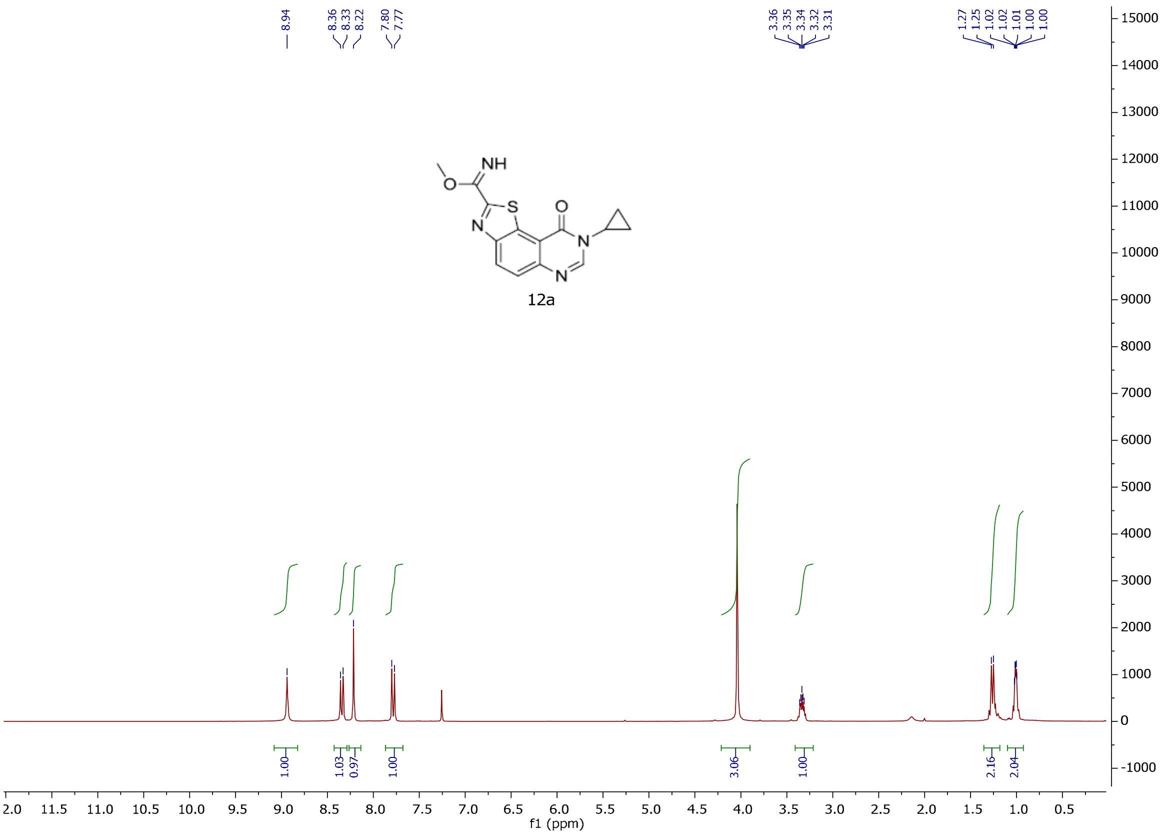

Figure S26. ^{1}H-NMR Compound **12a.**

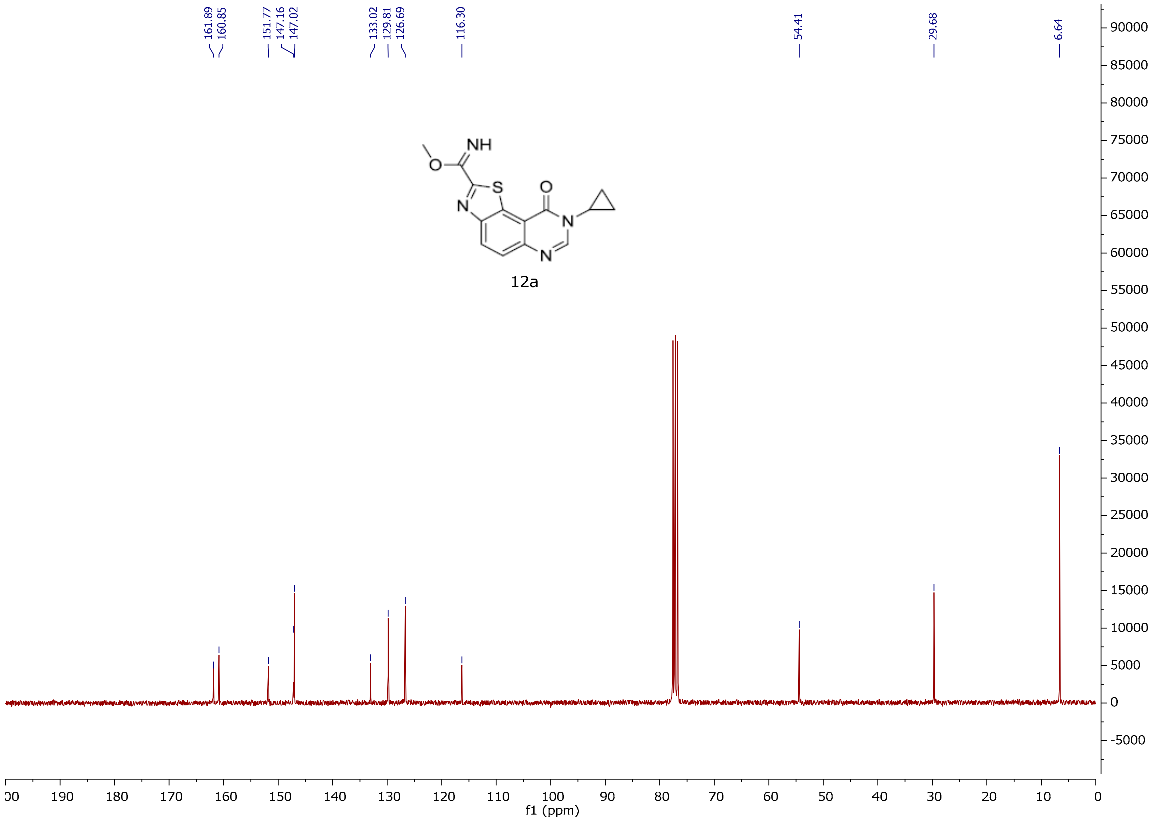

Figure S27. ^{13}C-NMR Compound **12a.**

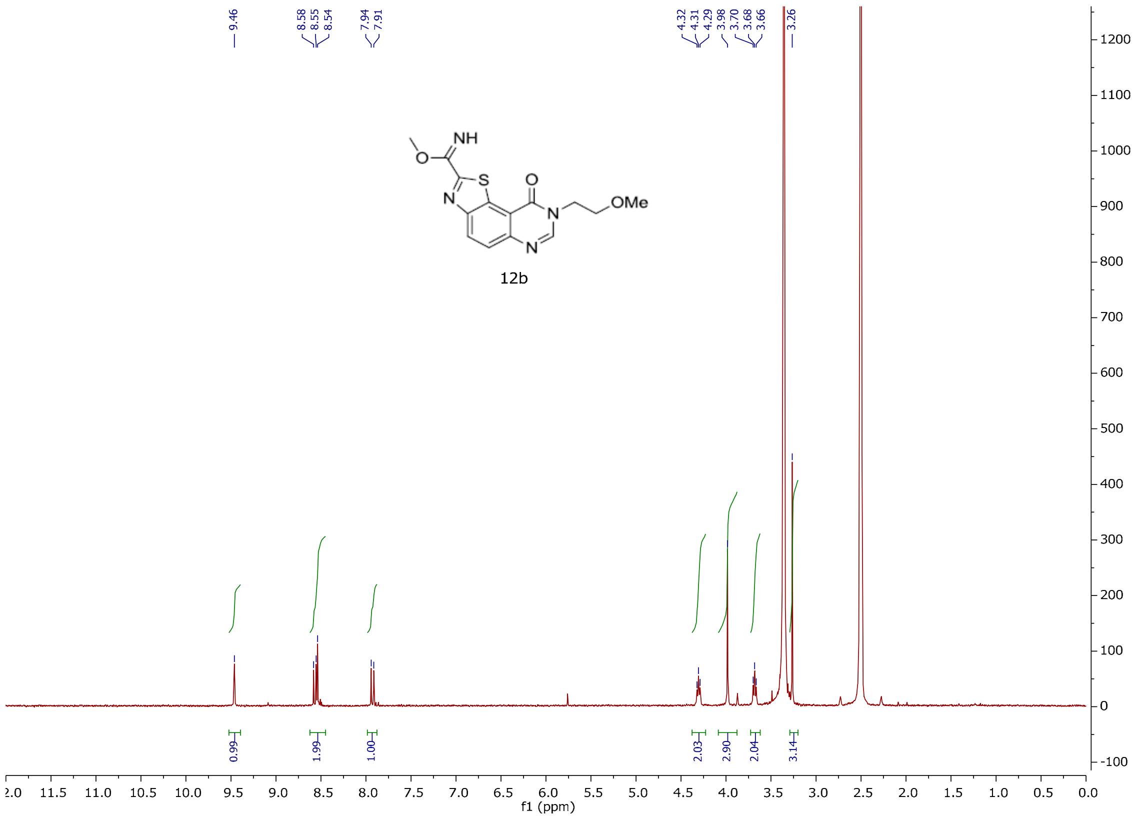

Figure S28. ^{1}H-NMR Compound **12b.**

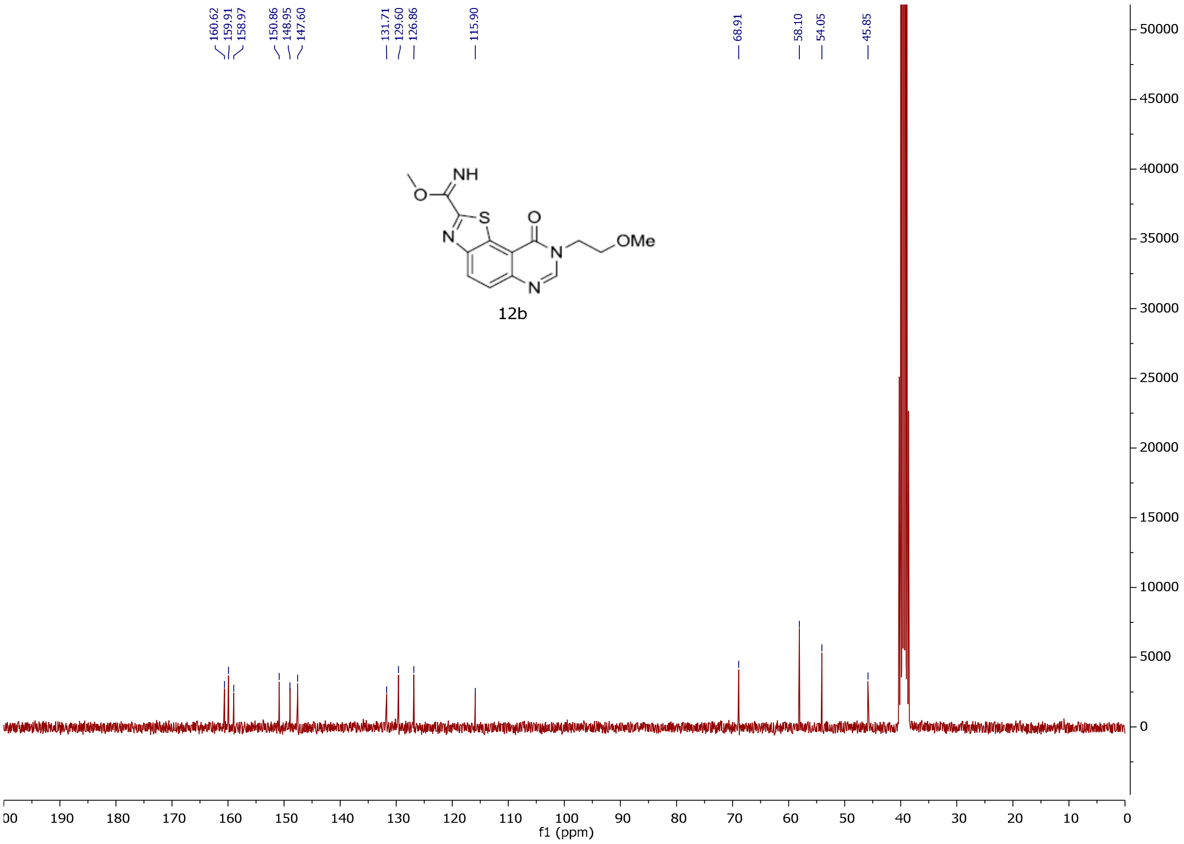

Figure S29. ^{13}C-NMR Compound **12b.**

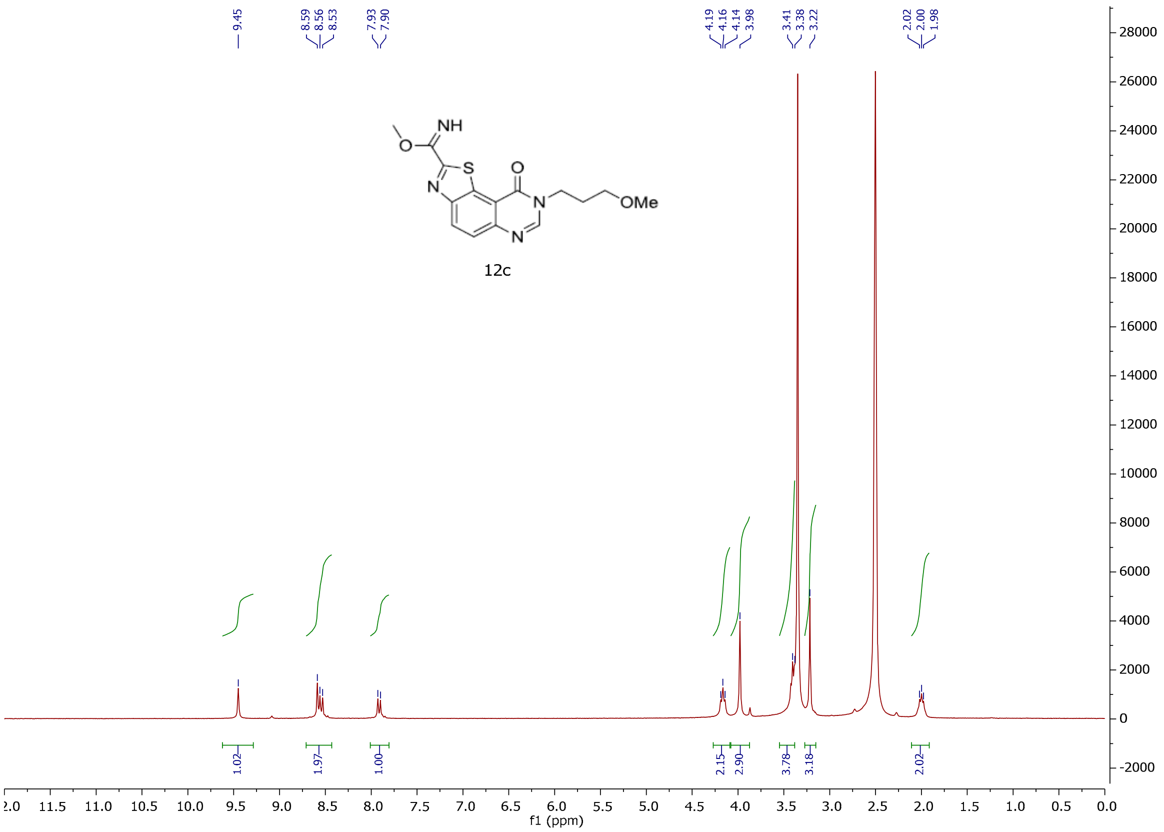

Figure S30. ^{1}H-NMR Compound **12c.**

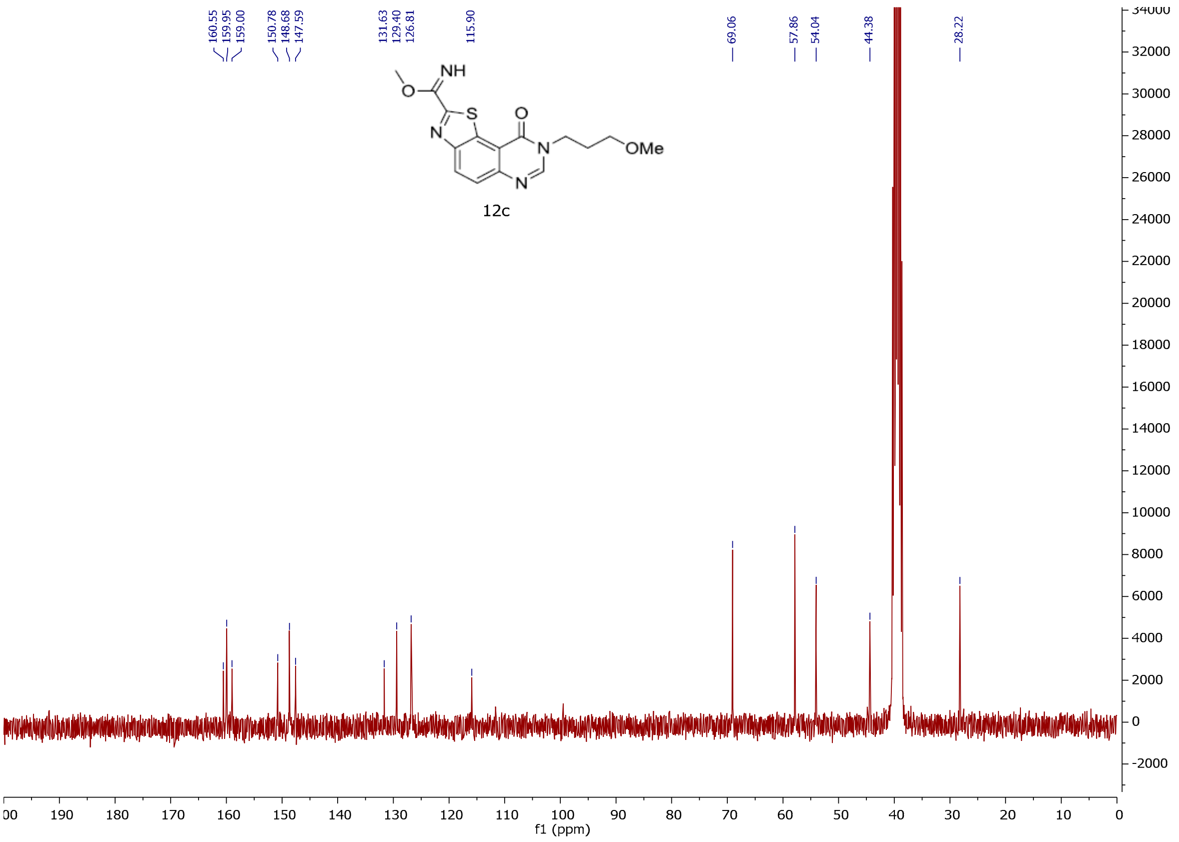

Figure S31. ^{1}H-NMR Compound **12c.**

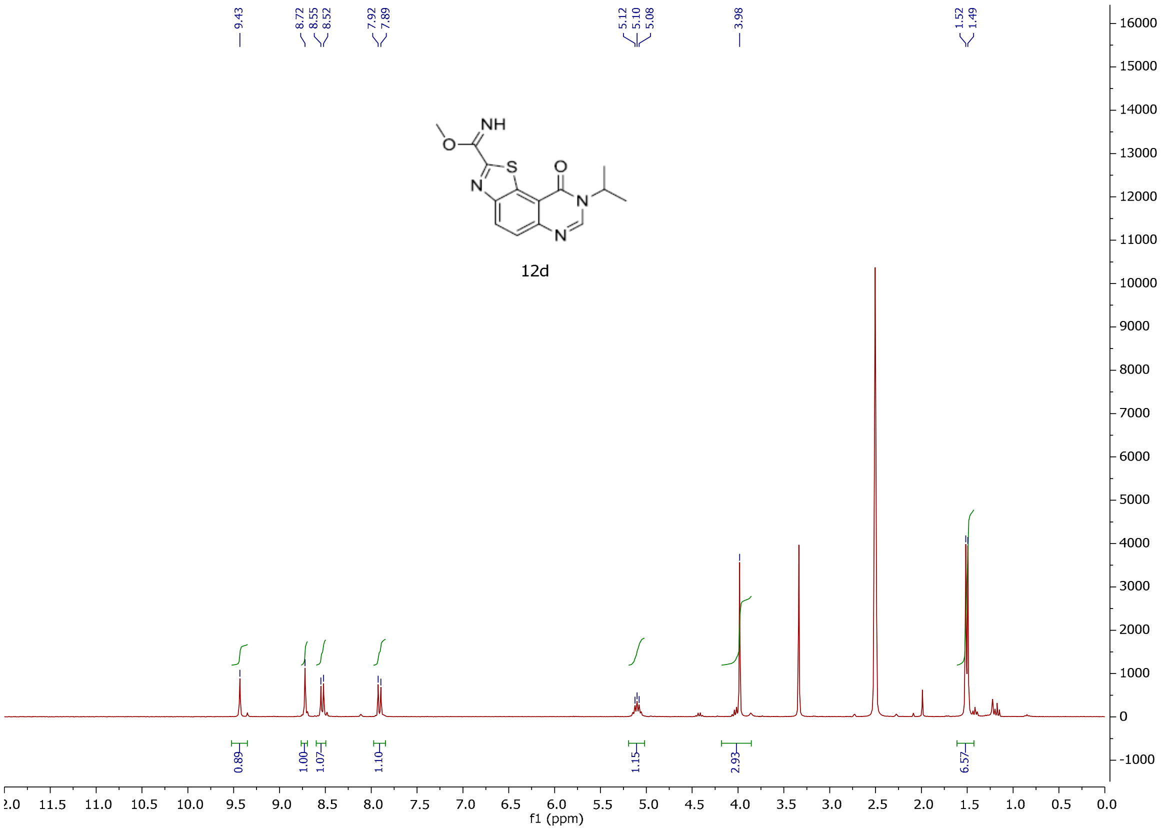

Figure S32. ^{1}H-NMR Compound **12d.**

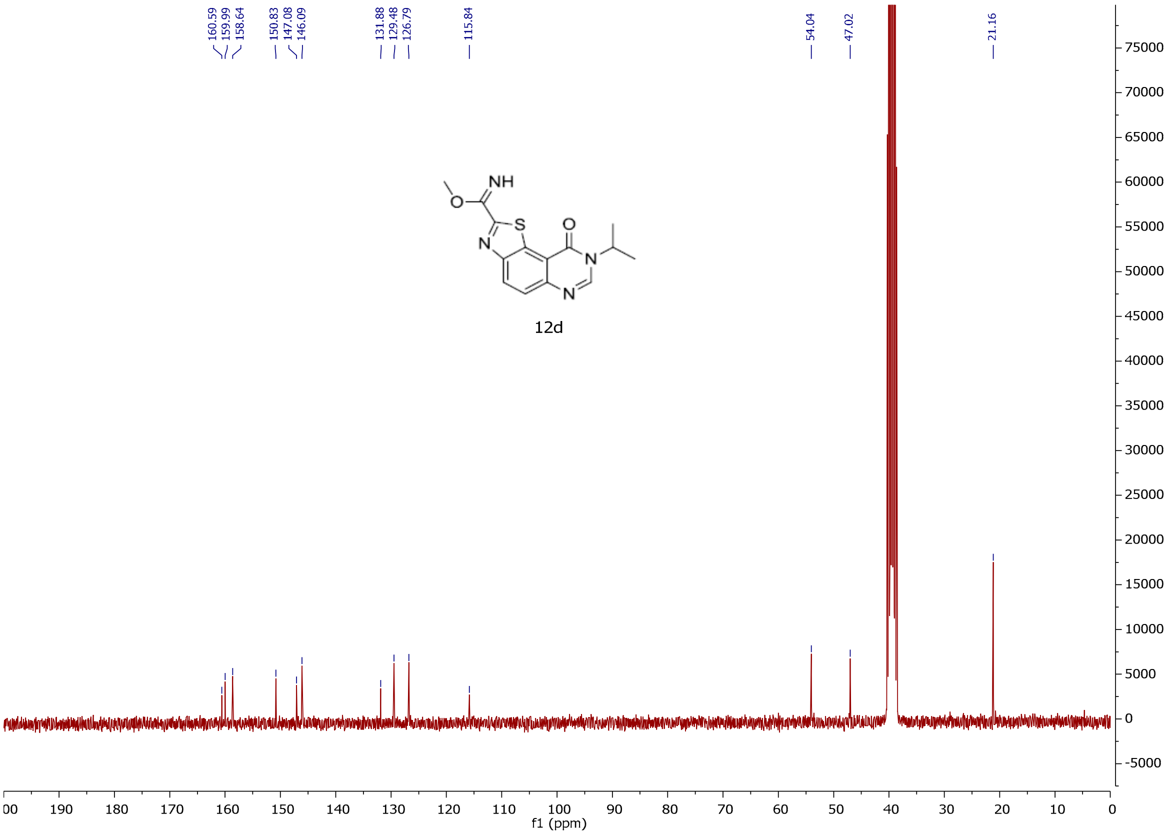

Figure S33. ^{13}C-NMR Compound **12d.**

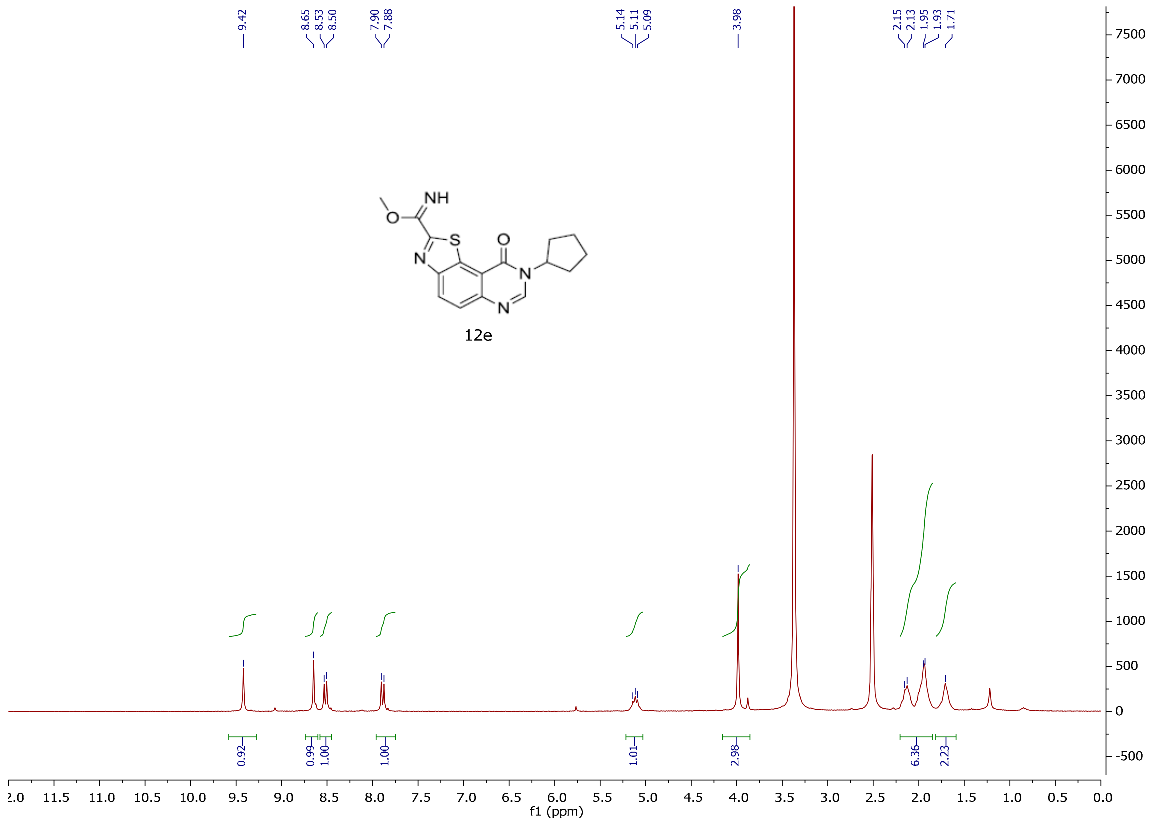

Figure S34. ^{1}H-NMR Compound **12e.**

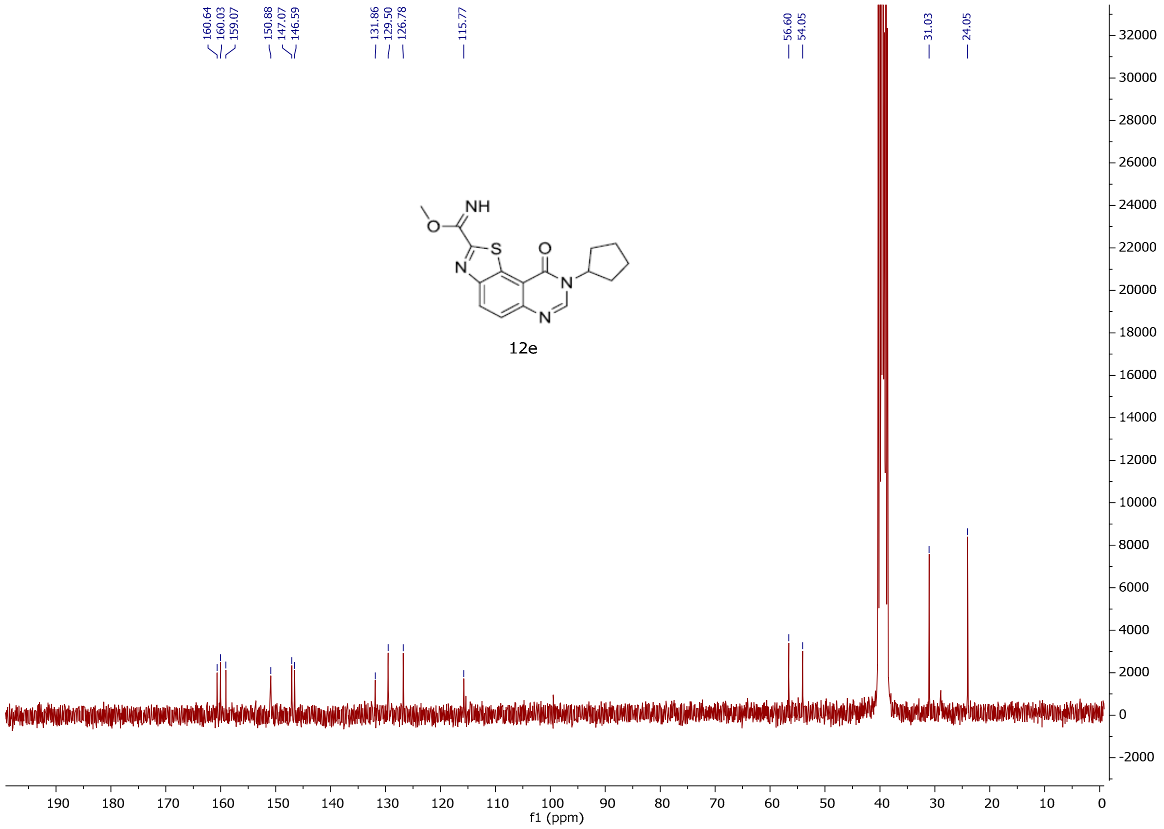

Figure S35. ^{13}C-NMR Compound **12e.**

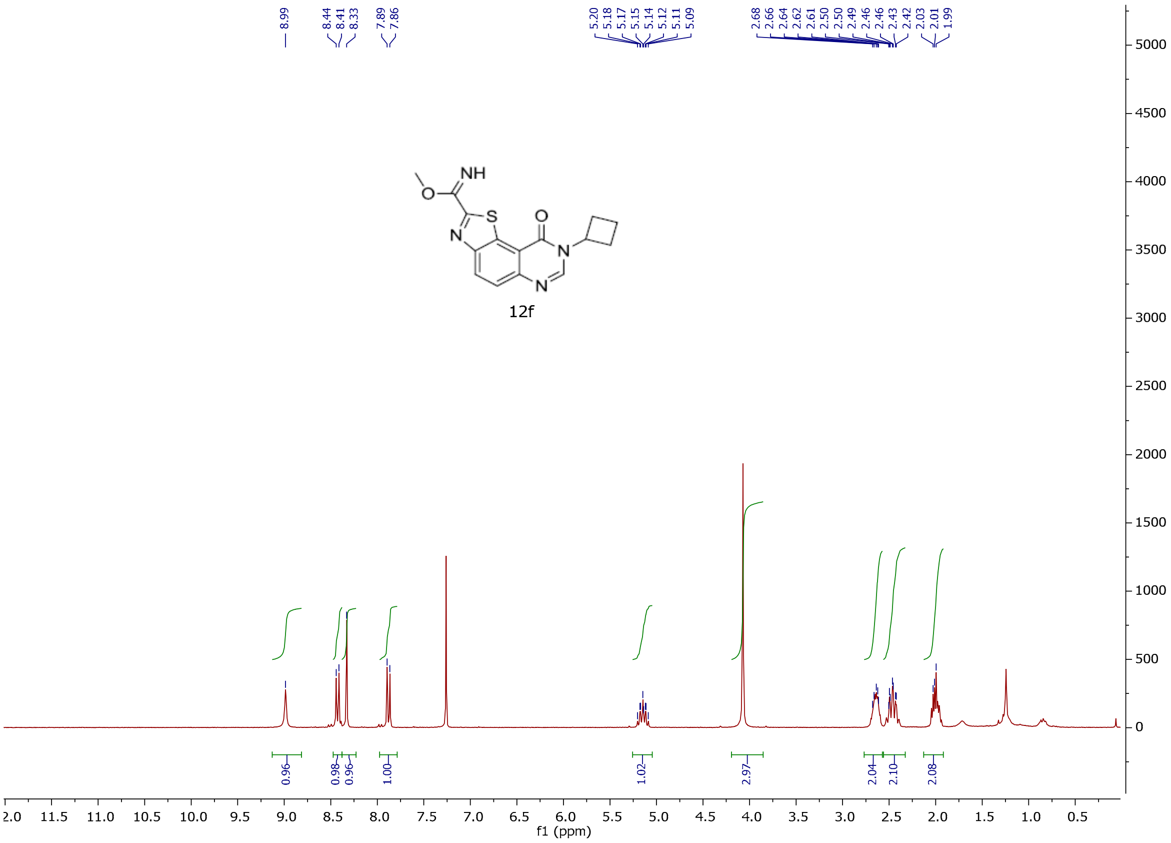

Figure S36. ^{1}H-NMR Compound **12f.**

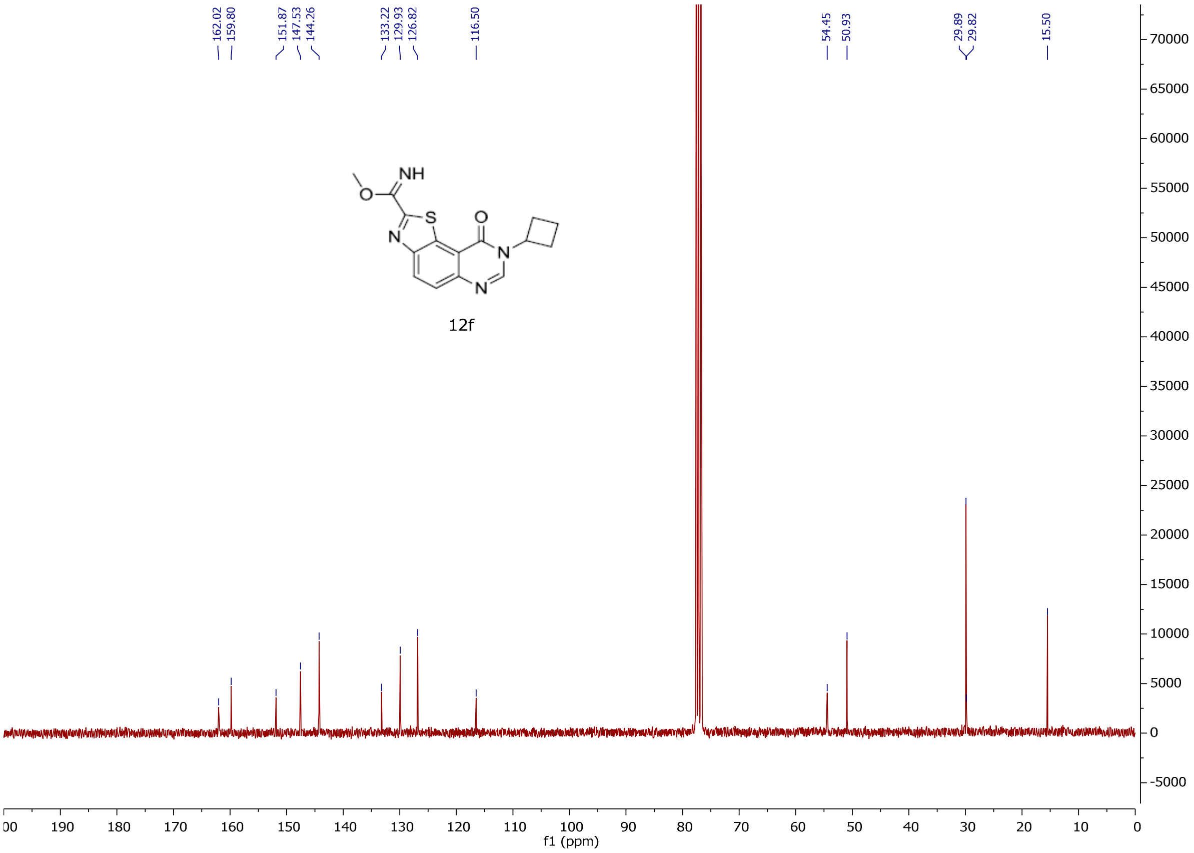

Figure S37. ^{13}C-NMR Compound **12f.**

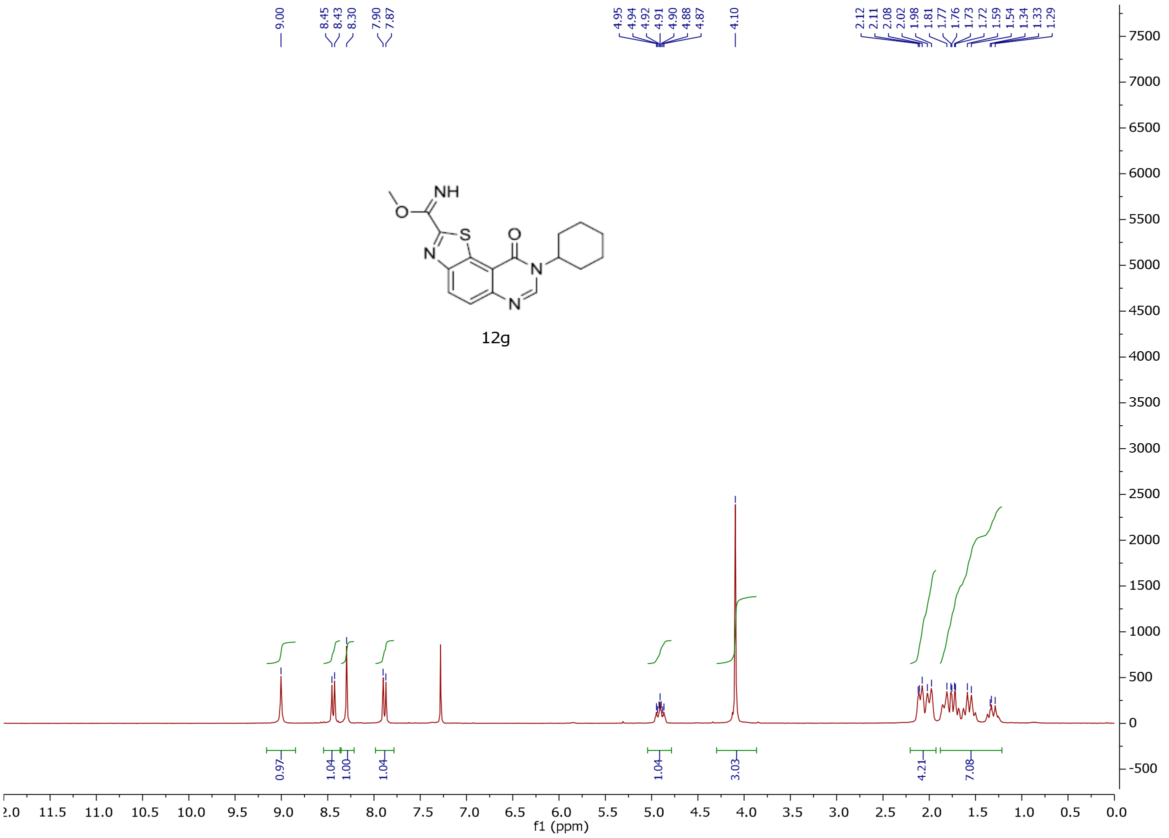

Figure S38. ^{1}H-NMR Compound **12g.**

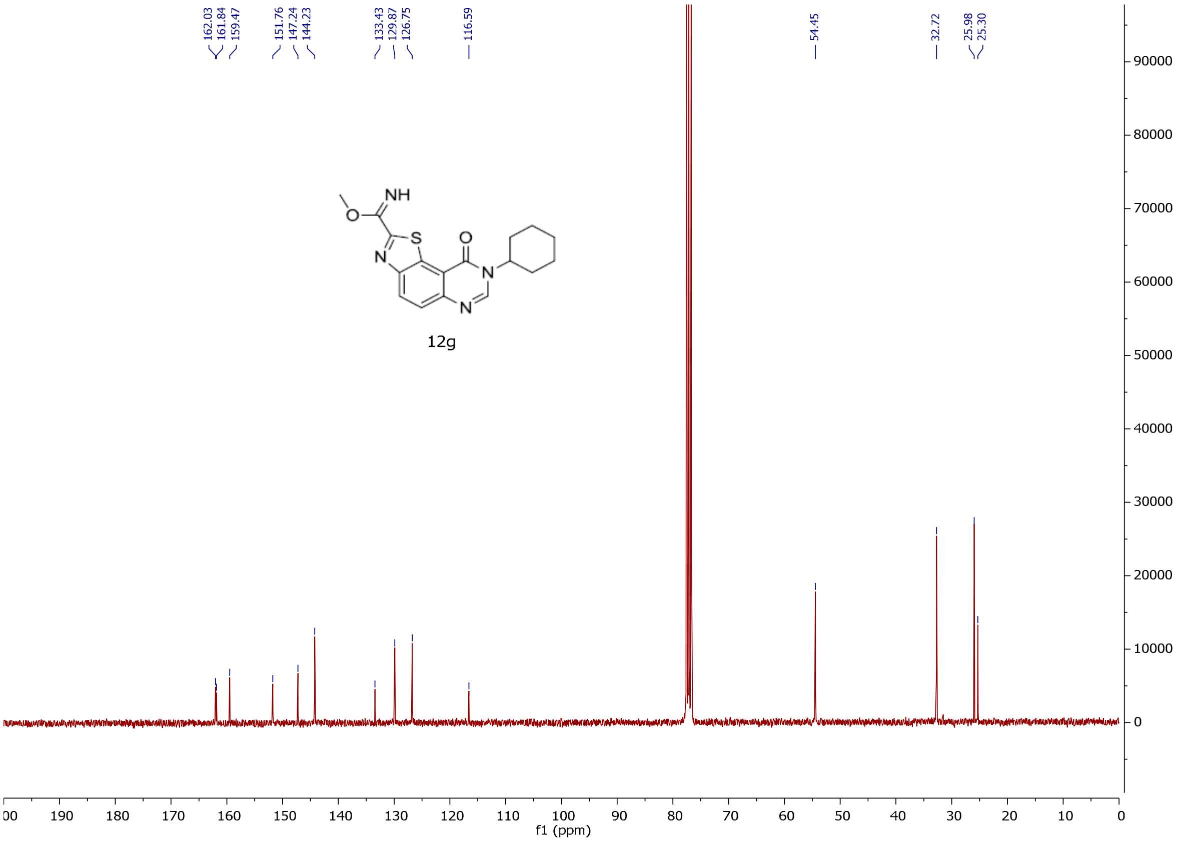

Figure S39. ^{13}C-NMR Compound **12g.**

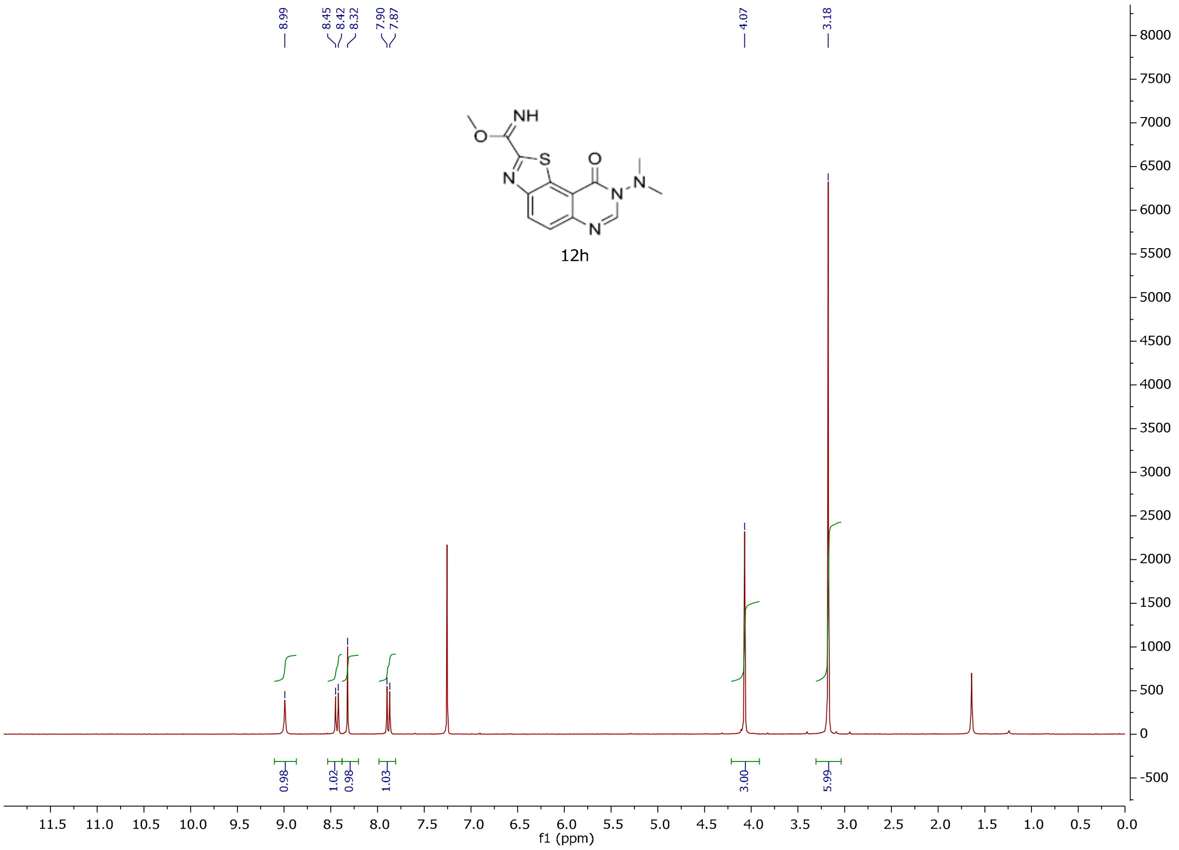

Figure S40. ^{1}H-NMR Compound **12h.**

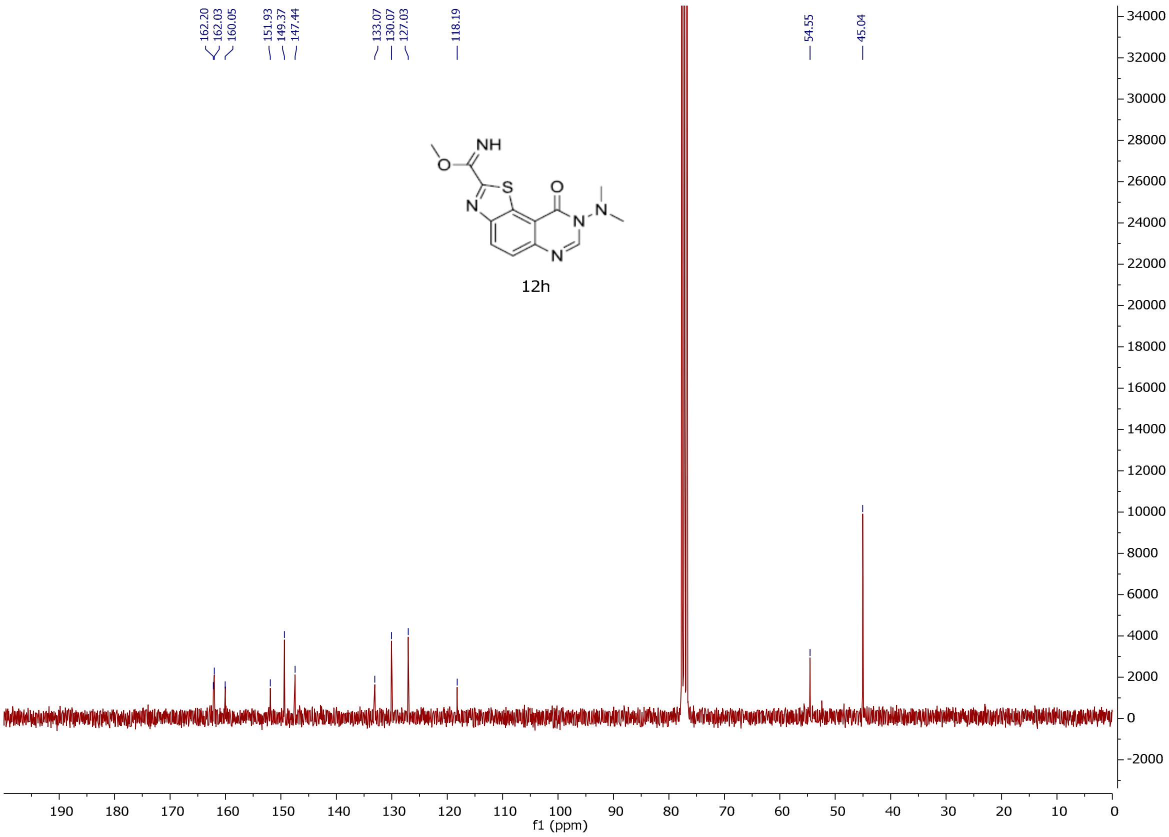

Figure S41. ^{13}C-NMR Compound **12h.**

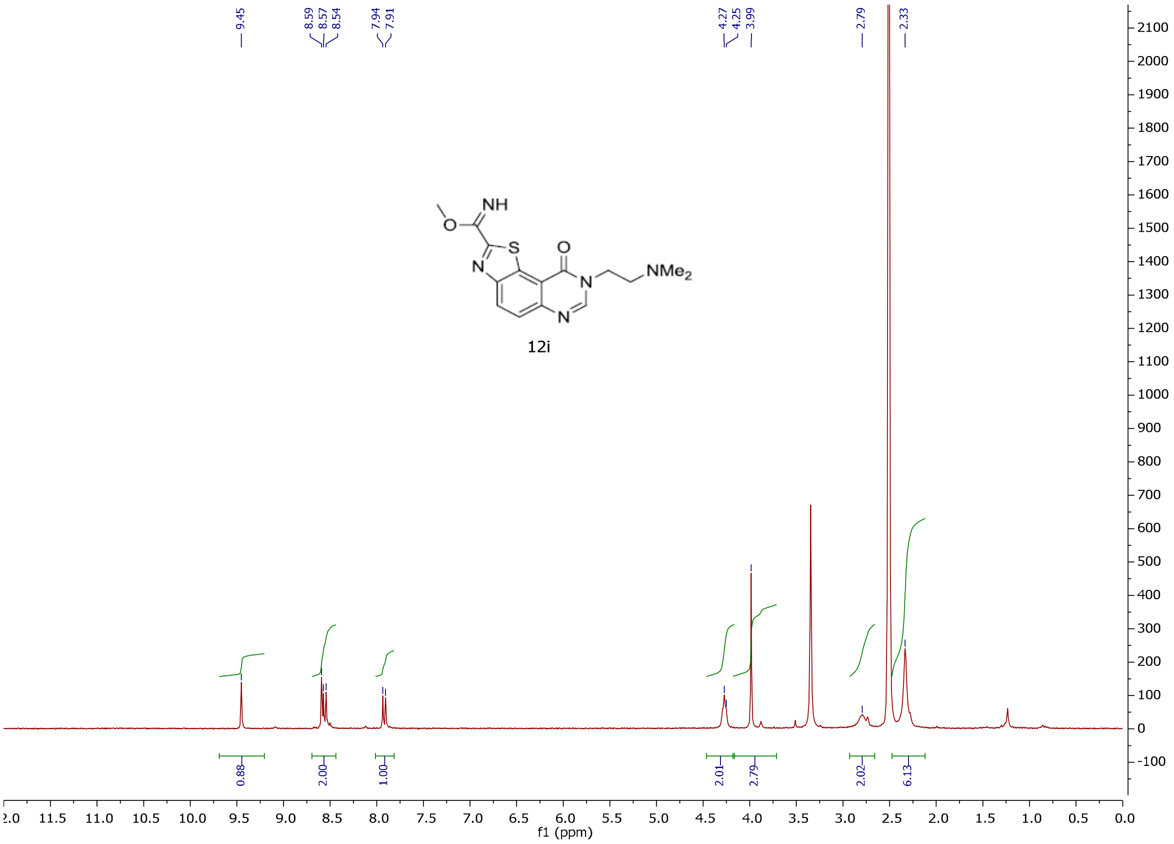

Figure S42. ^{1}H-NMR Compound **12i**.

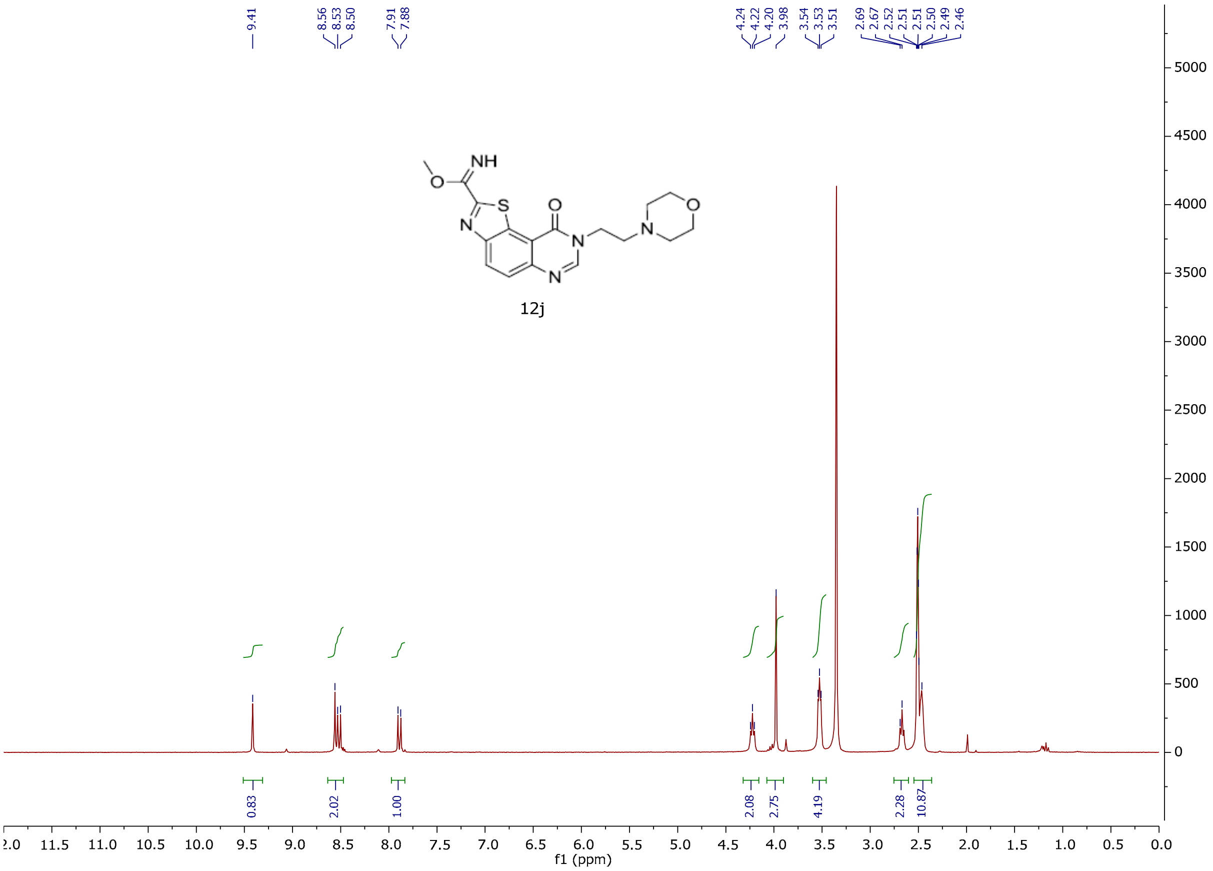

Figure S43. ^{1}H-NMR Compound **12j.**

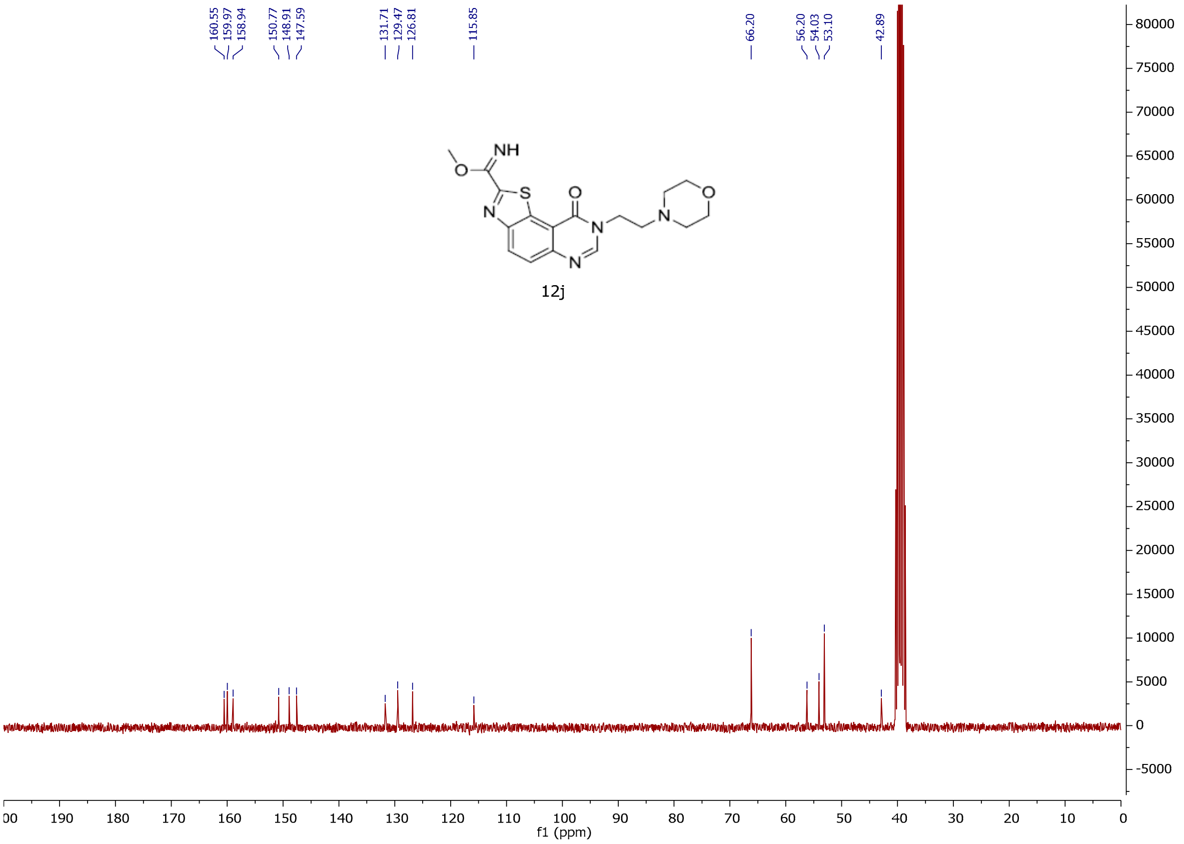

Figure S44. ^{13}C-NMR Compound **12j.**

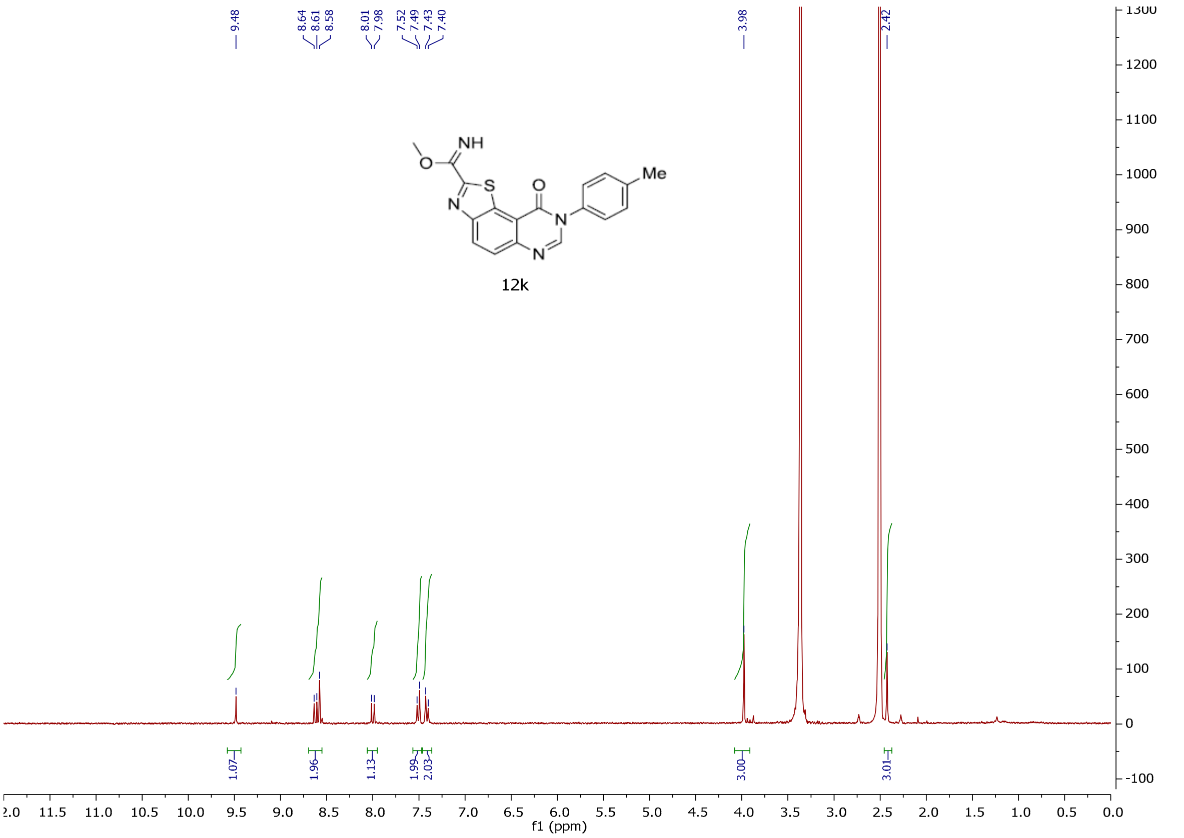

Figure S45. ^{1}H-NMR Compound **12k.**

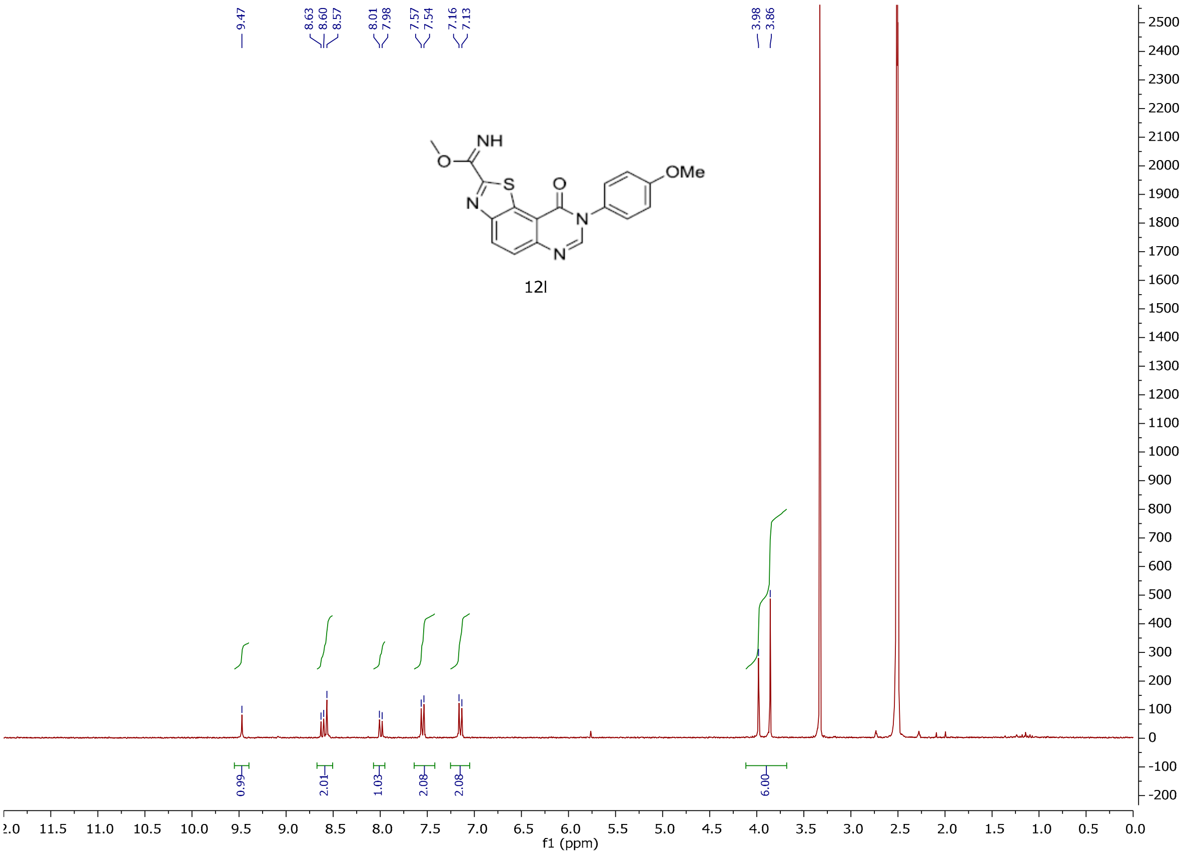

Figure S46. ^{1}H-NMR Compound **12l.**

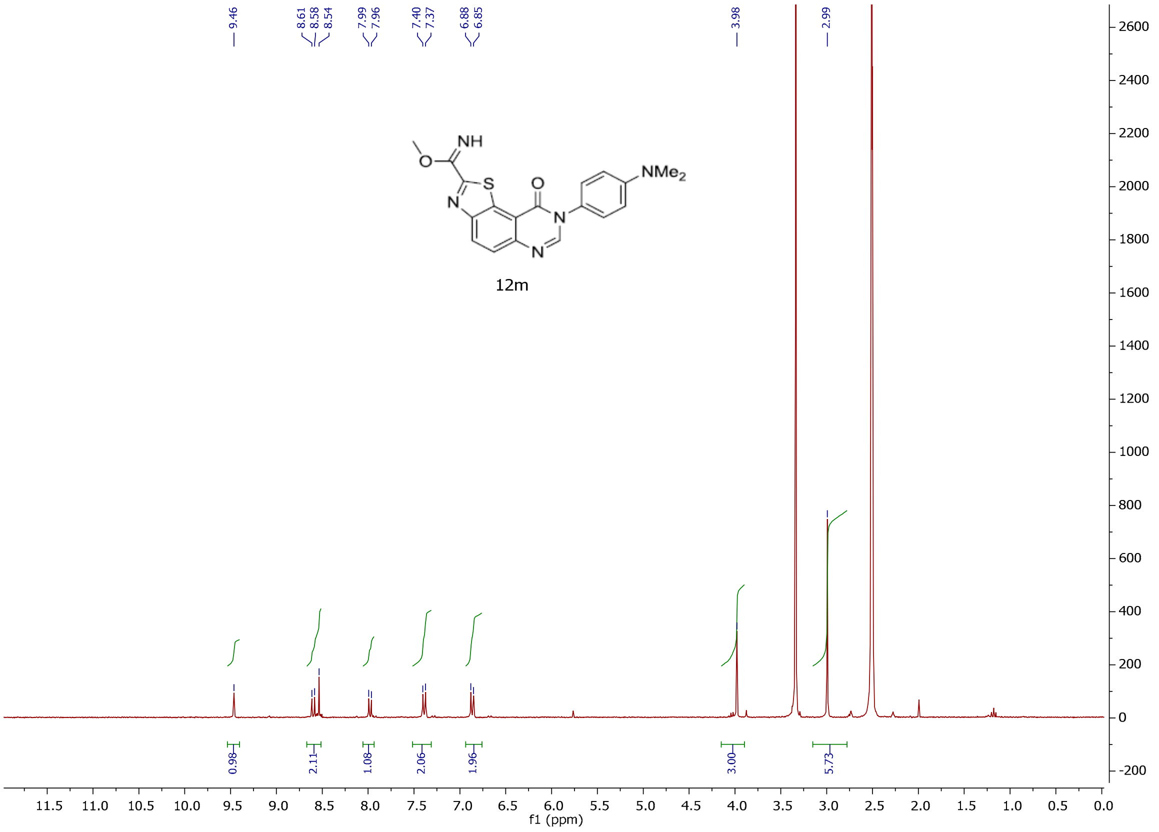

Figure S47. ^{1}H-NMR Compound **12l.**

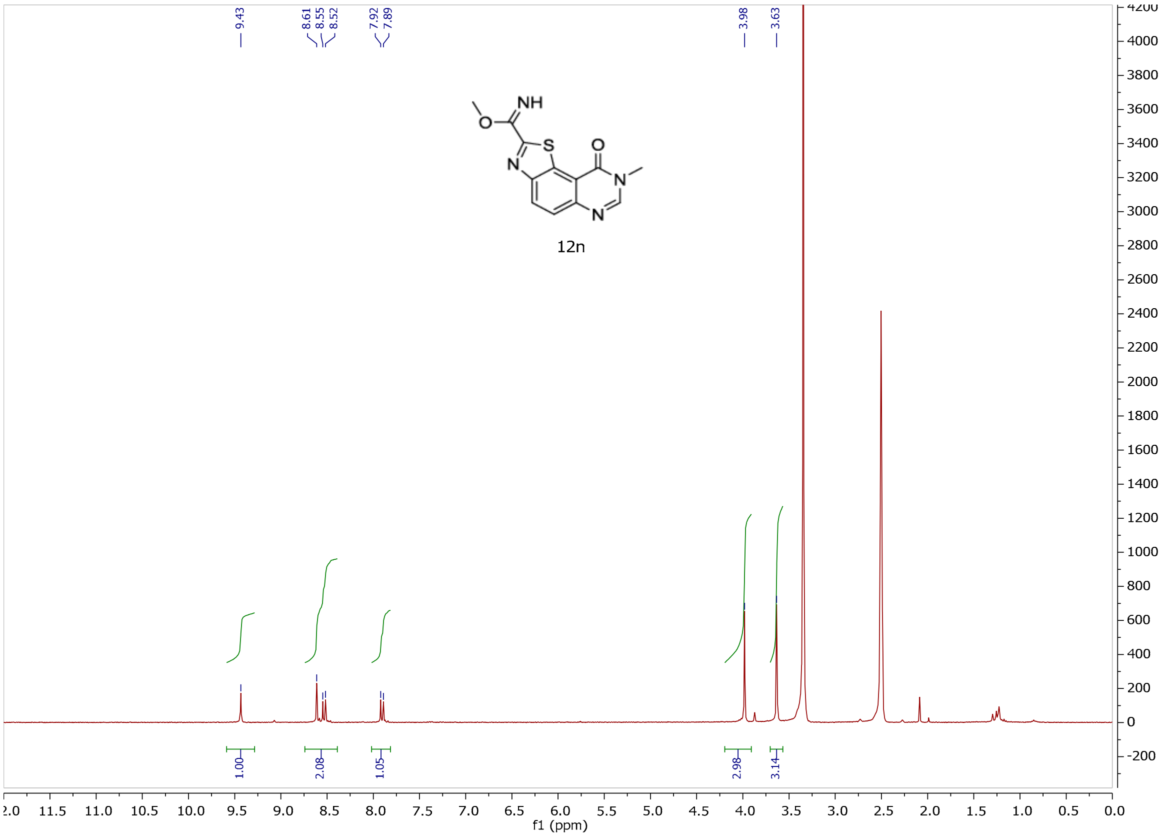

Figure S48. ^{1}H-NMR Compound **12n.**

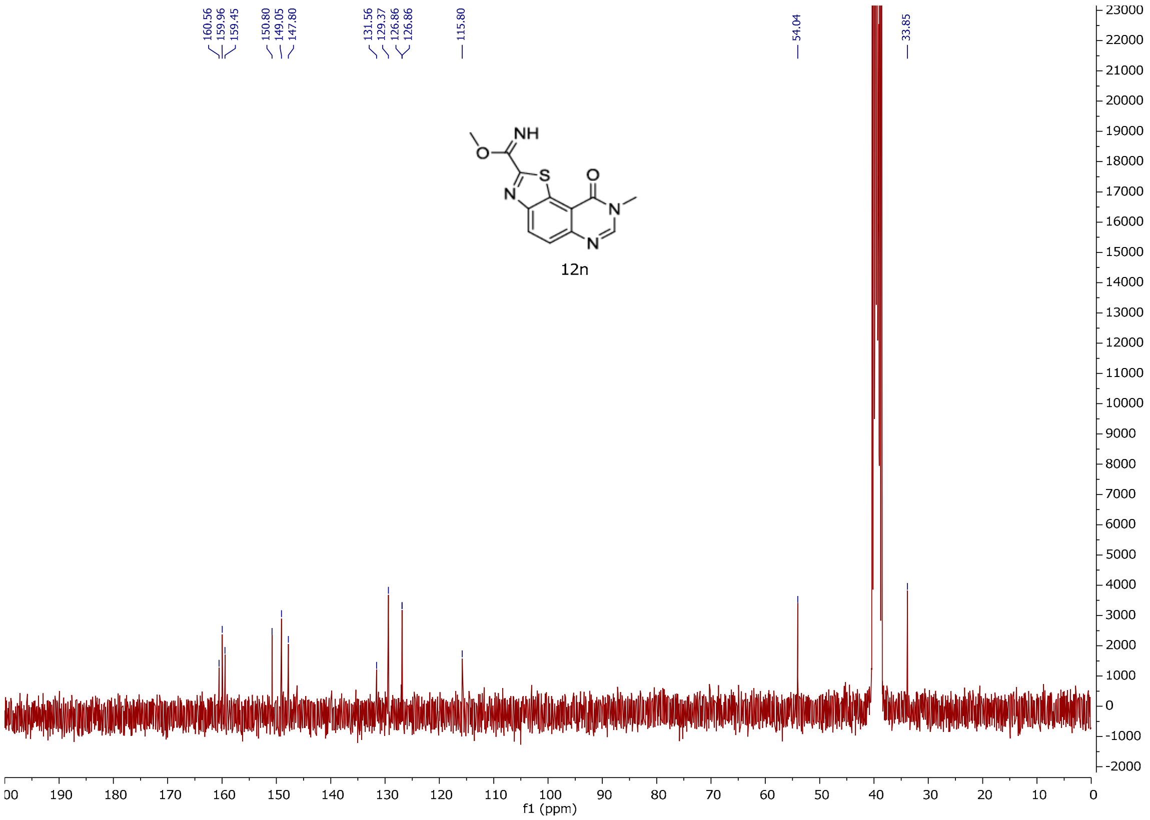

Figure S49. ^{13}C-NMR Compound **12n.**

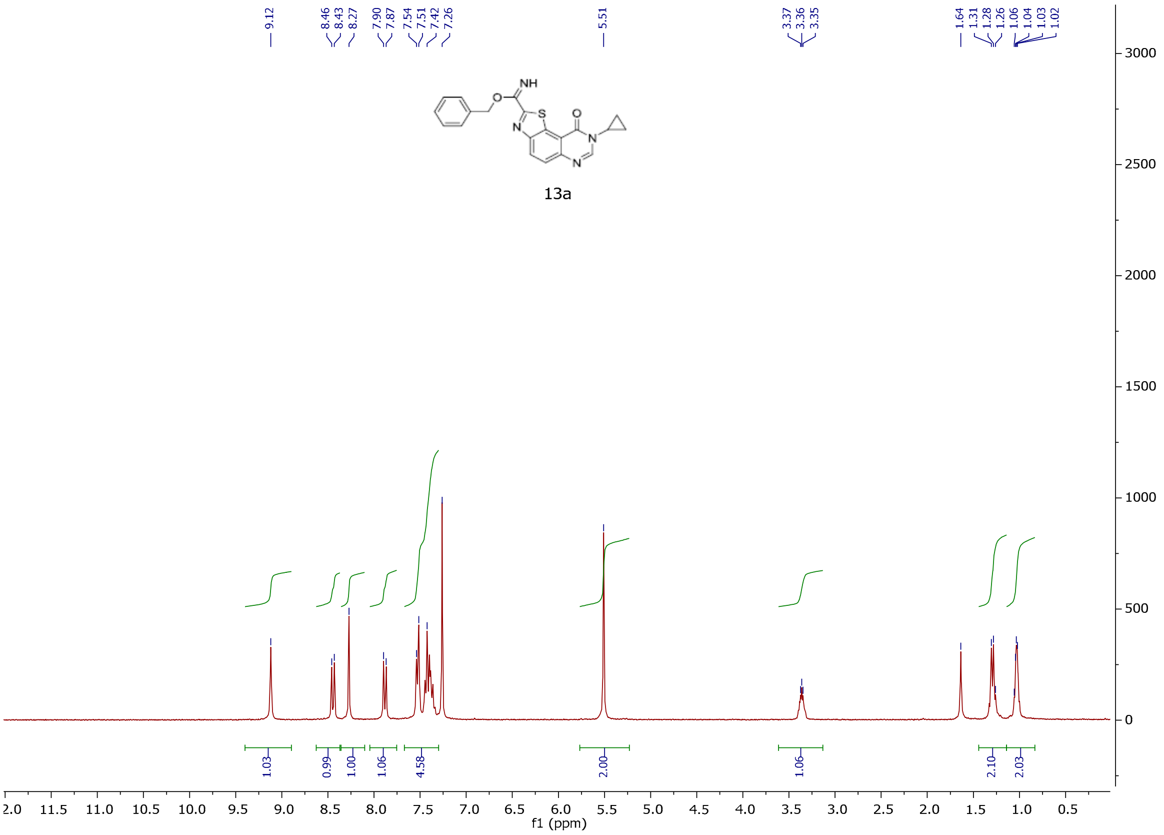

Figure S50. ^{1}H-NMR Compound **13a.**

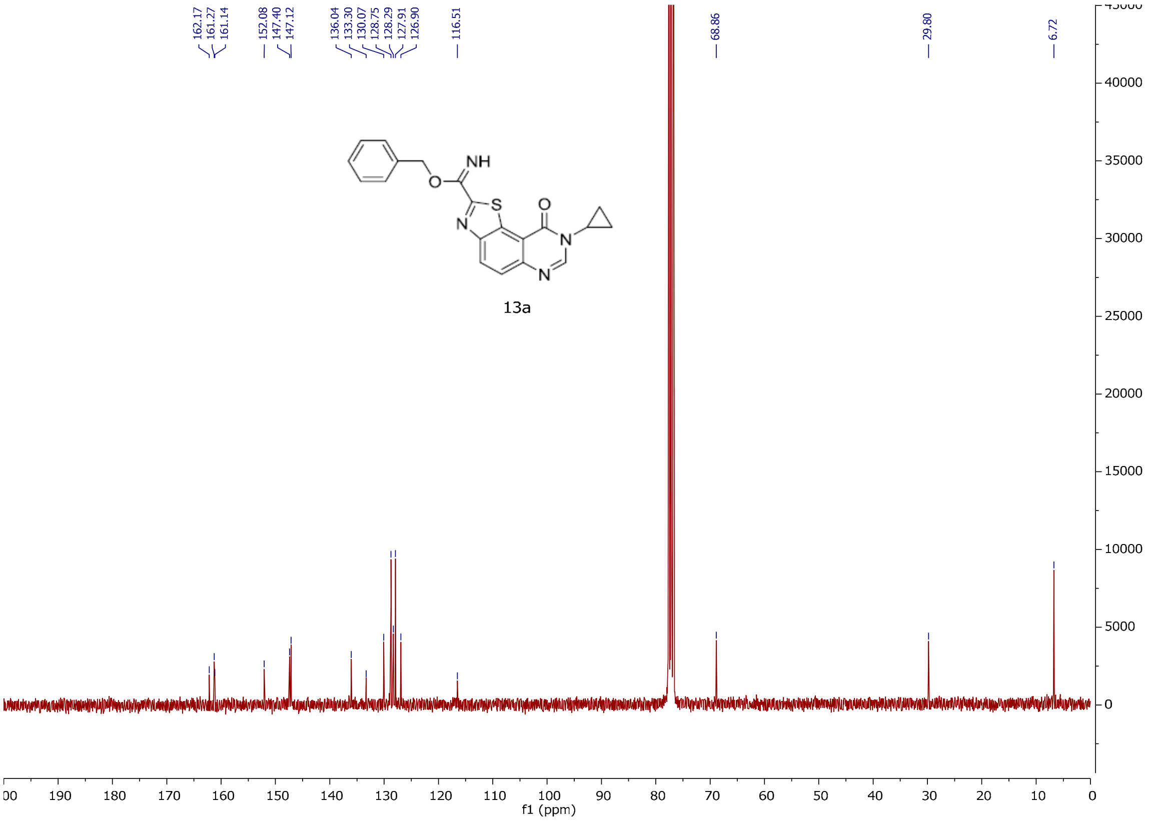

Figure S51. ^{13}C-NMR Compound **13a.**

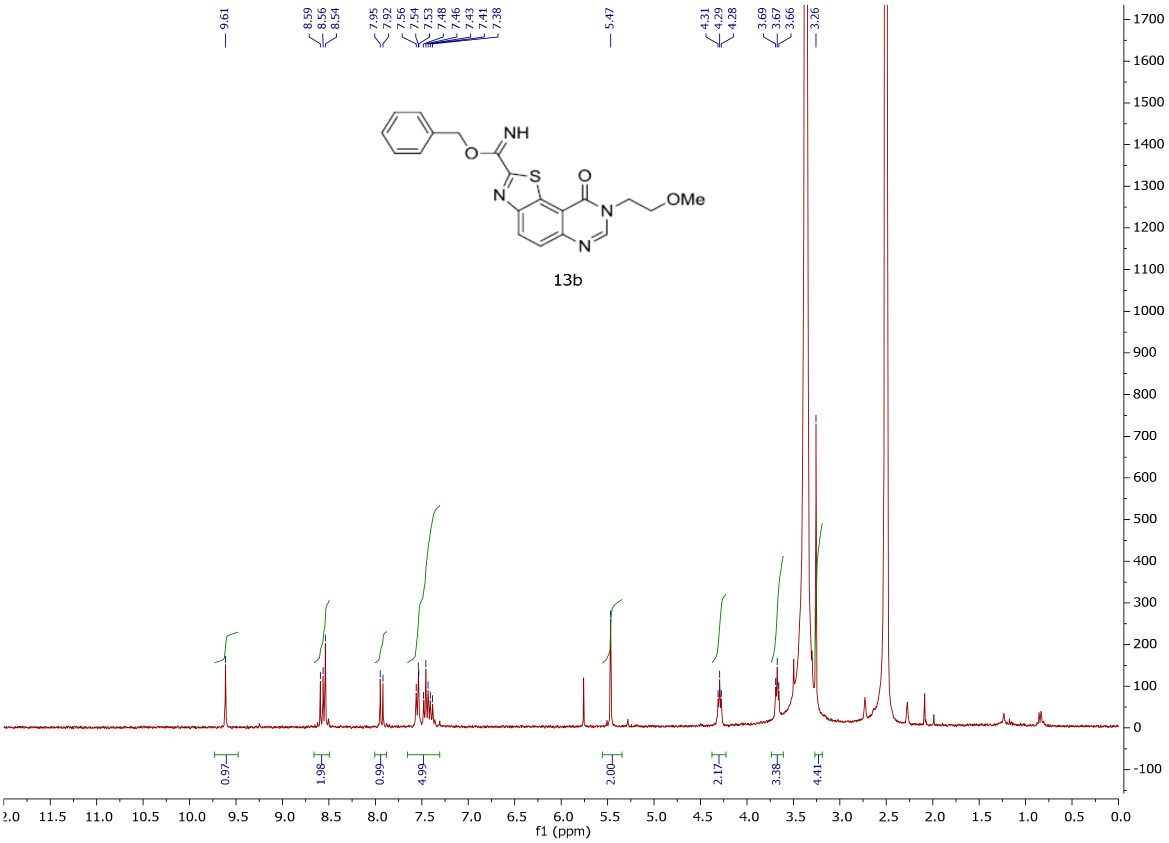

Figure S52. ¹H-NMR Compound **13b.**

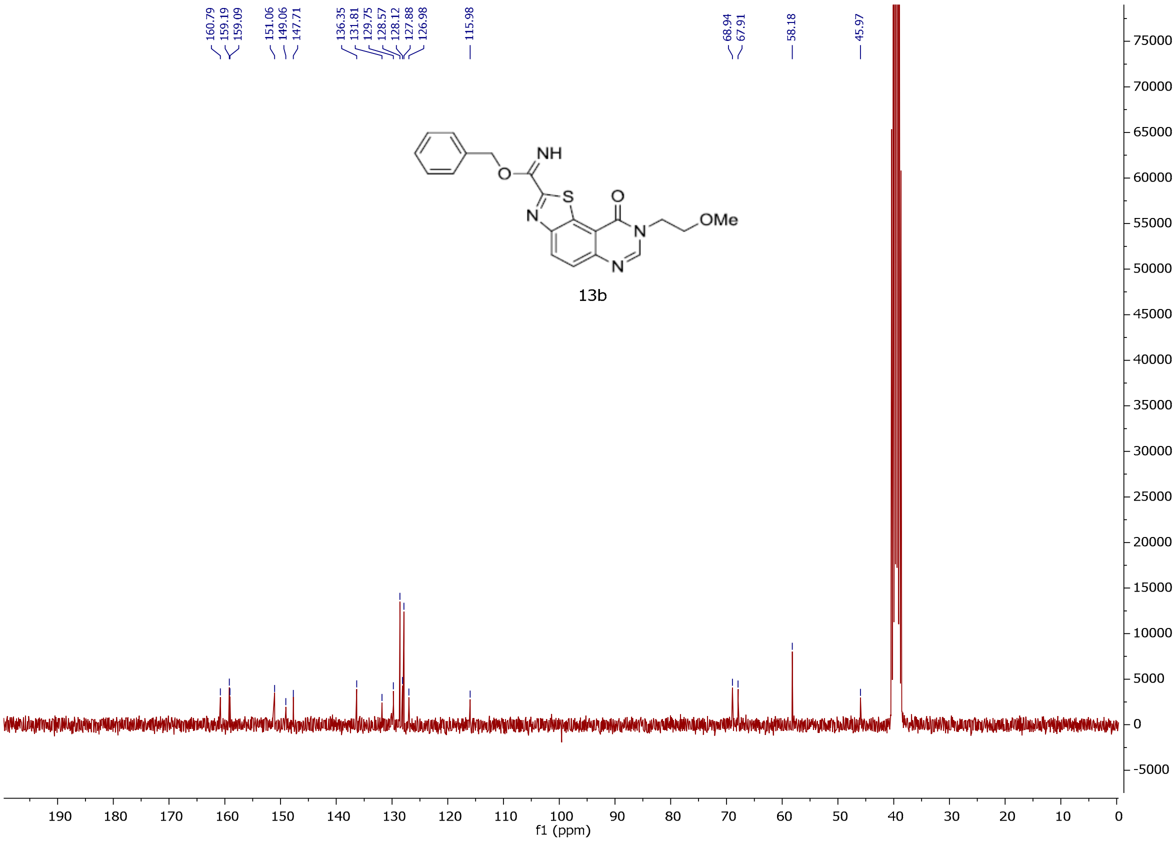

Figure S53. ¹³C-NMR Compound **13b.**

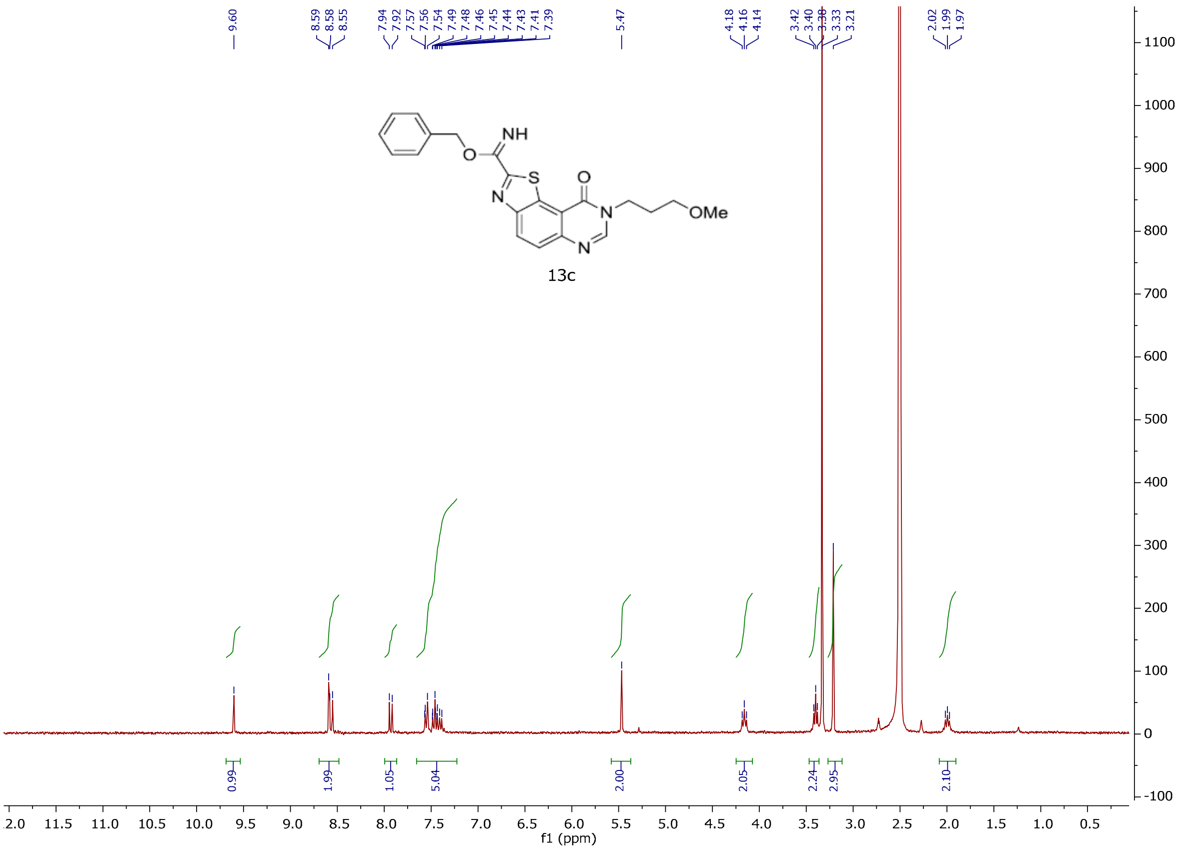

Figure S54. ^{1}H-NMR Compound **13c.**

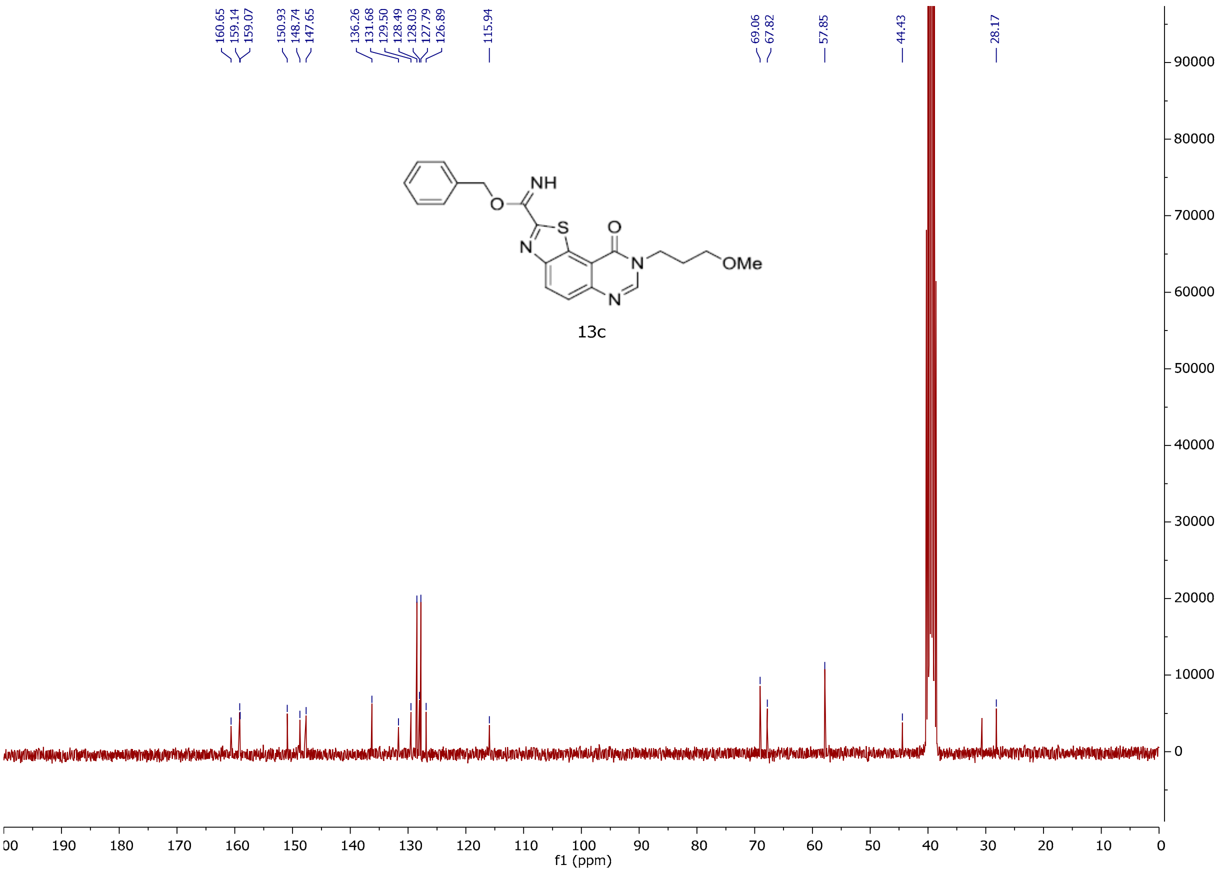

Figure S55. ^{13}C-NMR Compound **13c.**

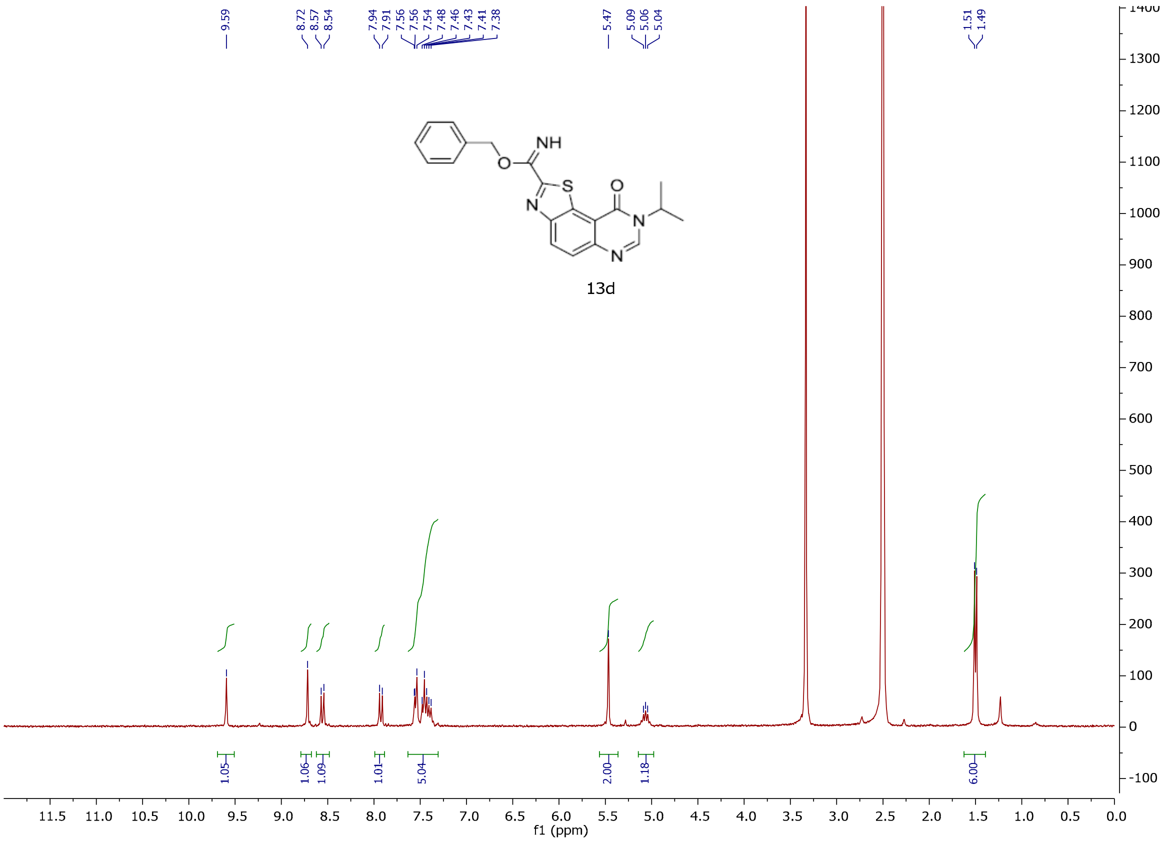

Figure S56. ^{1}H-NMR Compound **13d.**

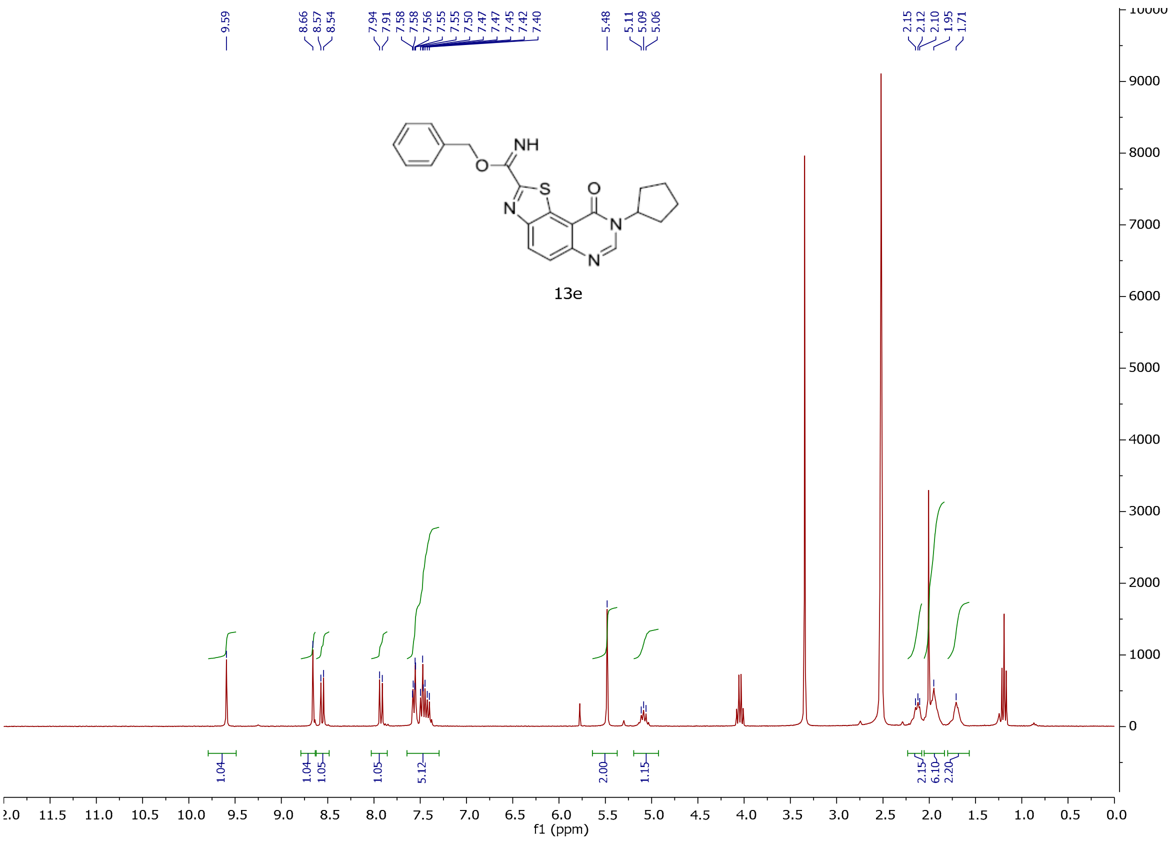

Figure S57. ^{1}H-NMR Compound **13e.**

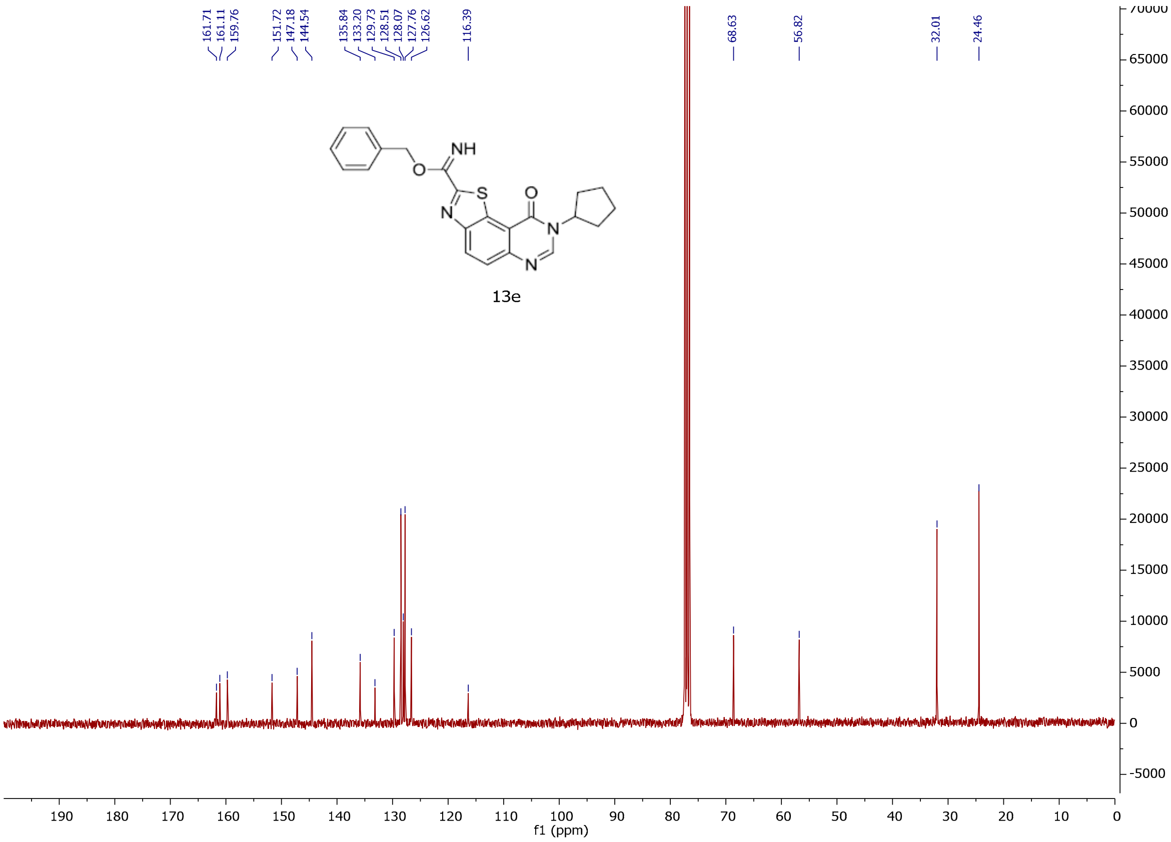

Figure S58. ^{13}C-NMR Compound **13e.**

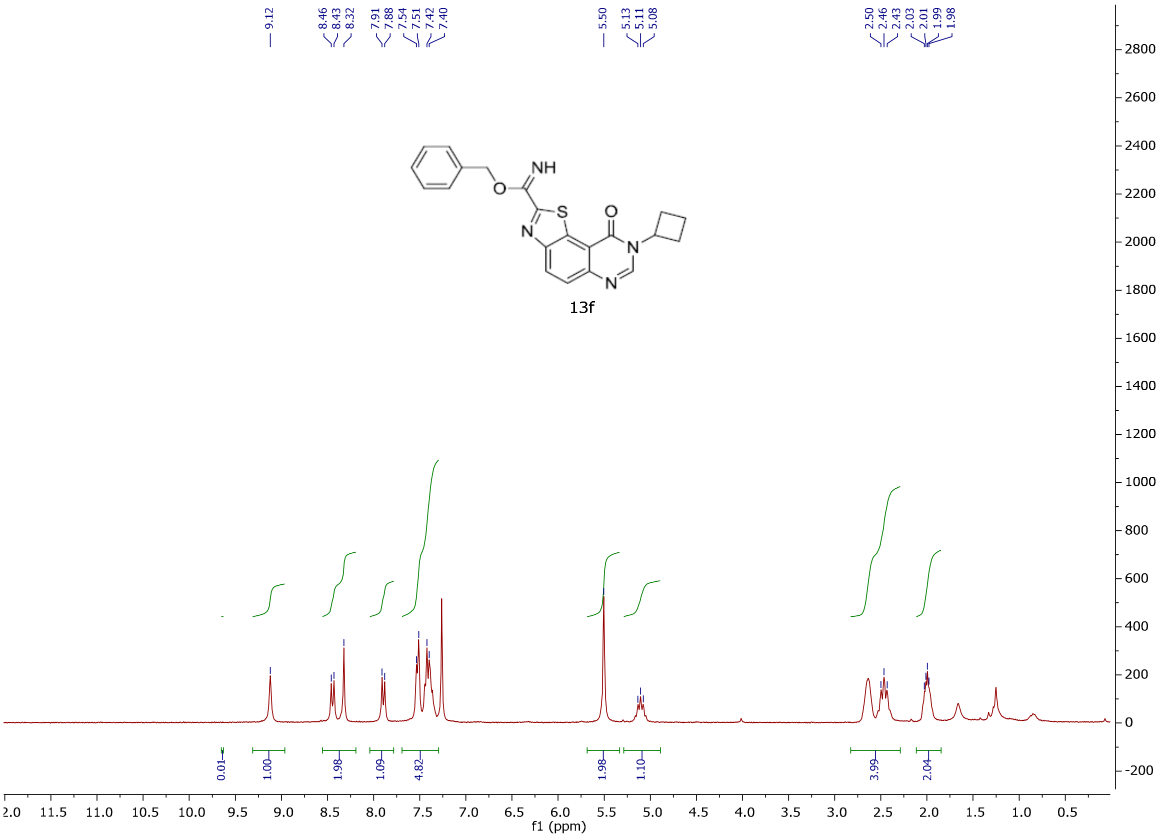

Figure S59. ^{1}H-NMR Compound **13f.**

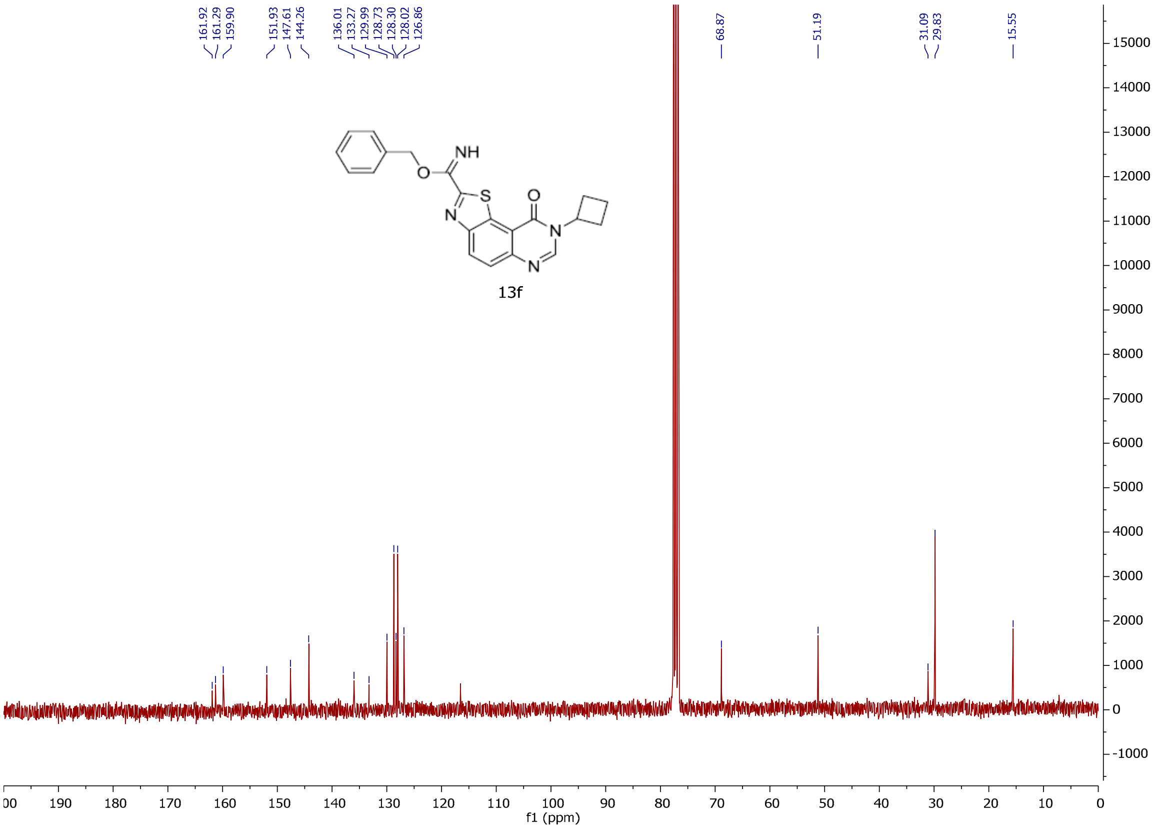

Figure S60. ^{13}C-NMR Compound **13f**

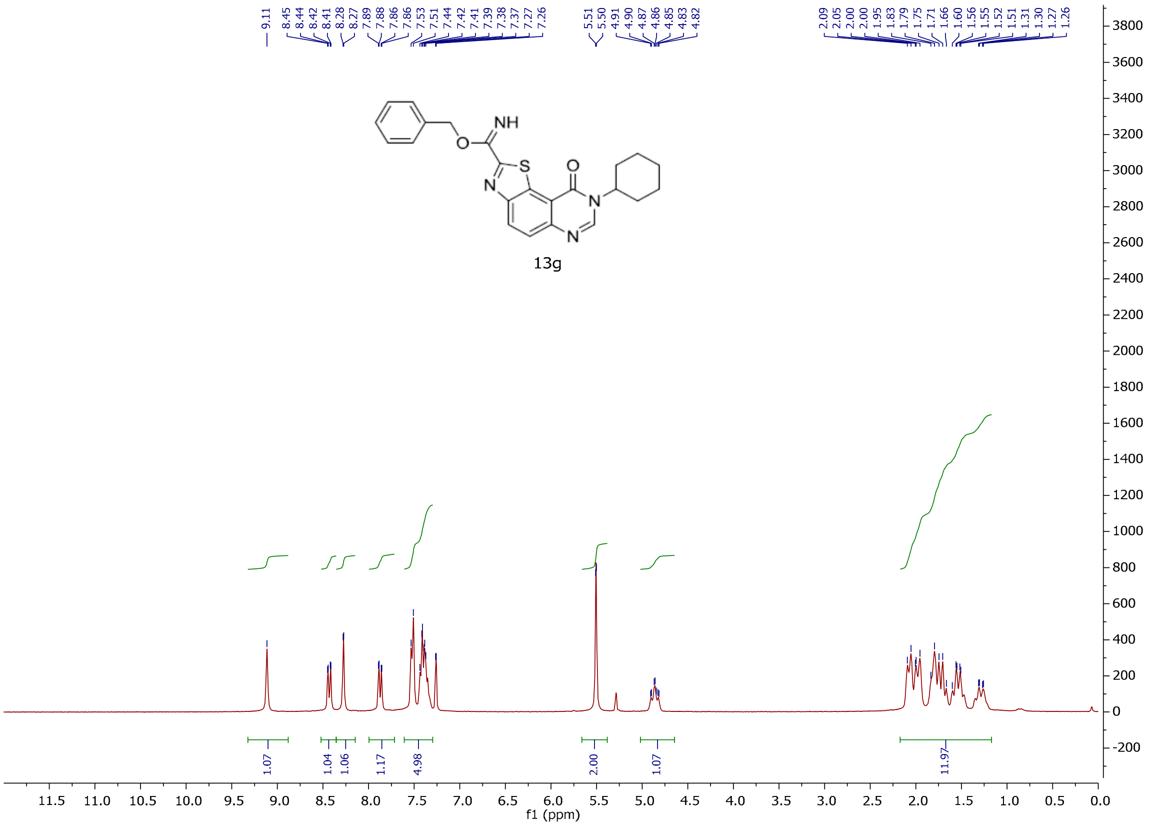

Figure S61. ^{1}H-NMR Compound **13g.**

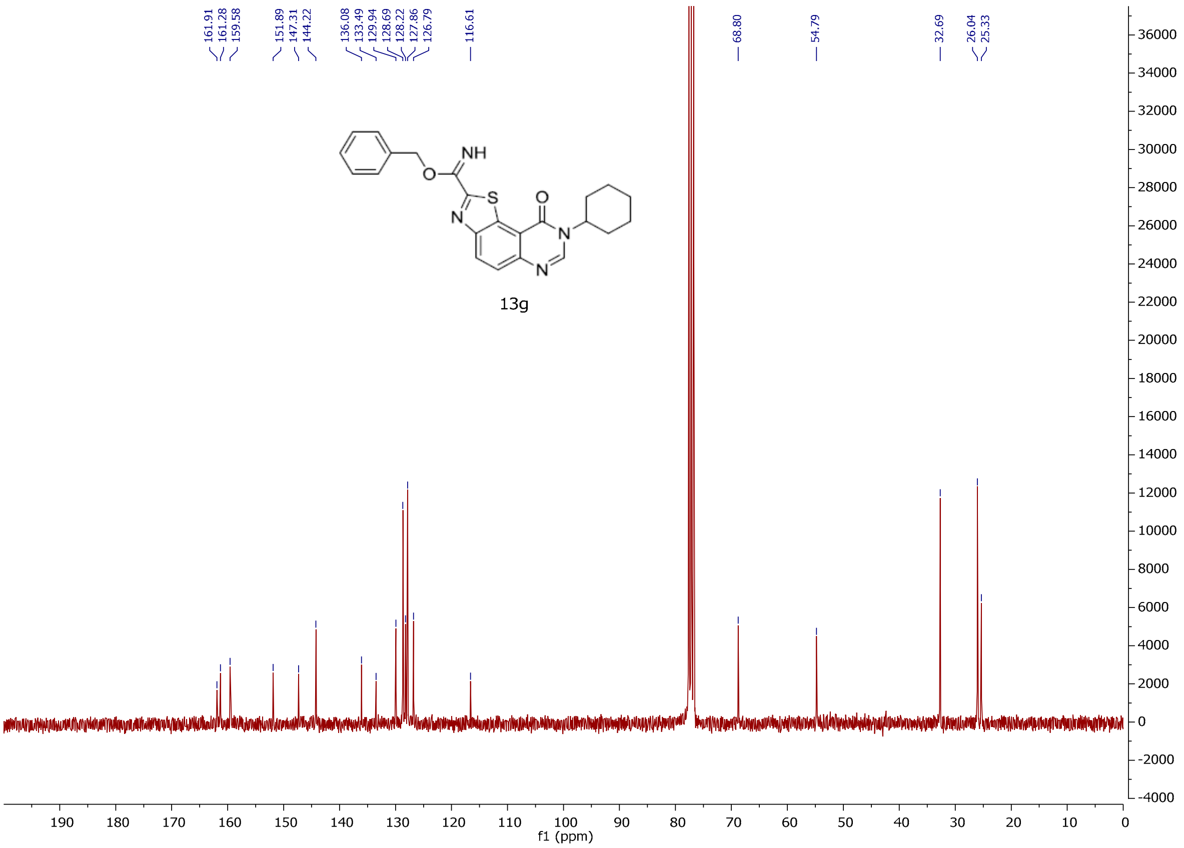

Figure S62. ^{13}C-NMR Compound **13g.**

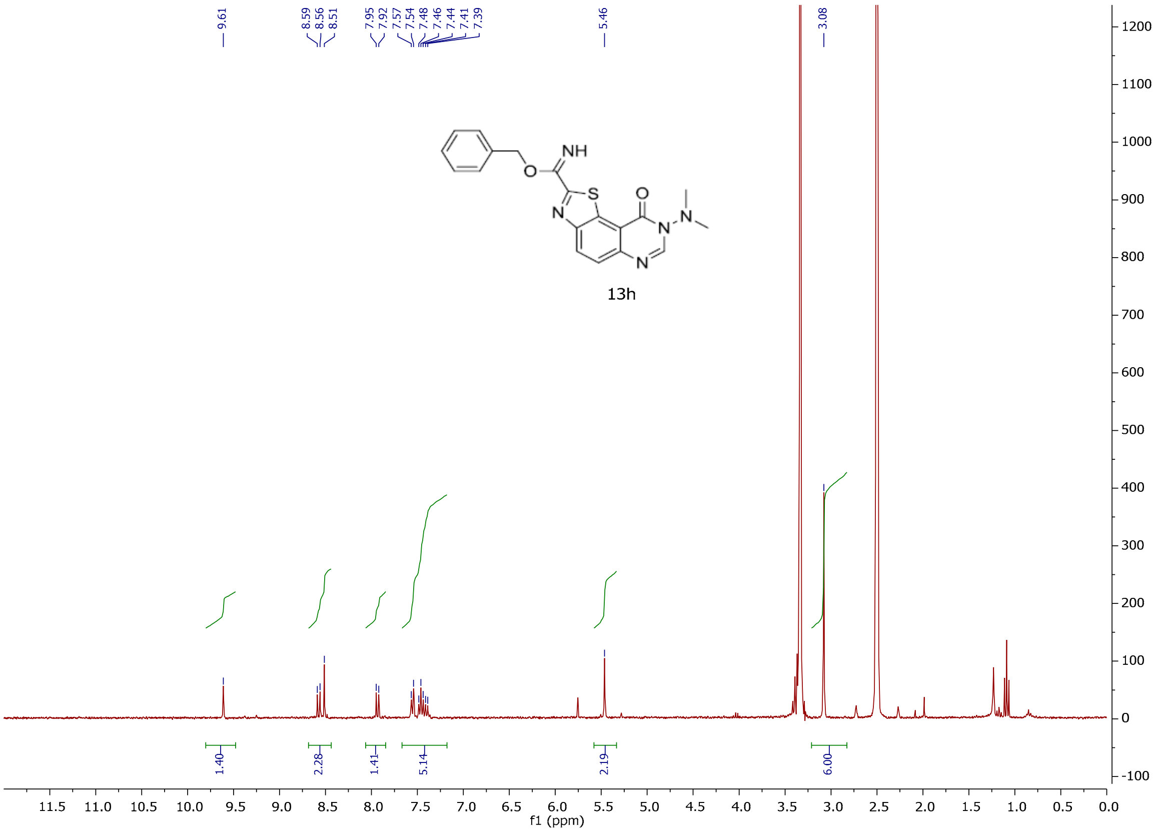

Figure S63. ^{1}H-NMR Compound **13h.**

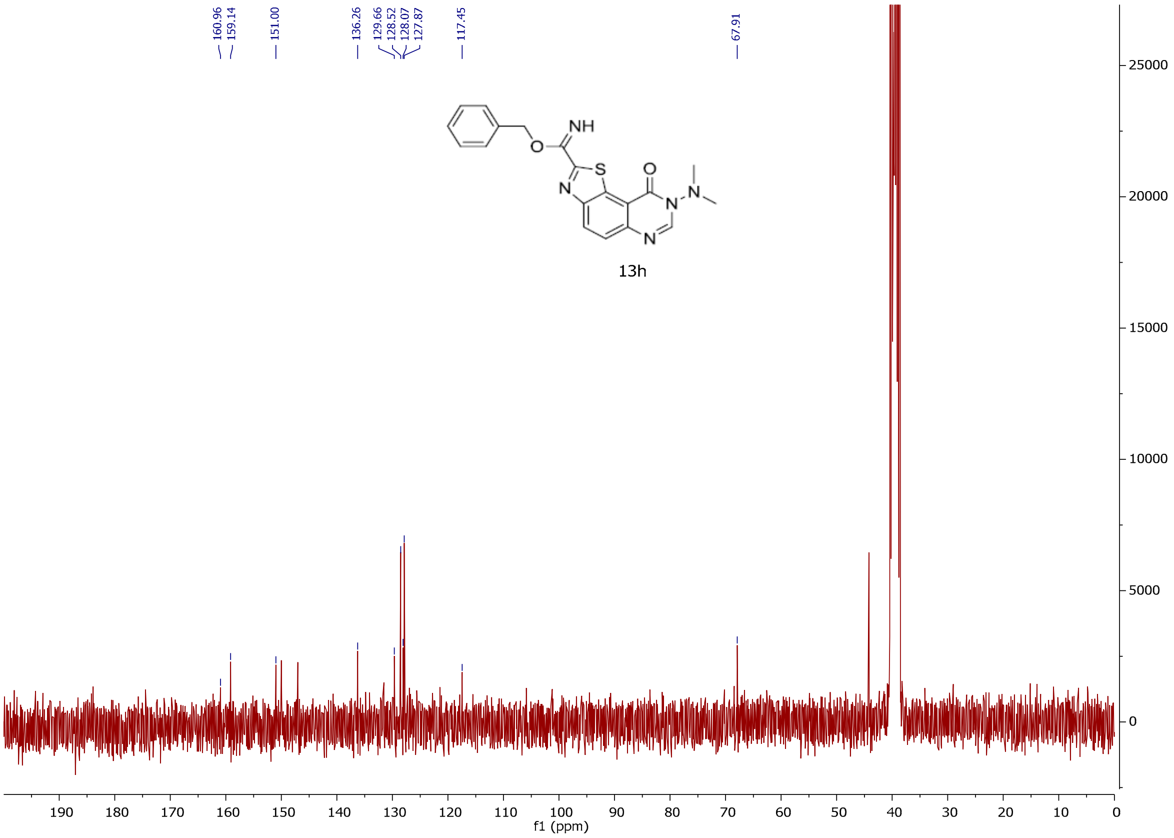

Figure S64. ^{13}C-NMR Compound **13h**.

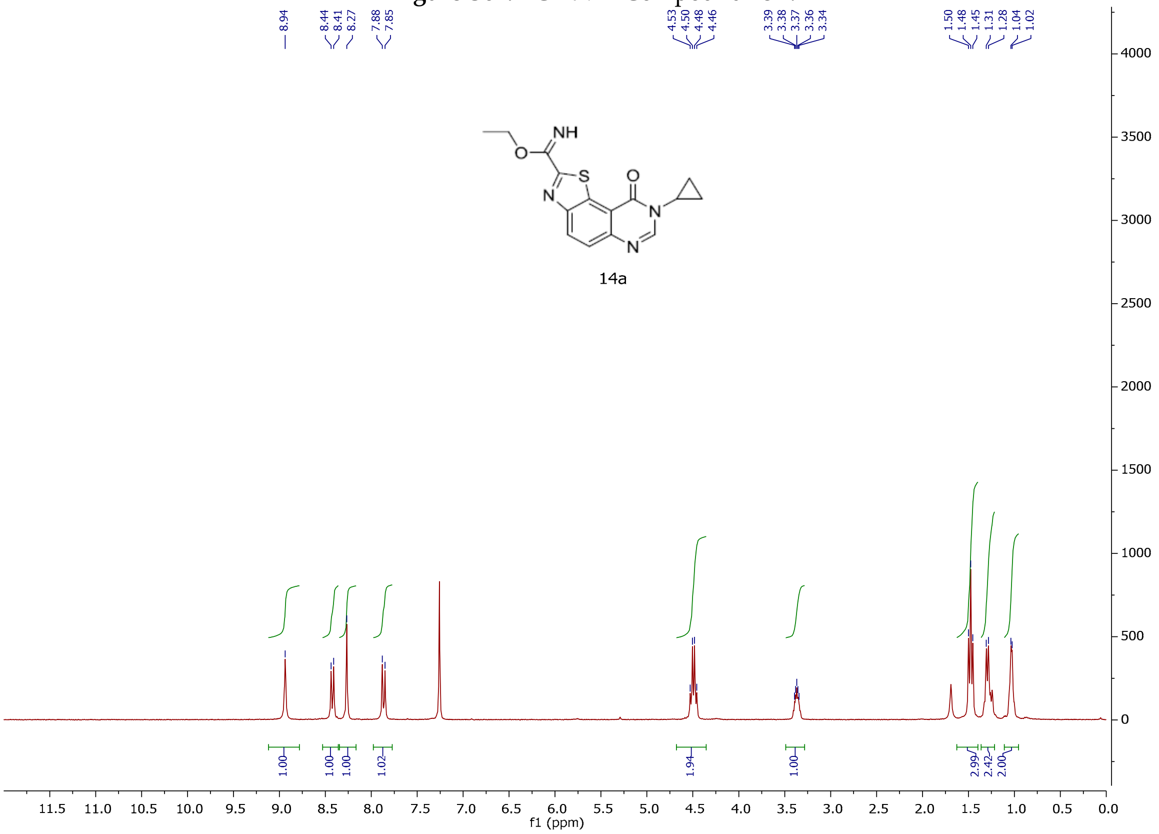

Figure S65. ^{1}H-NMR Compound **14a.**

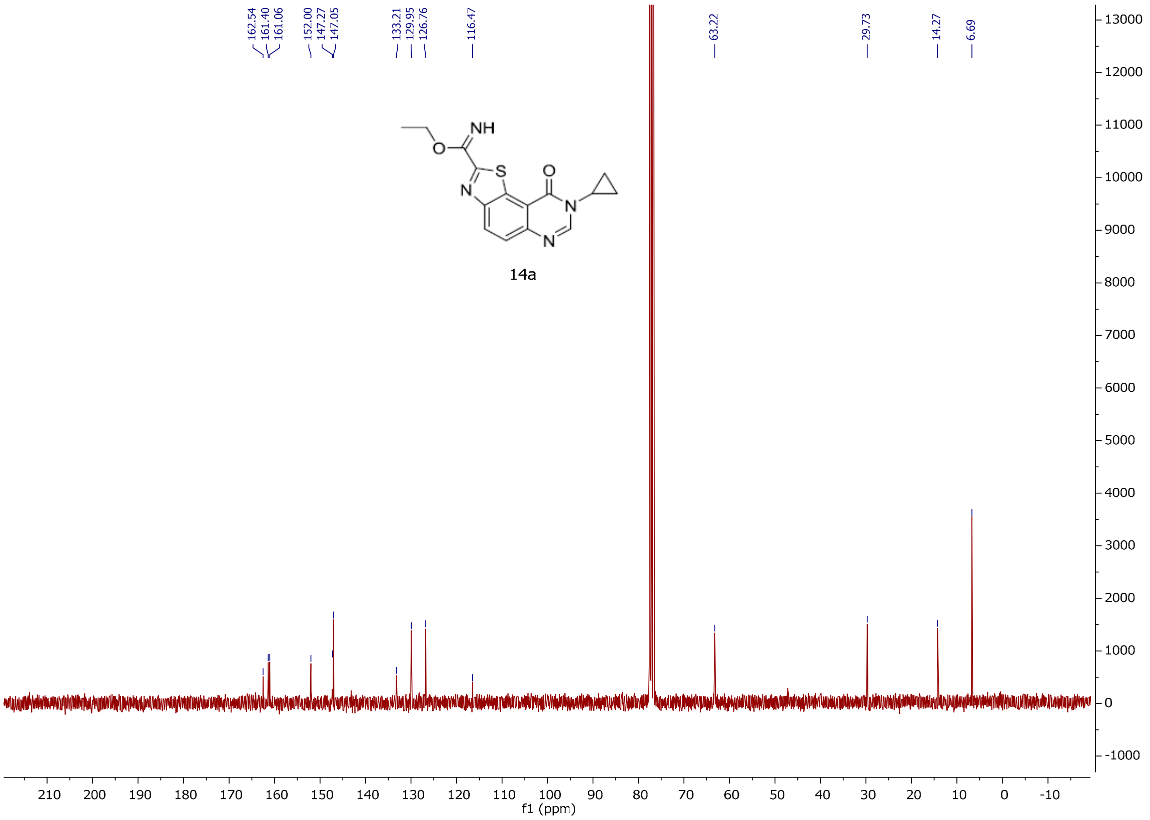

Figure S66. ^{1}H-NMR Compound **14a.**

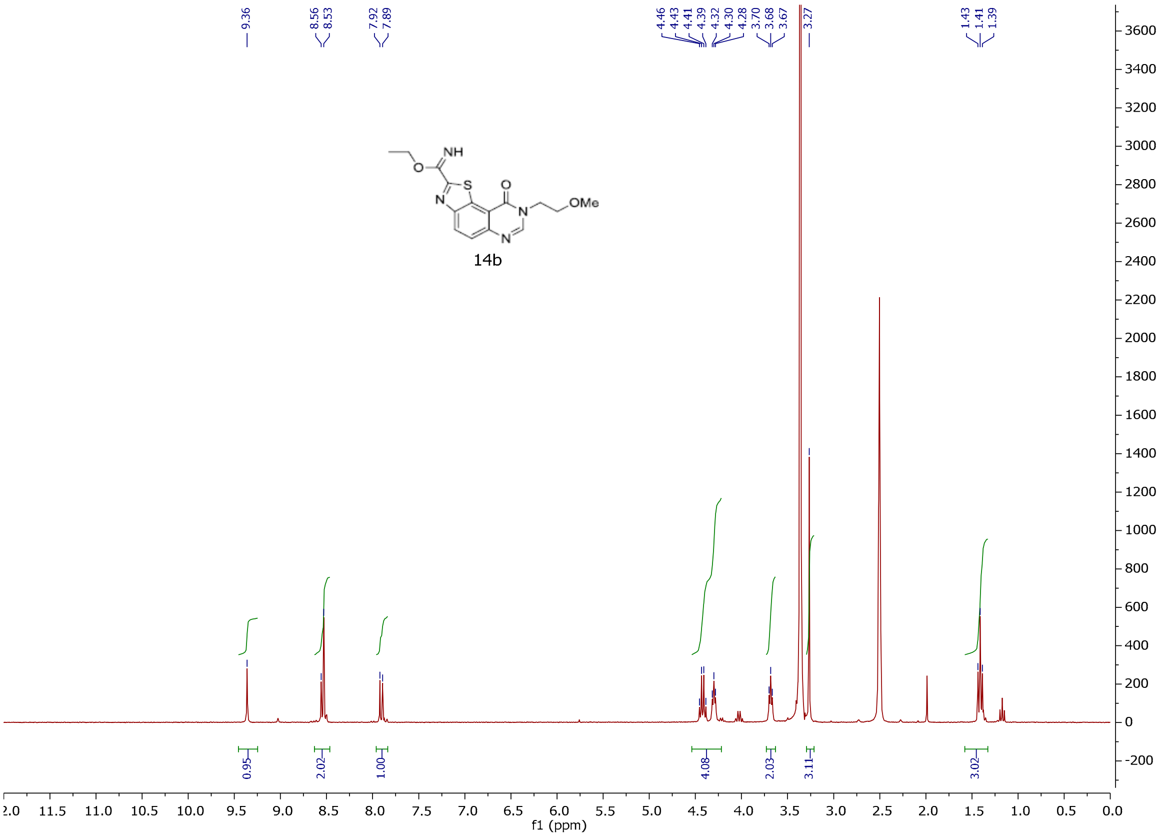

Figure S67. ^{1}H-NMR Compound **14b.**

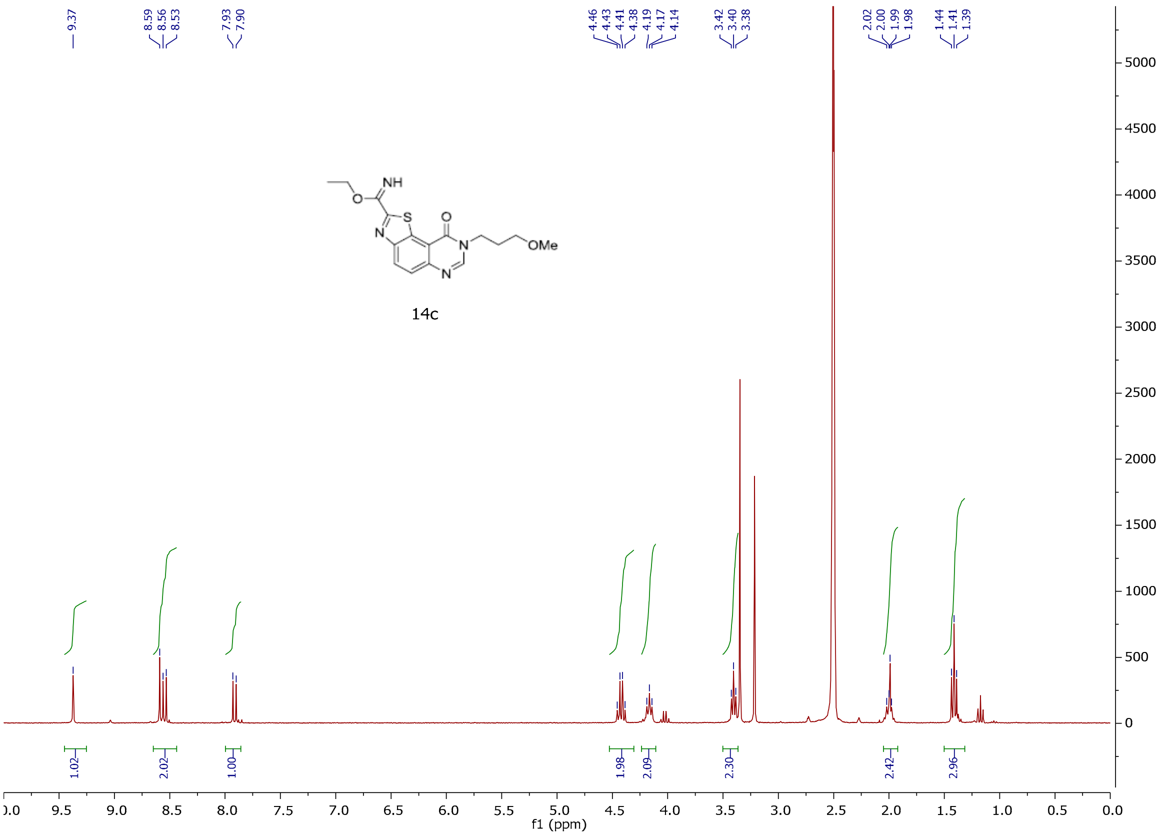

Figure S68. ^{1}H-NMR Compound **14c.**

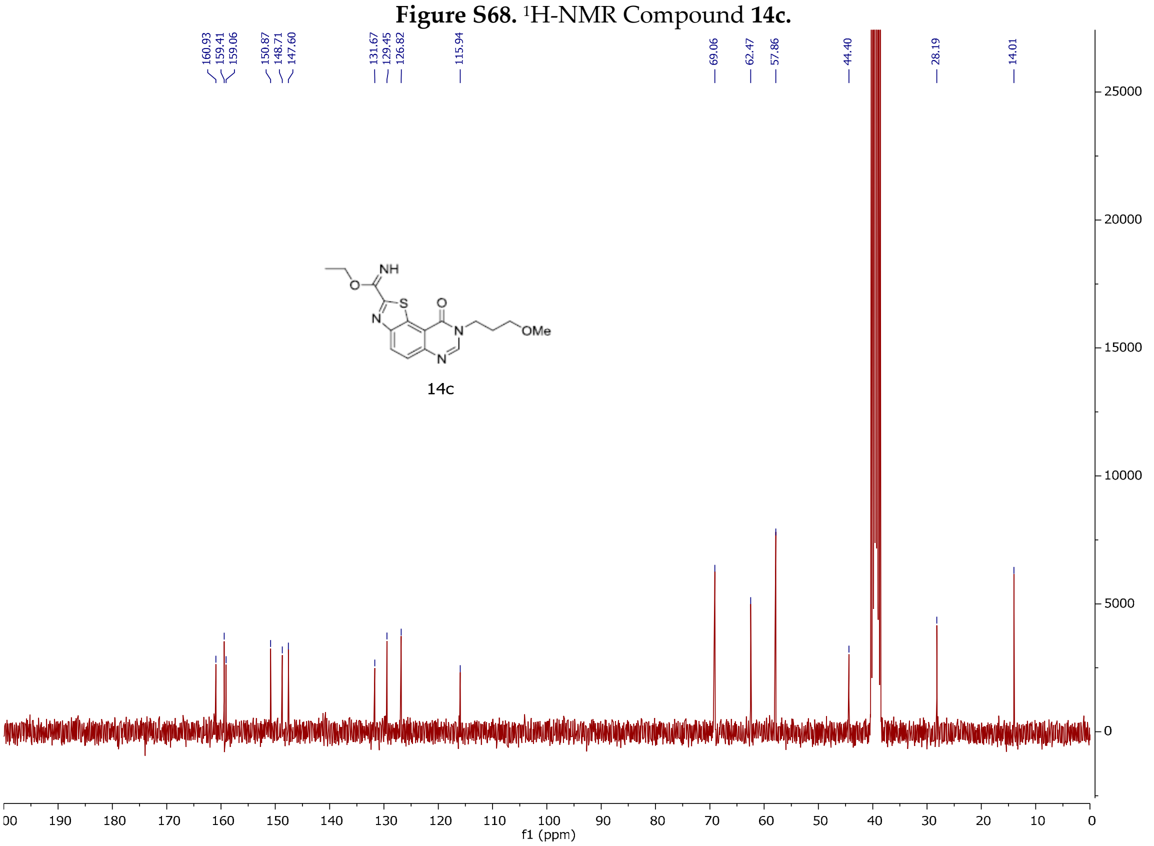

Figure S69. ^{13}C-NMR Compound **14c.**

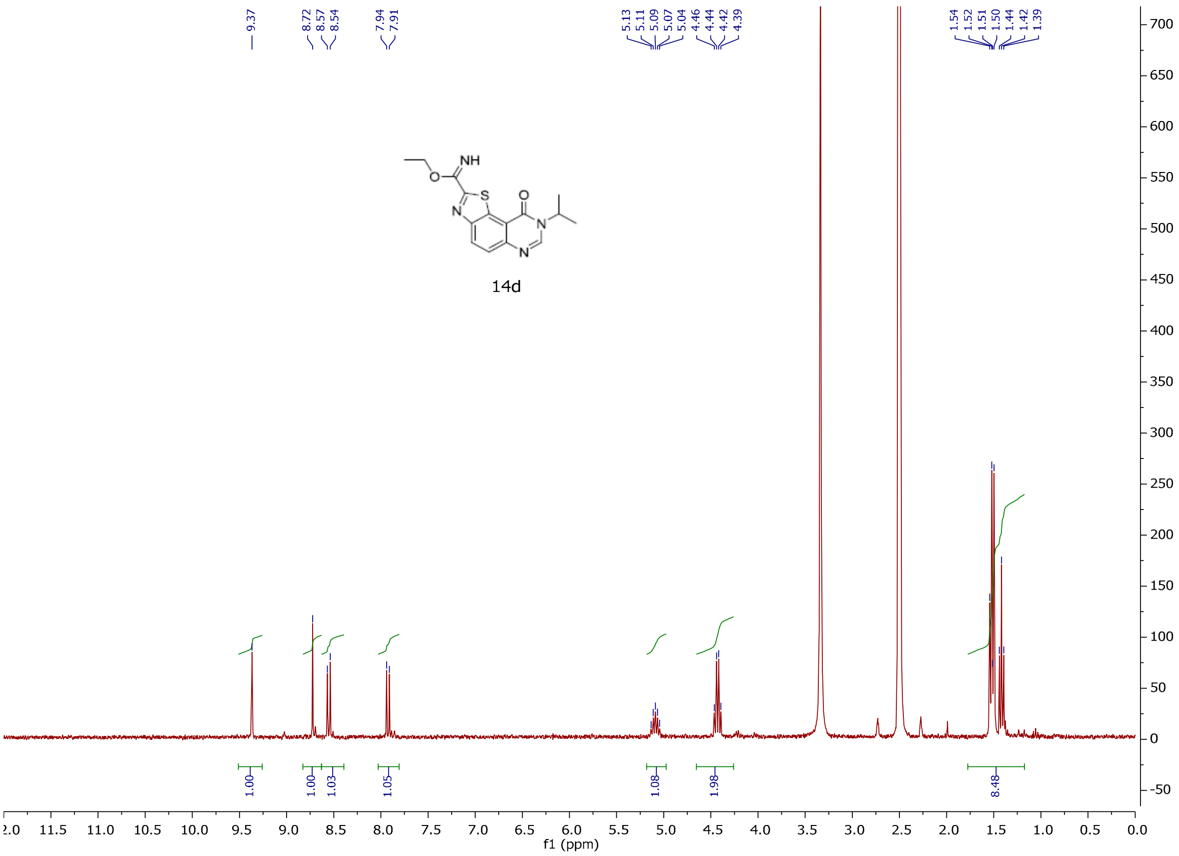

Figure S70. ^{1}H-NMR Compound **14d.**

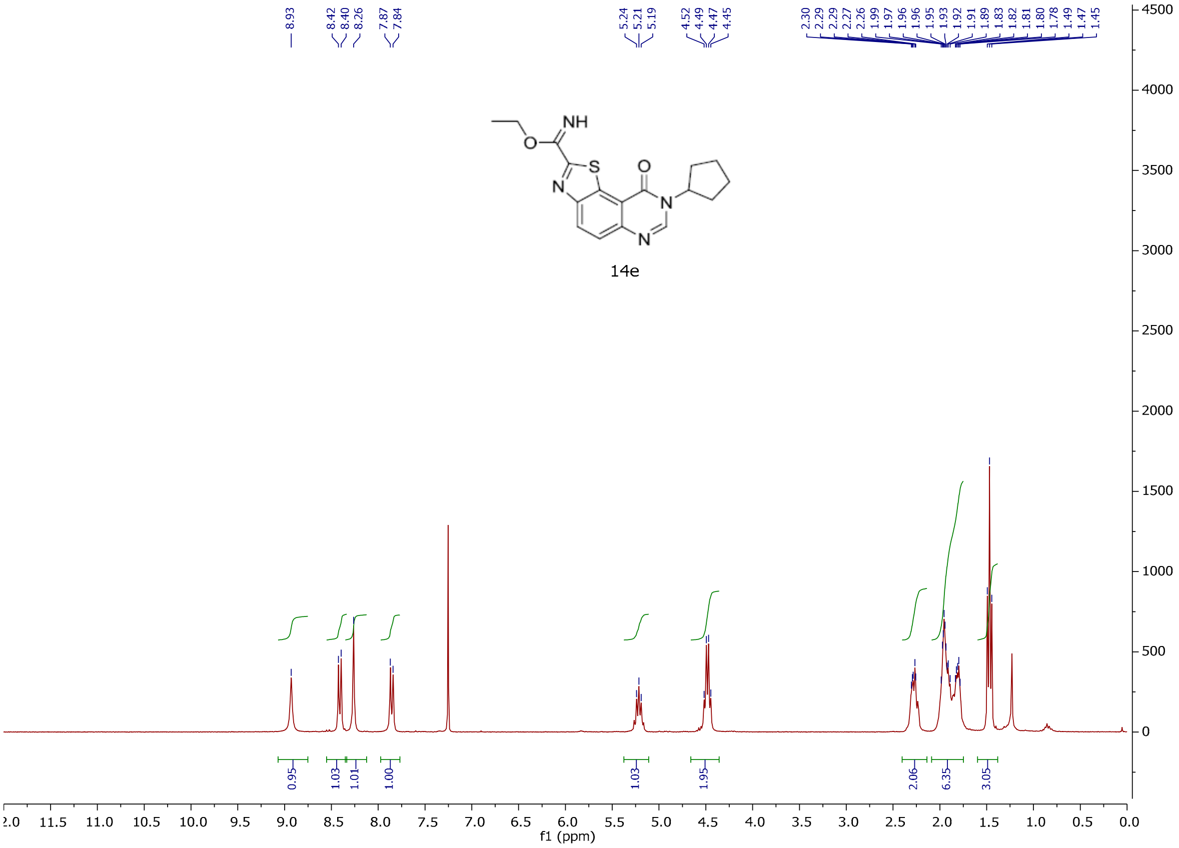

Figure S71. ^{1}H-NMR Compound **14e.**

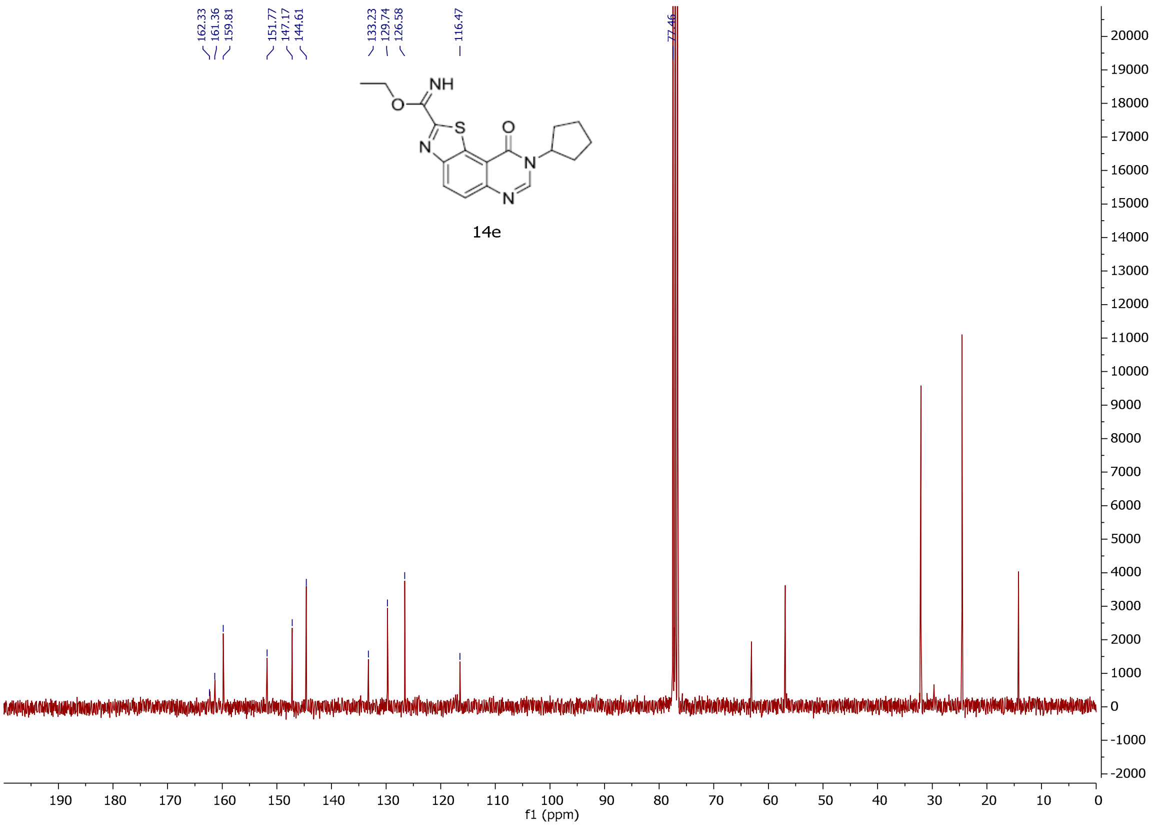

Figure S72. ^{13}C-NMR Compound **14e.**

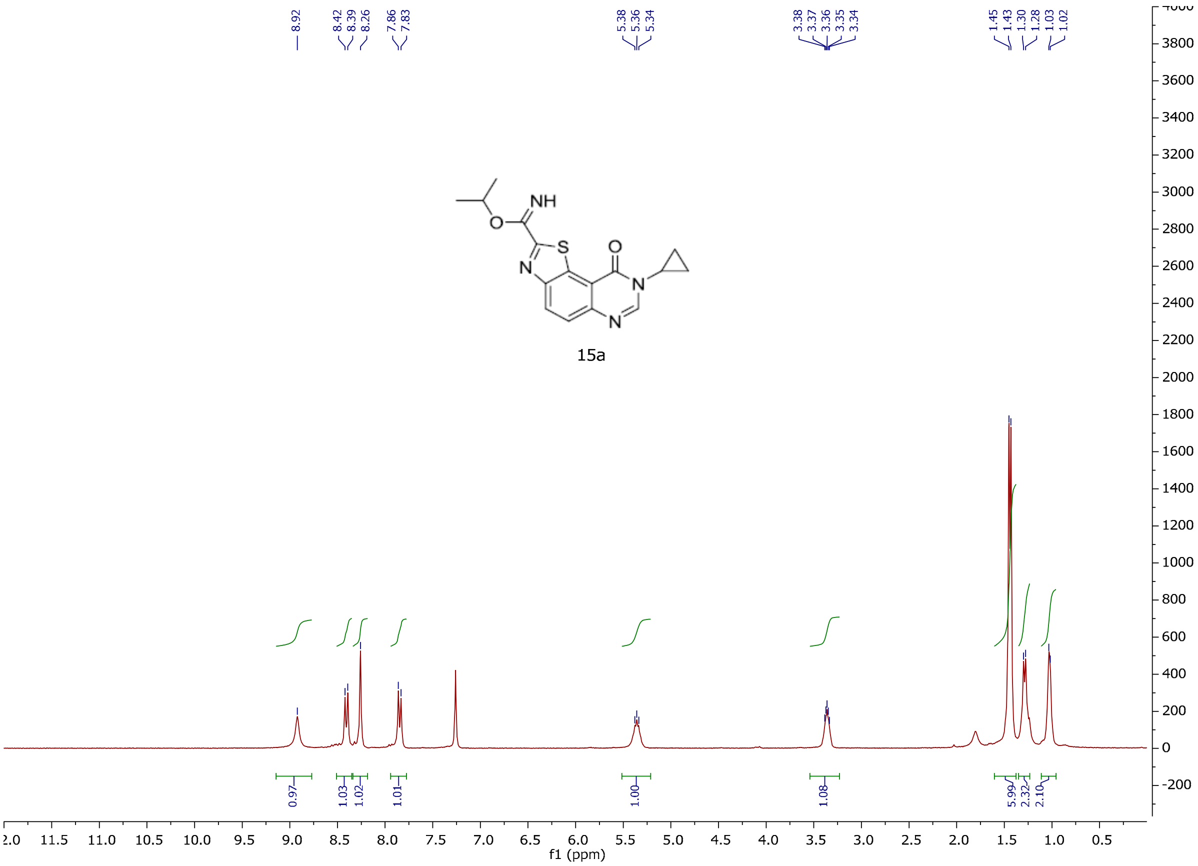

Figure S73. ^{1}H-NMR Compound **15a.**

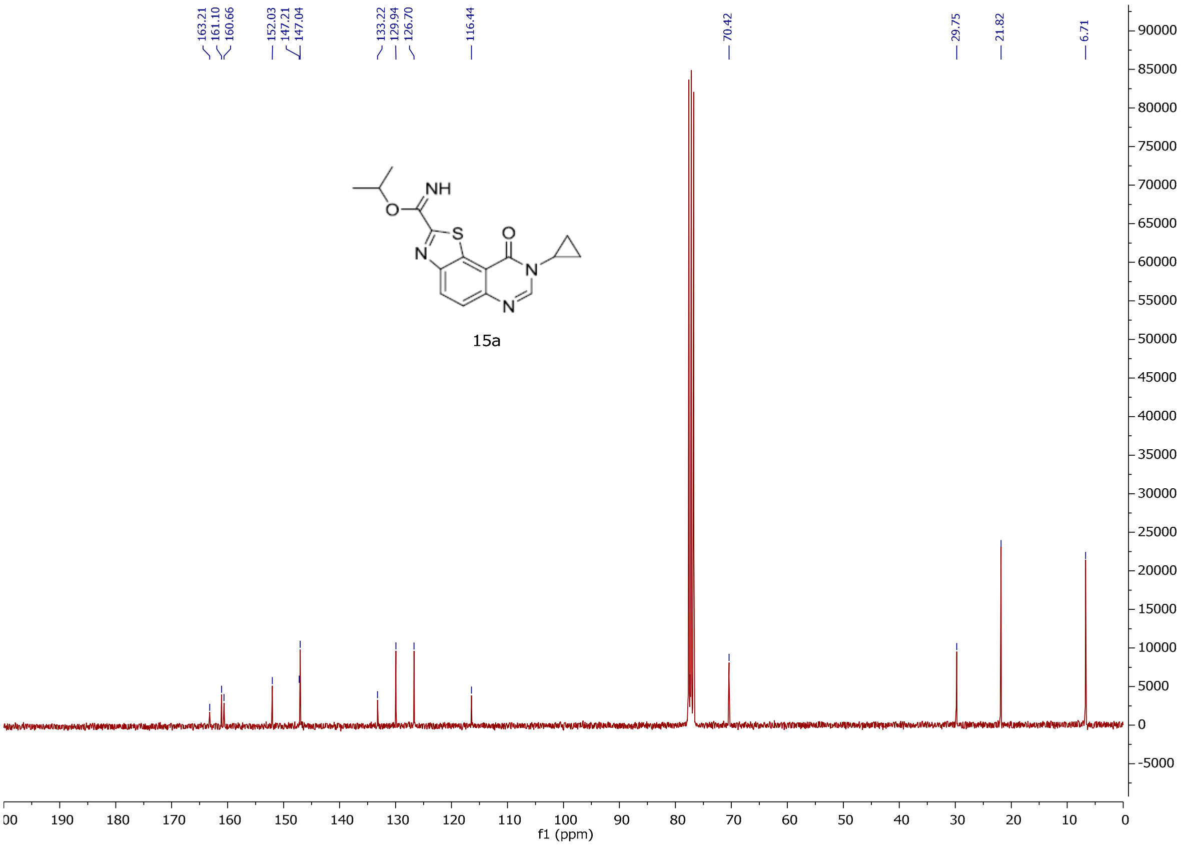

Figure S74. ^{13}C-NMR Compound **15a.**

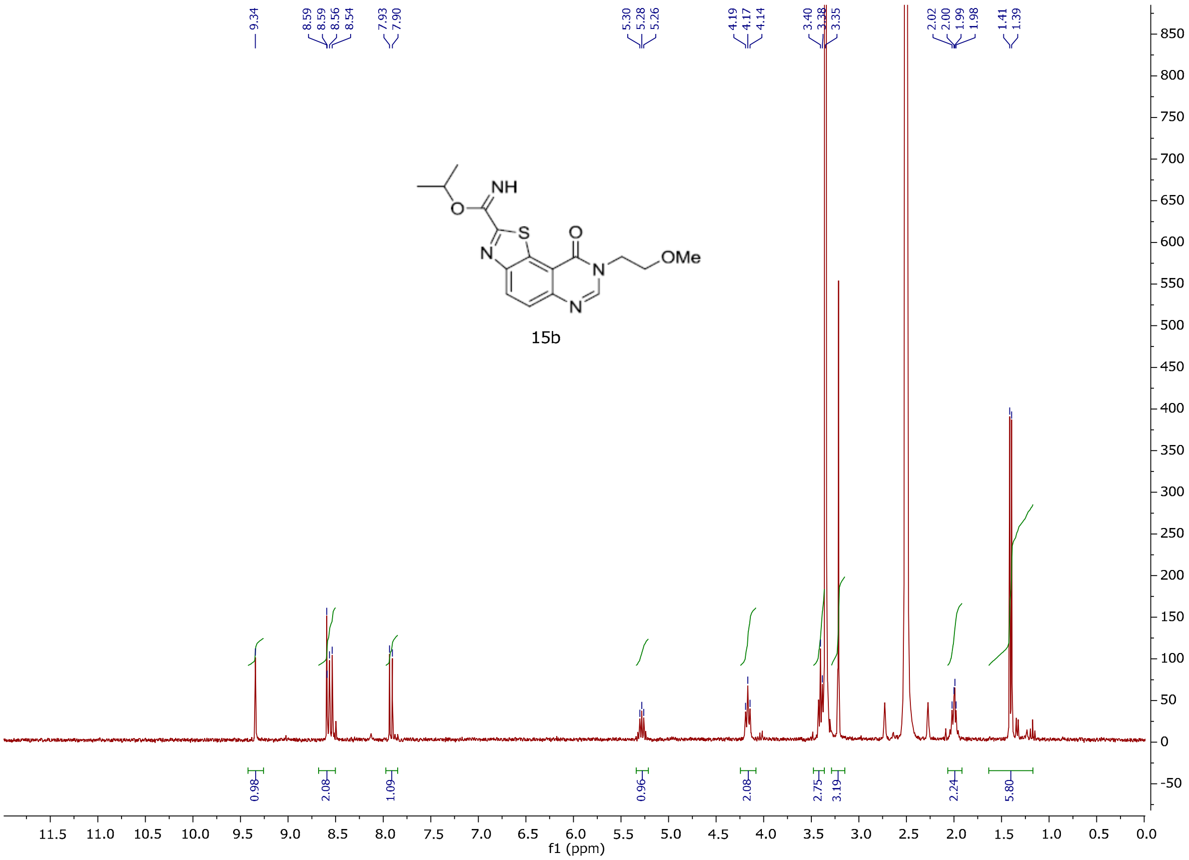

Figure S75. ^{1}H-NMR Compound **15b.**

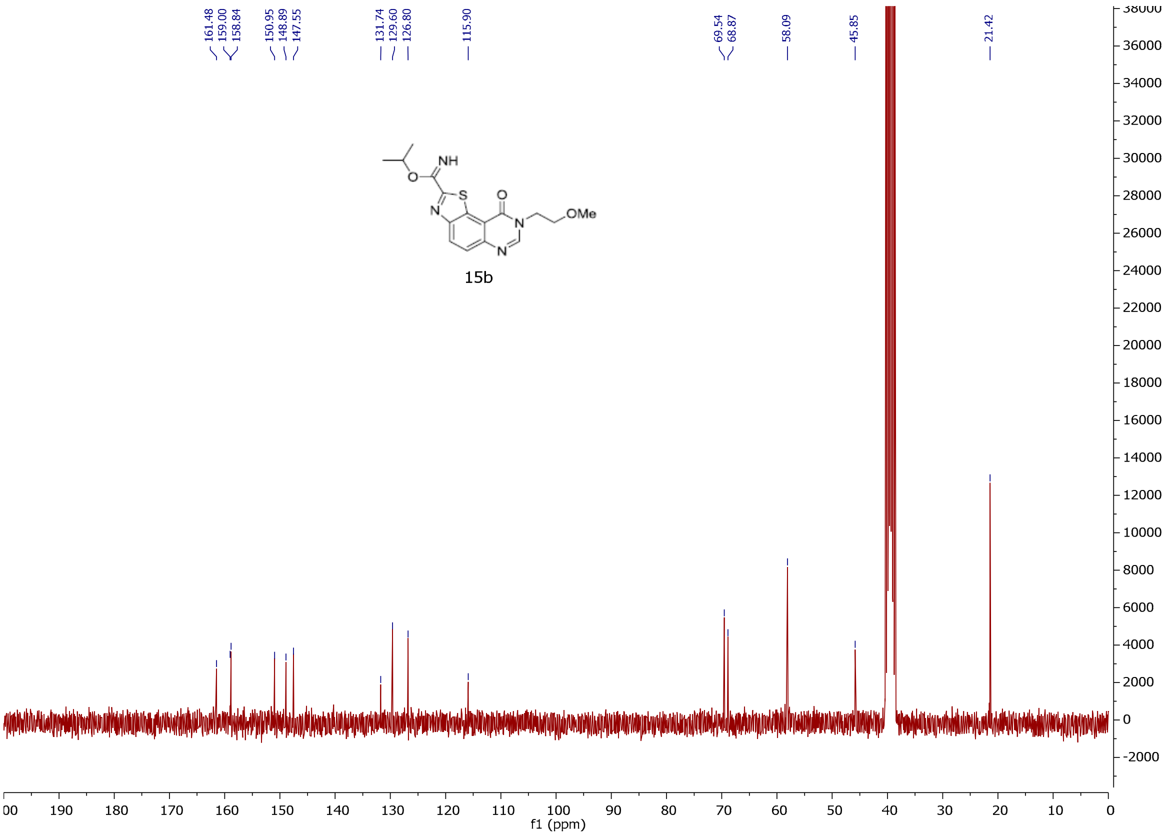

Figure S76. ^{13}C-NMR Compound **15b.**

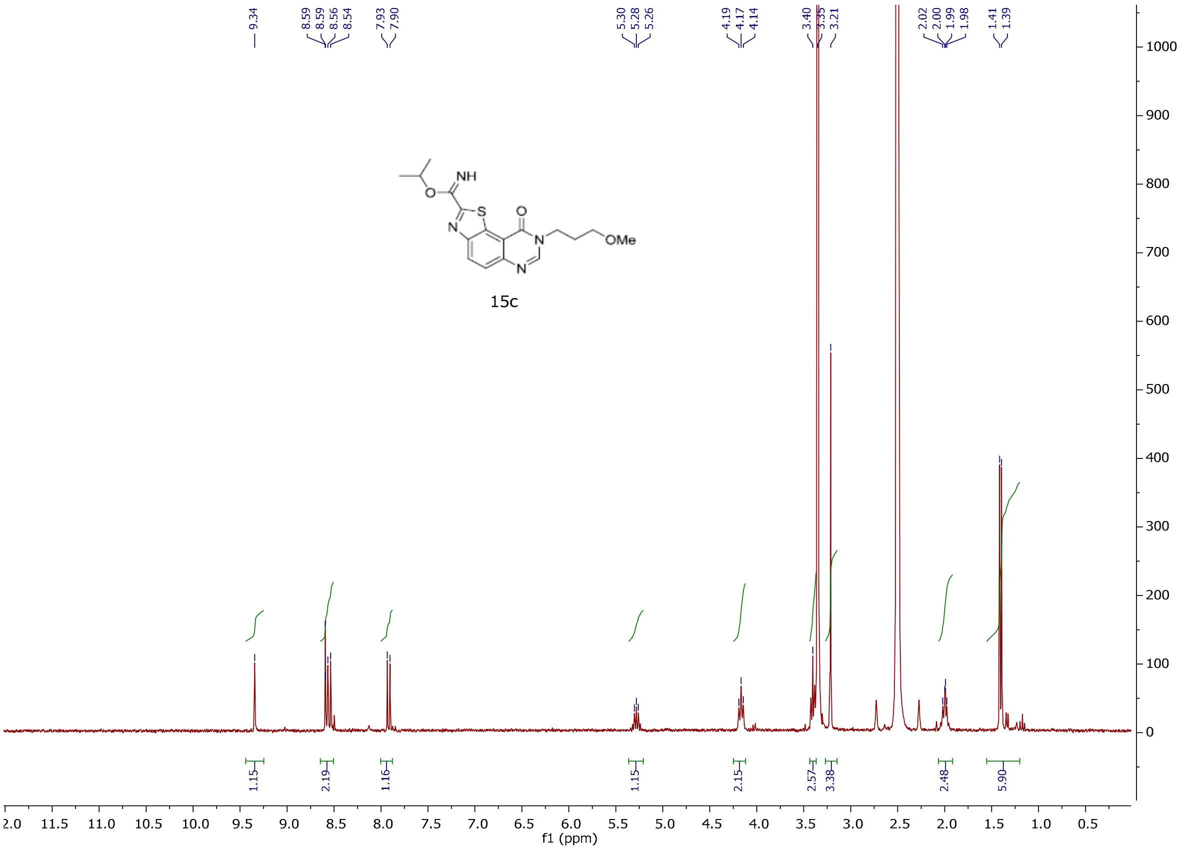

Figure S77. ^{1}H-NMR Compound **15c.**

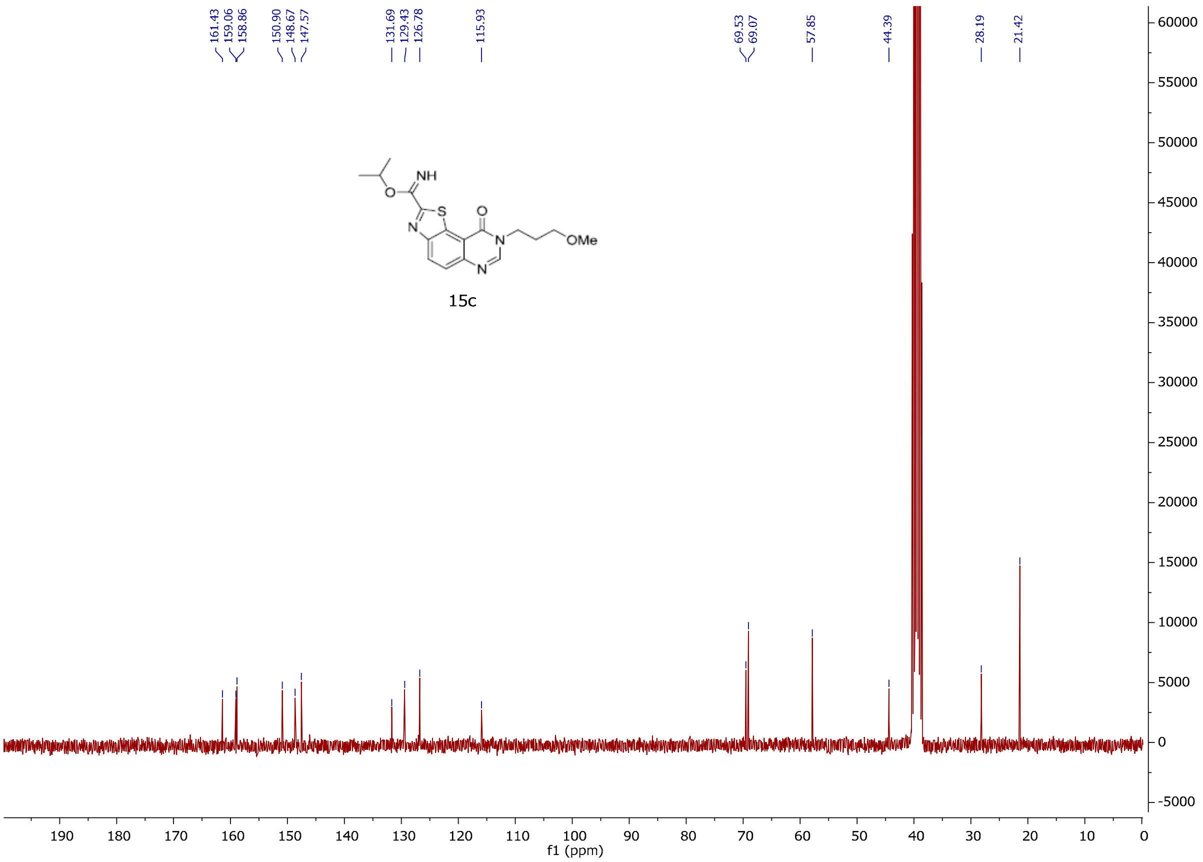

Figure S80. ^{13}C-NMR Compound **15c.**
